# Integrative Pan‐Cancer Characterization of lncRNA UPK1A‐AS1 and Its Role in Hypoxia‐Associated Sorafenib Resistance in Hepatocellular Carcinoma

**DOI:** 10.1155/ancp/1554526

**Published:** 2026-09-01

**Authors:** Ze-Kai Li, Min-Rui Luo, Shu-Jie Fang, Yi-Da Wu, Quan-Hui Hu, Dong-Yan Zhang

**Affiliations:** ^1^ Department of Radiation Oncology, Nanfang Hospital, Southern Medical University, Guangzhou 510515, Guangdong Province, China, fimmu.com; ^2^ Department of Health Management, Nanfang Hospital, Southern Medical University, Guangzhou 510515, Guangdong Province, China, fimmu.com

**Keywords:** hepatocellular carcinoma, hypoxia, long noncoding RNA, pan-cancer analysis, sorafenib resistance, UPK1A-AS1

## Abstract

Long noncoding RNAs (lncRNAs) are emerging as critical regulators of tumor initiation and progression through transcriptional and posttranscriptional mechanisms. UPK1A antisense RNA 1 (*UPK1A-AS1*), a cancer‐associated lncRNA, has been reported to participate in oncogenic processes; however, its overall landscape across human malignancies and its biological role in therapy resistance remain poorly understood. Given the increasing importance of identifying functional lncRNAs with prognostic and therapeutic potential, this study presents a comprehensive multiomics characterization of *UPK1A-AS1* and its experimental validation in hepatocellular carcinoma (HCC). We integrated datasets from The Cancer Genome Atlas (TCGA), the Genotype‐Tissue Expression Project (GTEx), the cancer immunology data engine (CIDE), and the cBioPortal for cancer genomics (cBioPortal) to systematically assess its expression pattern, genomic alterations, clinical significance, and immunological associations. Our analyses revealed that *UPK1A-AS1* is significantly upregulated in multiple tumor types, with copy‐number amplification as the predominant genomic alteration driving its overexpression. Elevated *UPK1A-AS1* expression was correlated with advanced disease stage, poor differentiation, immune exclusion, and unfavorable prognosis, supporting its potential as a cancer type‐dependent biomarker. In parallel, functional studies demonstrated that hypoxia transcriptionally induces *UPK1A-AS1* in HCC, where it promotes sorafenib resistance by suppressing apoptosis. Silencing *UPK1A-AS1* restored apoptotic and enhanced sorafenib efficacy both in vitro and in vivo. Collectively, our findings suggest that *UPK1A-AS1* is a hypoxia‐inducible oncogenic lncRNA that plays dual roles in cancer, with cancer type‐dependent associations with progression and immune modulation across malignancies and mechanistically mediating hypoxia‐associated drug resistance in HCC.

## 1. Introduction

Cancer remains one of the leading causes of morbidity and mortality worldwide and imposes a substantial public health burden. For numerous cancer types, particularly at the advanced stages, survival outcomes are still unsatisfactory. Global cancer statistics indicate that the incidence and mortality of many malignancies remain high despite advances in prevention, early detection, and systemic therapies [1]. These challenges highlight the urgent need to clarify the molecular mechanisms underlying tumor initiation and progression and to identify reliable biomarkers and therapeutic targets to improve cancer management.

Long noncoding RNAs (lncRNAs), defined as transcripts longer than 200 nucleotides without significant protein‐coding potential, have emerged as key regulators of gene expression and cellular behavior [2, 3]. These molecules participate in chromatin remodeling, transcriptional regulation, RNA processing, and posttranscriptional control through mechanisms such as scaffolding, guiding, or decoying regulatory proteins [2]. Dysregulated lncRNAs have been shown to modulate multiple hallmarks of cancer, influencing cell proliferation, apoptosis, migration, genomic stability, and metabolic reprogramming [3–5]. Moreover, some lncRNAs can encode micropeptides that actively contribute to oncogenic signaling, further underscoring the functional diversity of this transcript class [4]. Given their cancer‐ and tissue‐specific expression patterns, lncRNAs have gained increasing attention as promising diagnostic and prognostic biomarkers, as well as potential therapeutic targets [5].

Among these cancer‐associated lncRNAs, UPK1A antisense RNA 1 (*UPK1A-AS1*) has recently emerged as a functionally versatile transcript implicated in malignancies. Our previous work demonstrated that *UPK1A-AS1* is significantly upregulated in hepatocellular carcinoma (HCC) and that its high expression correlates with a poor patient prognosis. Mechanistic studies revealed that *UPK1A-AS1* promotes cancer cell proliferation by interacting with *EZH2* and facilitating histone‐mediated repression of tumor‐suppressive genes [6]. Additionally, in pancreatic cancer, *UPK1A-AS1* is induced by cancer‐associated fibroblast‐derived IL‐8 through NF‐κB signaling and confers platinum resistance by enhancing DNA double‐strand break repair via the Ku70/Ku80‐dependent nonhomologous end‐joining pathway [7]. In contrast, in esophageal squamous cell carcinoma (ESCC), *UPK1A-AS1* is downregulated and functions as a tumor suppressor, inhibiting cell proliferation, migration, and invasion by sponging *miR-1248* [8]. Similar context‐dependent functional patterns have been reported for other cancer‐associated lncRNAs. *SNHG10* has been implicated in the regulation of proliferation, apoptosis, drug resistance, metabolic reprogramming, and immune evasion, but its expression patterns and biological effects vary substantially across tumor types [9]. Likewise, *SOCS2-AS1* functions as a tumor suppressor in HCC, colorectal cancer, and endometrial cancer while exhibiting oncogenic properties in papillary thyroid cancer, glioma (GBMLGG), and prostate cancer [10]. Collectively, these findings suggest that the biological effects of cancer‐associated lncRNAs are strongly shaped by tissue‐specific molecular and microenvironmental contexts. Accordingly, *UPK1A-AS1* may act as an oncogene in some tumor types while serving as a tumor suppressor in others. This functional heterogeneity highlights the necessity of a comprehensive pan‐cancer investigation to clarify the biological roles and regulatory mechanisms of *UPK1A-AS1* across malignancies.

Considering its divergent and tumor type‐specific functions, the overall biological and clinical relevance of *UPK1A-AS1* across human cancers has yet to be systematically defined. Integrative studies combining multidatabase transcriptomic profiling with prognostic and immune microenvironment analyses offer a valuable framework for investigating the roles of cancer‐associated molecules [11]. Adopting this strategy, we performed a comprehensive pan‐cancer analysis to delineate the expression patterns of *UPK1A-AS1* in normal tissues and multiple tumor types and to clarify its associations with clinicopathological features, patient prognosis, and genomic alterations. We further investigated the relationship between *UPK1A-AS1* expression and the tumor immune microenvironment, including immune and stromal cell infiltration, immunogenic features, and responses to immune checkpoint blockade across cancers. In addition, we combined in vitro and in vivo HCC models to investigate the functional role of *UPK1A-AS1* in tumor progression and hypoxia‐associated sorafenib resistance. Together, these integrated bioinformatics and experimental analyses provide a systematic view of the biological and clinical importance of *UPK1A-AS1* and support its potential as a prognostic biomarker and therapeutic target in cancer.

## 2. Materials and Methods

### 2.1. *UPK1A-AS1* Gene Expression Analysis

The expression pattern of *UPK1A-AS1* across normal and tumor tissues was analyzed via RNA‐sequencing data from the Genotype‐Tissue Expression Project (GTEx) [12] and The Cancer Genome Atlas (TCGA) databases, which were obtained from the UCSC Xena platform (https://xenabrowser.net/datapages). Integrated pan‐cancer analyses combining TCGA and GTEx datasets were performed via Sangerbox (http://sangerbox.com/) to evaluate the expression landscape of *UPK1A-AS1* across diverse cancer types. Furthermore, the cancer immunology data engine (CIDE) database (https://cide.ccr.cancer.gov) was utilized to explore the cell type‐specific expression profile of *UPK1A-AS1* within the tumor microenvironment via single‐cell transcriptomic data.

### 2.2. Genetic Alteration Analysis

Genetic alterations in *UPK1A-AS1* across various cancer types were analyzed via the cBioPortal for cancer genomics (cBioPortal) (https://www.cbioportal.org), which integrates multidimensional genomic data from TCGA Pan‐Cancer Atlas studies. The frequency and distribution of alteration types, including mutations, copy‐number variations (CNVs), and amplifications, were summarized across TCGA tumor cohorts to provide an overview of *UPK1A-AS1* alteration patterns. Additionally, the somatic mutation profiles of the high‐ and low‐*UPK1A-AS1* expression groups (top 50% vs. bottom 50%) were compared via Sangerbox, which stratifies TCGA samples by gene expression and generates group‐specific mutation landscapes visualized as waterfall plots.

### 2.3. Functional Enrichment Analysis

To investigate the biological functions and signaling pathways associated with *UPK1A-AS1*, enrichment analyses were performed on both genomic and transcriptomic data. Genes showing significant co‐mutation with *UPK1A-AS1* across multiple cancer types were retrieved from the cBioPortal, where co‐mutation significance was assessed using one‐sided Fisher’s exact tests with Benjamini–Hochberg FDR correction, and the top 500 genes with the strongest correlation (*p*‐value < 0.05, ranked by the log2 ratio) were selected for subsequent pathway enrichment analysis (Table S1). Differentially expressed genes (DEGs) between the high‐ and low‐*UPK1A-AS1* expression groups in TCGA cohort were identified via the edgeR [13] package with the Benjamini–Hochberg method applied for multiple testing correction (adjusted *p*  < 0.05 and |log_2_FC| > 1, Table S2). These gene sets were subjected to Kyoto Encyclopedia of Genes and Genomes (KEGG) [14] and Gene Ontology (GO) [15] enrichment analyses to determine the functional categories and molecular pathways potentially regulated by *UPK1A-AS1*. All analyses and visualizations were conducted via R software (Version 4.3.3) [16] with the clusterProfiler [17], org.Hs.eg.db [18], GOplot [19], and ggplot2 [20] packages under default parameters.

### 2.4. Immune Infiltration and Immune‐Related Gene Analysis

Comprehensive immune infiltration profiling was performed across multiple cancer types via Sangerbox, which integrates transcriptomic and immune‐related data derived from TCGA cohorts. The tumor immune estimation resource (TIMER) [21] embedded within Sangerbox was used to quantify the associations between *UPK1A-AS1* expression and immune infiltration levels and to evaluate correlations with immune checkpoint‐related genes, chemokines, and chemokine receptors. To further estimate the relative abundance of immune and stromal components within tumor tissues, Estimation of STromal and Immune cells in MAlignant Tumours using Expression data (ESTIMATE) [22] was implemented in R. Radar plots summarizing the immune infiltration characteristics were generated via the fmsb [23] package in R to provide integrated visualization of the immune landscape associated with *UPK1A-AS1* expression.

### 2.5. Survival and Prognostic Analysis

The prognostic value of *UPK1A-AS1* was evaluated across cancer types on the basis of patient survival data. In TCGA cohort, patients were stratified into high‐ and low‐expression groups according to the top 50% and bottom 50% of *UPK1A-AS1* expression levels, and overall survival (OS), progression‐free survival (PFS), disease‐free survival (DFS), and disease‐specific survival (DSS) were analyzed via Sangerbox. Additional survival data derived from the CIDE database were used to examine the prognostic relevance of *UPK1A-AS1* expression in immunotherapy‐treated cohorts. The predictive performance related to the immune checkpoint blockade response was further validated via the ROC Plotter (https://rocplot.com/immune).

### 2.6. Cell Culture

The HCC cell lines Huh7 (RRID: CVCL_0336) and MHCC‐97H (RRID: CVCL_4972) were obtained from the Cell Bank of Type Culture Collection (CBTCC, Chinese Academy of Sciences, Shanghai, China). These two cell lines were selected because they represent distinct metastatic potentials (Huh7 is less invasive, while MHCC‐97H is highly invasive) and are widely used models for HCC research. The use of these authenticated cell lines with valid RRIDs ensures the reproducibility and reliability of our findings. Cells were cultured in Dulbecco’s Modified Eagle’s Medium (DMEM, Gibco) supplemented with 10% fetal bovine serum (FBS, Gibco) and maintained at 37°C in a humidified incubator containing 5% CO_2_. For hypoxia treatment, cells were incubated for 24 h under a gas mixture containing 1% O_2_, 5% CO_2_, and 94% N_2_. Cells maintained under normoxic conditions were used as controls.

### 2.7. RNA Interference

The siRNAs specifically targeting *UPK1A-AS1* and the corresponding negative controls were obtained from RiboBio. Briefly, 50 nM of each siRNA was transfected into cells via Lipofectamine RNAiMAX (Invitrogen) according to the manufacturer’s protocol. The sequences of the siRNAs used are as follows: si‐AS1‐1, 5′‐GGAGATGCTTGGTCCATCT‐3′; si‐AS1‐2, 5′‐CCTTGACAGTGCTGTTATT‐3′.

### 2.8. Lentiviral Construction and Transduction

The hU6‐shUPK1A‐AS1‐CMV lentiviral vectors used to knock down *UPK1A-AS1* expression were designed and constructed by Genechem Company Ltd. To generate clones with stable *UPK1A-AS1* knockdown, MHCC‐97H cells were infected at a multiplicity of infection of 30 with hU6‐shUPK1A‐AS1‐CMV lentiviral vectors or control vectors. The stable *UPK1A-AS1*‐downregulated clones were selected after 2 weeks of treatment with puromycin, and the expression of *UPK1A-AS1* was determined.

### 2.9. RNA Extraction and qRT‐PCR

Total RNA was isolated via the TRIzol reagent (Invitrogen) following the manufacturer’s recommendations. qRT‐PCR was performed using a SYBR Green PCR kit (Takara Biotechnology). β‐Actin genes were used as internal controls. The specificity of the amplification products was confirmed by the melting curve analysis. Triplicate samples were analyzed in three independent experiments. The sequences of primers used were as follows: *UPK1A-AS1*, F: 5′‐AGAGCGGTGGGTTAGGAA‐3′, and R: 5′‐GGGCAGATGGACCAAGCA‐3′. *β-Actin*, F: 5′‐TCAAGATCATTGCTCCTCCTGA‐3′, R: 5′‐CTCGTCATACTCCTGCTTGCTG‐3′. *U6*, F: 5′‐CTCGCTTCGGCAGCACA‐3′, R: 5′‐AACGCTTCACGAATTTGCGT‐3′.

### 2.10. Immunohistochemistry (IHC) Assay

Paraffin‐embedded tumor tissue sections (5 μm thick) were labeled with primary antibodies, and immunoreactivity was detected with an envision system (Dako, Carpinteria). Briefly, formalin‐fixed, paraffin‐embedded tissue sections were deparaffinized, rehydrated, and subjected to heat‐induced epitope retrieval in citrate buffer (pH 6.0) via a pressure cooker. After blocking endogenous peroxidase activity and nonspecific binding, the sections were incubated with primary antibody (Mouse Monoclonal anti‐Ki‐67, Abcam, Cat Number ab279653; Cleaved Caspase‐3 (Asp175) Antibody, CST, Cat Number 9661; Rabbit Monoclonal anti‐HIF‐1α, CST, Cat Number 36169) overnight at 4°C. Following washes, detection was carried out via a horseradish peroxidase (HRP)‐labeled polymer detection system and 3,3′‐diaminobenzidine (DAB) chromogen. The slides were mounted per mount after dehydration.

### 2.11. Flow Cytometry Assay

Apoptotic cells were evaluated with an annexin‐V fluorescein isothiocyanate and propidium iodide apoptosis detection kit (Invitrogen) following the manufacturer’s protocol. The stained cells were then analyzed with a FACScan flow cytometer.

### 2.12. Animal Study

All procedures for the animal study were approved by the Animal Use and Care Committee of Nanfang Hospital, Southern Medical University (Guangzhou, China). Four‐week‐old male nude mice were subcutaneously injected with 1 × 10^7^ MHCC‐97H cells and subjected to the indicated treatments. Mice were randomly assigned to each group (*n* = 6 per group) using a random number table on day 13 after MHCC‐97H cell injection when the tumor volume reached 100–150 mm^3^. Drug administration was initiated immediately after randomization, and mice received daily oral sorafenib (30 mg/kg/day) or equal volume PBS until sacrifice. The tumor size was measured daily via an electronic caliper, and the tumor volume was calculated as (length × width^2^) / 2. The tumor‐bearing mice were sacrificed for further analysis 19 days after the injection of MHCC‐97H cells. All tumor measurements and analyses were performed with investigators blinded to the group allocation.

### 2.13. Statistical Methods

Statistical analyses were performed using GraphPad Prism 8.0, R 4.3.3, and public online platforms. For two‐group comparisons, Student’s *t*‐test or the Wilcoxon rank‐sum test was used as appropriate. *UPK1A-AS1* expression levels in paired HCC and adjacent nontumor tissues were compared using a paired Student’s *t*‐test. Comparisons among multiple groups were performed using one‐way ANOVA, followed by Tukey’s post‐hoc test. Details of correlation analyses are provided in the corresponding figure legends. Survival curves were generated by the Kaplan–Meier method and compared by the log‐rank test; forest plots were based on univariate Cox regression with Wald tests. Mutation frequency differences were evaluated by Fisher’s exact test. All statistical tests were two‐sided, and a *p*‐value of less than 0.05 was considered statistically significant.

## 3. Results

### 3.1. Tissue‐ and Cell Type‐Specific Expression Profiles of *UPK1A-AS1* in Human Cancers

Given that gene expression patterns can profoundly influence tumor behavior and cellular function, we next investigated the expression characteristics of *UPK1A-AS1* across normal tissues and various cancer types. Analysis of the GTEx data revealed that *UPK1A-AS1* exhibited variable expression levels across a wide range of normal human tissues. Relatively high expression was observed in the skin, vagina, testis, and bladder (Figure 1A). We therefore used TCGA database to examine the expression landscape of *UPK1A-AS1* across various cancer types. The results indicated that *UPK1A-AS1* was expressed at higher levels in multiple malignancies, including esophageal carcinoma (ESCA), lung adenocarcinoma (LUAD), ovarian cancer (OV), pancreatic ductal adenocarcinoma (PAAD), breast cancer (BRCA), lung squamous cell carcinoma (LUSC), sarcoma (SARC), uterine corpus endometrial carcinoma (UCEC), liver HCC (LIHC), acute myeloid leukemia (LAML), mesothelioma (MESO), cholangiocarcinoma (CHOL), uterine carcinosarcoma (UCS), uveal melanoma (UVM), bladder carcinoma (BLCA), testicular germ cell tumor (TGCT), and skin cutaneous melanoma (SKCM) (Figure 1B). Further integrative analysis via Sangerbox, which is based on combined TCGA and GTEx datasets, revealed differential expression patterns of *UPK1A-AS1* across various cancer types. In the paired tumor and adjacent normal samples from TCGA cohort (Figure 1C), *UPK1A-AS1* expression was significantly downregulated in glioblastoma multiforme (GBM), GBMLGG, lower‐grade GBMLGG (LGG), BRCA, kidney renal papillary cell carcinoma (KIRP), pan‐kidney cohort (KIPAN), prostate adenocarcinoma (PRAD), kidney renal clear cell carcinoma (KIRC), and kidney chromophobe (KICH). In contrast, it was upregulated in colon and rectal adenocarcinoma (COADREAD), LUSC, LIHC, and CHOL. In the unpaired analysis combining TCGA and GTEx datasets (Figure 1D), *UPK1A-AS1* expression was significantly downregulated in GBM, GBMLGG, LGG, BRCA, LUAD, ESCA, stomach and ESCA (STES), KIRP, KIPAN, colon adenocarcinoma (COAD), PRAD, KIRC, Wilms tumor (WT), thyroid carcinoma (THCA), TGCT, and KICH. Conversely, it was upregulated in stomach adenocarcinoma (STAD), LIHC, SKCM, OV, PAAD, UCS, LAML, adrenocortical carcinoma (ACC), and CHOL. Collectively, these findings suggest that this lncRNA may serve as a potential regulatory molecule involved in tumorigenesis and cancer progression across diverse malignancies.

Figure 1Expression pattern of *UPK1A-AS1* across normal tissues and pan‐cancer samples. (A) Expression profile of *UPK1A-AS1* across 31 normal human tissues from the GTEx database. (B) Differential expression of *UPK1A-AS1* across multiple cancer types on the basis of TCGA datasets. (C,D) Comparative analysis of *UPK1A-AS1* expression in tumor and normal tissues across multiple cancer types using the unpaired Wilcoxon rank‐sum (Mann–Whitney U) test. (C) Comparison of *UPK1A-AS1* expression between paired tumor and adjacent normal tissues from TCGA cohort. (D) Integrated TCGA–GTEx analysis showing *UPK1A-AS1* expression differences between tumor and normal samples.  ^∗^ *p* < 0.05,  ^∗∗^ *p* < 0.01,  ^∗∗∗^ *p* < 0.001,  ^∗∗∗∗^ *p* < 0.0001, and  ^−^ *p* ≥ 0.05. (E) Single‐cell transcriptomic analysis from the CIDE database indicating the cell type‐specific distribution of *UPK1A-AS1* expression within the tumor microenvironment.

**Figure:**
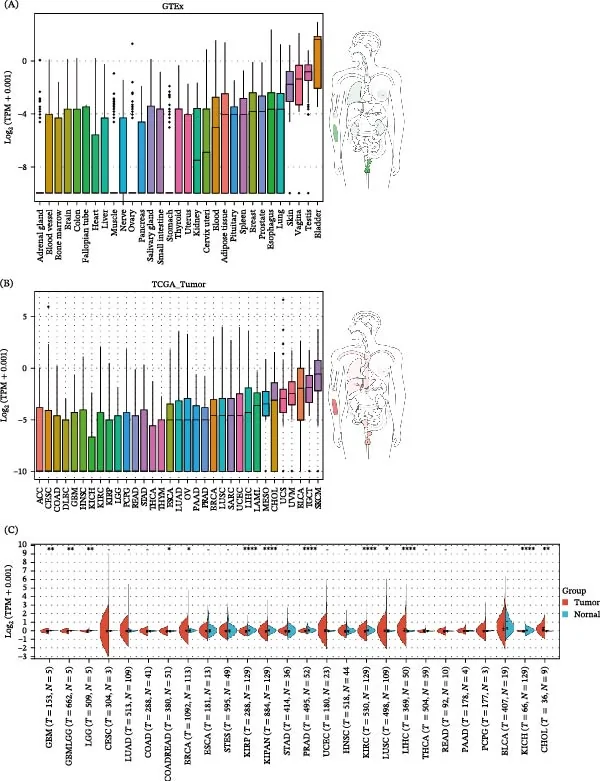

**Figure:**
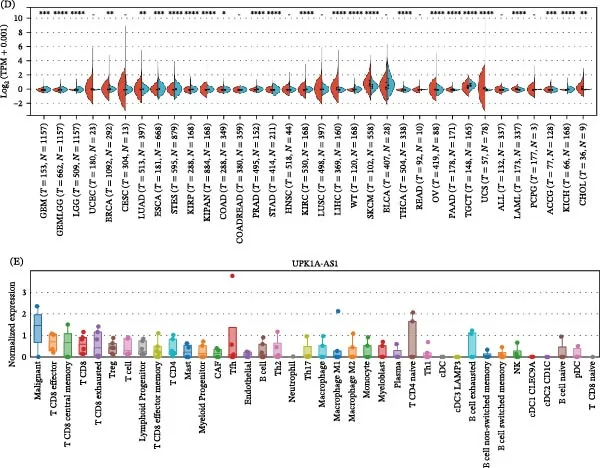

To further investigate the expression pattern of *UPK1A-AS1* among different cell types within the tumor microenvironment, we analyzed its distribution via the CIDE database. The results indicated that *UPK1A-AS1* was predominantly expressed in tumor cells. In addition, higher expression levels were observed across several T‐cell subpopulations, including effector CD8^+^ T cells, central memory CD8^+^ T cells, exhausted CD8^+^ T cells, and regulatory T cells, whereas its expression in naïve T cells was very low or barely detectable on the basis of transcriptomic data (Figure 1E and Figure S1A). The enrichment of *UPK1A-AS1* in tumor cells suggests a potential tumor‐promoting role, while its specific expression pattern among multiple T‐cell subsets implies that this lncRNA may also contribute to the regulation of the tumor immune microenvironment and T‐cell functional states.

### 3.2. Clinical Correlations and Prognostic Value of *UPK1A-AS1* Across Cancers

**Figure 2:**
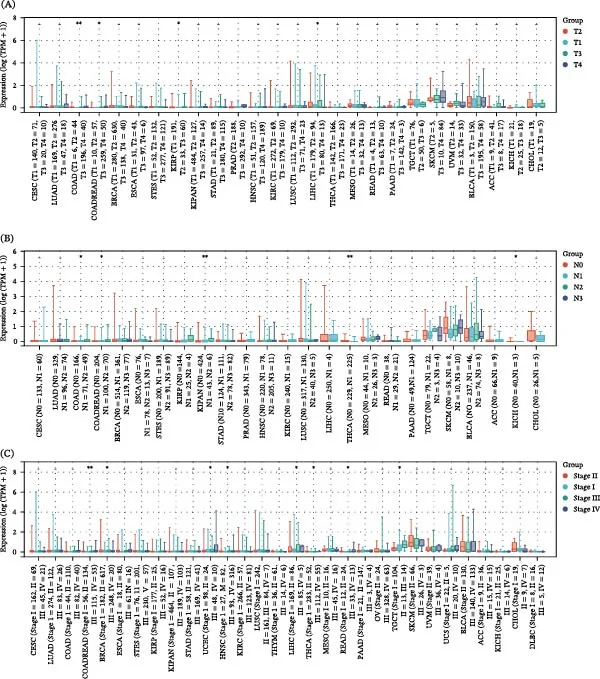
Correlations between *UPK1A-AS1* expression and clinicopathological characteristics across cancers. (A) Boxplots showing the expression levels of *UPK1A-AS1* in tumors with different invasion depths (*T* stage) across TCGA cohorts. (B) Comparison of *UPK1A-AS1* expression among groups with distinct lymph node metastasis statuses (*N* stage). (C) Expression pattern of *UPK1A-AS1* across different overall clinical stages (Stages I–IV) in various cancer types. All comparisons in (A–C) were performed using the unpaired Student’s t‐test.  ^∗^ *p* < 0.05,  ^∗∗^ *p* < 0.01, and  ^−^ *p* ≥ 0.05.

To explore the potential impact of *UPK1A-AS1* on the prognosis of multiple cancer types, we performed a systematic analysis via Sangerbox. The results revealed that the expression level of *UPK1A-AS1* was significantly associated with various clinicopathological features across multiple malignancies, suggesting its potential involvement in tumor initiation and progression. With respect to tumor invasion depth (*T* stage), *UPK1A-AS1* exhibited differential expression in COAD, COADREAD, KIRP, and LIHC (Figure 2A), indicating a possible link between this gene and local invasive behavior. In terms of lymph node metastasis (*N* stage), differential expression of *UPK1A-AS1* was observed in COAD, COADREAD, KIPAN, THCA, and KICH (Figure 2B), further implying that *UPK1A-AS1* plays a role in regulating local metastatic processes. Notably, for distant metastasis (*M* stage), significant differences in expression were detected only in COADREAD (Figure S2A), suggesting a specific role in the distant metastasis of colorectal cancer. From the perspective of overall tumor stage, *UPK1A-AS1* expression was correlated with disease progression in COAD, COADREAD, UCEC, head and neck squamous cell carcinoma (HNSC), LIHC, THCA, rectum adenocarcinoma (READ), and TGCT (Figure 2C). In addition, a significant association with the tumor differentiation grade was observed only in LIHC (Figure S2B), indicating that *UPK1A-AS1* may serve as a potential indicator for malignancy assessment in HCC. Moreover, its expression differed according to sex in LGG, BRCA, SARC, KIRP, LIHC, and ACC (Figure S2C).

We next assessed the prognostic value of *UPK1A-AS1* across cancers via Sangerbox survival analyses. Elevated *UPK1A-AS1* expression was significantly associated with poorer OS in multiple cancer types, including KIPAN (HR = 1.12, *p* = 5.9e − 9), KIRC (HR = 1.10, *p* = 3.3e − 5), ACC (HR = 1.21, *p* = 4.4e − 4), KIRP (HR = 1.13, *p* = 3.6e − 3), and MESO (HR = 1.14, *p* = 8.5e − 3) (Figure 3A). Similarly, high *UPK1A-AS1* expression correlated with shorter PFS in KIPAN (HR = 1.13, *p* = 1.1e − 9), KIRC (HR = 1.12, *p* = 1.1e − 6), ACC (HR = 1.23, *p* = 2.8e − 6), STAD (HR = 1.09, *p* = 8.7e − 4), STES (HR = 1.06, *p* = 3.2e − 3), KIRP (HR = 1.11, *p* = 6.7e − 3), and pheochromocytoma and paraganglioma (PCPG, HR = 1.16, *p* = 0.03) patients (Figure 3C). Kaplan–Meier curves revealed that elevated *UPK1A-AS1* expression was significantly associated with reduced OS in ACC, KIPAN, KIRC, KIRP, MESO, STAD, and LIHC patients (Figure 3B). Similarly, high *UPK1A-AS1* expression correlated with shorter PFS in ACC, KIPAN, KIRP, LIHC, LUSC, LGG, STAD, and STES (Figure 3D). These results consistently indicate that *UPK1A-AS1* functions as a potential adverse prognostic factor across diverse human cancers.

Figure 3Prognostic significance of *UPK1A-AS1* expression across multiple cancer types. For forest plots (A, C), hazard ratios and *p*‐values were derived from univariate Cox proportional hazards models with Wald tests; for Kaplan–Meier curves (B, D), group comparisons were performed using log‐rank tests. (A) Forest plot showing the association between *UPK1A-AS1* expression and overall survival (OS) across various cancers. (B) Kaplan–Meier survival curves illustrating the relationship between *UPK1A-AS1* expression and OS in representative tumor types. (C) Forest plot showing the association between *UPK1A-AS1* expression and progression‐free survival (PFS) across cancers. (D) Kaplan–Meier survival curves showing the correlation between *UPK1A-AS1* expression and PFS in selected cohorts.

**Figure:**
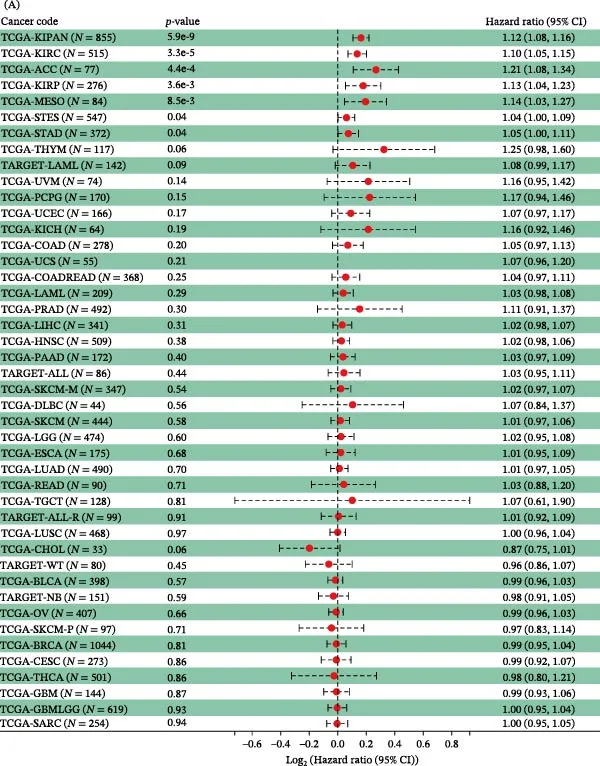

**Figure:**
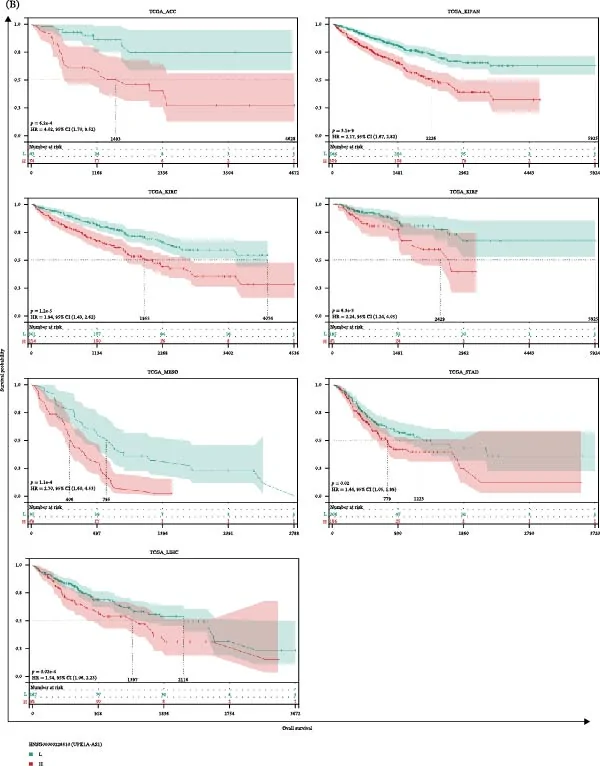

**Figure:**
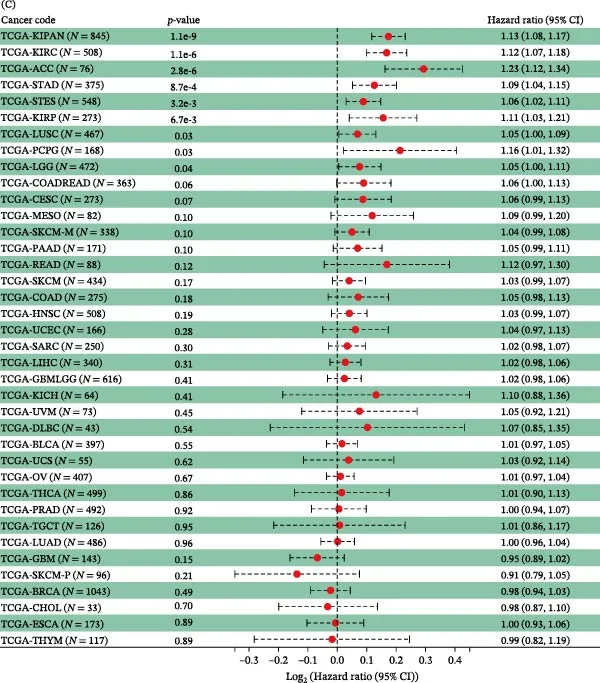

**Figure:**
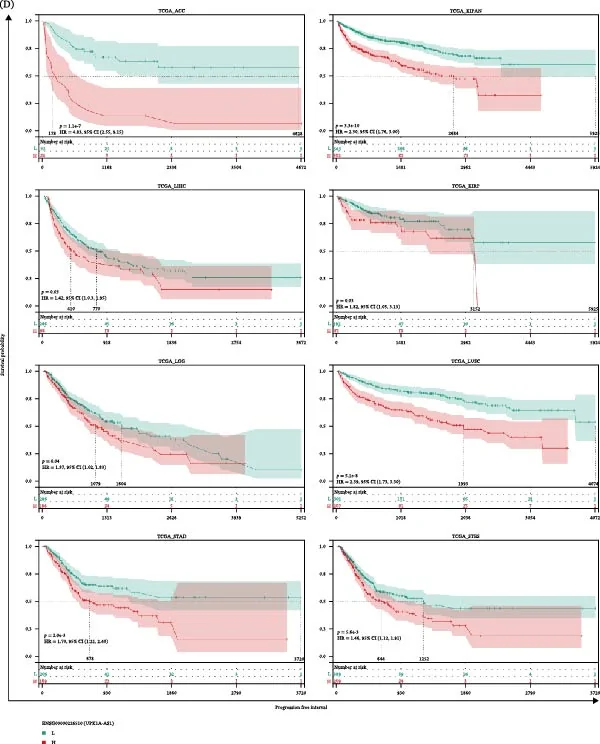

### 3.3. Genomic Alterations and Functional Implications of *UPK1A-AS1* Across Human Cancers

In cancer genomics, somatic mutations are among the key drivers of tumorigenesis. These mutations include point mutations, insertions or deletions, and large‐scale structural variations such as CNVs, which can lead to oncogene activation or tumor suppressor gene inactivation [24]. Using the cBioPortal, we examined the mutation frequency of *UPK1A-AS1* across various cancers. Copy‐number amplification was the predominant alteration type and was observed at relatively high frequencies in multiple tumor types, with the highest amplification rate detected in UCS (19.3%). Other cancers with relatively high amplification frequencies included OV (8.22%), LUSC (5.34%), esophageal adenocarcinoma (3.85%), SARC (4.31%), UCEC (3.21%), cervical squamous cell carcinoma (3.37%), PAAD (3.26%), LUAD (3.00%), and BLCA (2.43%) (Figure 4A).

Figure 4Genomic alterations and functional enrichment analysis of *UPK1A-AS1* across cancers. (A) Distribution of genomic alterations, including mutations, amplifications, and deletions, across multiple tumor types. (B) Kaplan–Meier survival analysis comparing overall survival (OS) and progression‐free survival (PFS) between the altered and unaltered groups, with statistical significance evaluated by the log‐rank test. (C) Mutation landscape plots showing representative alteration profiles in tumors with different *UPK1A-AS1* expression levels. Statistical significance of mutation frequency differences between groups was assessed by two‐sided Fisher’s exact test.  ^∗^ indicates statistical significance. (D) KEGG pathway enrichment analysis showing the major pathways associated with *UPK1A-AS1*‐related alterations. (E) Gene Ontology (GO) enrichment analysis summarizing the biological processes, cellular components, and molecular functions associated with UPK1A‐AS1.

**Figure:**
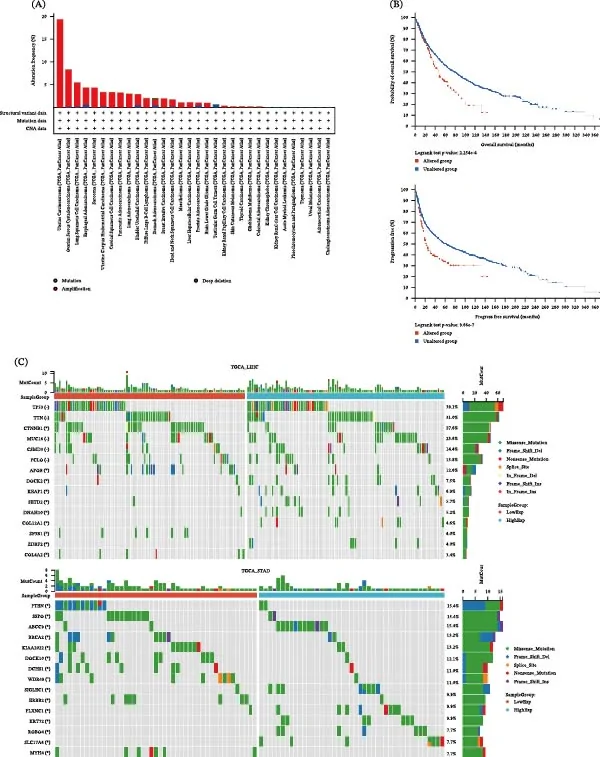

**Figure:**
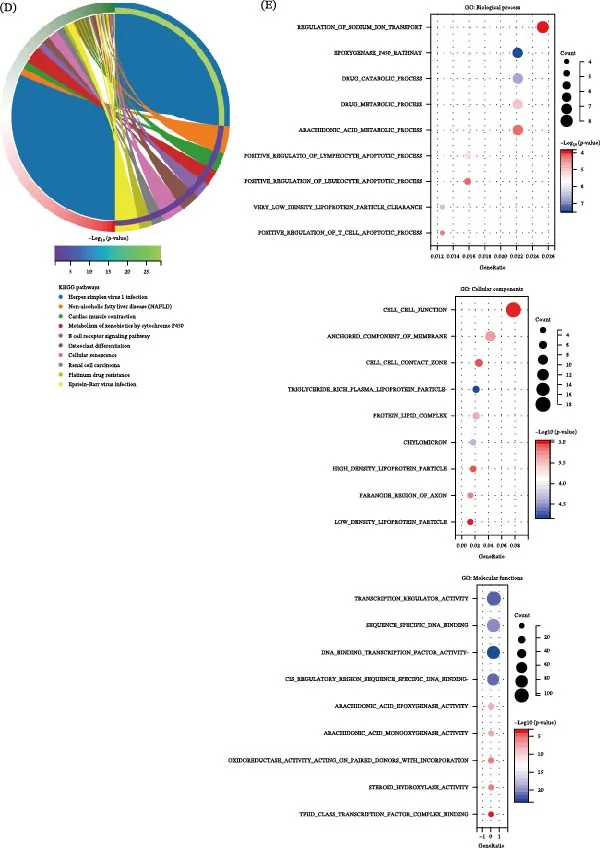

To further evaluate the clinical impact of these alterations, we compared OS and PFS between patients with and without *UPK1A-AS1* alterations. Kaplan–Meier survival analysis and log‐rank tests revealed that patients without alterations presented significantly better OS, PFS, DFS, and DSS than those with alterations did, indicating that *UPK1A-AS1* genomic alterations may be associated with unfavorable clinical outcomes (Figure 4B and Figure S3A,B).

We subsequently used Sangerbox to explore the relationship between *UPK1A-AS1* expression and specific genomic features in LIHC and STAD. High *UPK1A-AS1* expression was associated with increased frequencies of somatic mutations in *DOCK2* (7.5%), *KEAP1* (6.9%), *SETD2* (5.7%), *DNAH10* (5.2%), *COL12A1* (4.6%), and *ZDBF2* (4.0%) in LIHC and in *ABCC9* (15.4%), *SIGLEC1* (9.9%), *PLXNC1* (9.9%), *KRT72* (7.7%), *ROBO4* (7.7%), and *SLC17A6* (7.7%) in STAD (Figure 4C).

We next retrieved genes co‐mutated with *UPK1A-AS1* across pan‐cancer samples via the cBioPortal and performed KEGG pathway enrichment analysis. The results revealed several prominent pathways, including the metabolism of xenobiotics by cytochrome P450, the B‐cell receptor signaling pathway, renal cell carcinoma, platinum drug resistance, and Epstein–Barr virus infection (Figure 4D). Moreover, GO enrichment analysis further revealed significant enrichment in drug metabolism‐related processes and positive regulation of lymphocyte, leukocyte, and T‐cell apoptotic processes, as well as additional enrichment in transcriptional regulatory pathways, arachidonic acid metabolism, and lipoprotein‐associated metabolic processes (Figure 4E). Collectively, these results suggest that the expression and mutation of *UPK1A-AS1* may influence key biological processes, including transcriptional regulation, tumor immunity, chemoresistance, and metabolic reprogramming, thereby contributing to cancer initiation and progression. The genomic characteristics of *UPK1A-AS1* may thus hold potential predictive value in the diagnosis and therapy of cancer.

Collectively, the strong associations between *UPK1A-AS1* expression, genomic aberrations, and unfavorable patient prognosis indicate that this lncRNA may act as an oncogenic regulator involved in tumor progression and could represent a novel prognostic biomarker and therapeutic candidate across diverse cancer types.

### 3.4. *UPK1A-AS1* is Linked to Immune Infiltration and Immune Signaling Heterogeneity Across Cancers

To investigate the potential impact of *UPK1A-AS1* on the tumor immune microenvironment, we conducted a pan‐cancer analysis using ESTIMATE, which generates immune, stromal, and ESTIMATE scores to evaluate the level of immune and stromal cell infiltration in tumor tissues. *UPK1A-AS1* expression was negatively correlated with one or more of these scores in multiple cancer types, including HNSC, BRCA, BLCA, ACC, UCS, UCEC, THCA, TGCT, SKCM, SARC, and LUSC (Figure 5A). Notably, BRCA, BLCA, ACC, and UCEC were negatively correlated with all three scores, suggesting that *UPK1A-AS1* may be associated with a “cold” tumor immune microenvironment. In contrast, PRAD exhibited positive correlations, suggesting a potential association between *UPK1A-AS1* and enhanced immune infiltration in this cancer type.

Figure 5Correlation between *UPK1A-AS1* expression and the tumor immune microenvironment across cancers. (A) Radar plots showing the correlations of *UPK1A-AS1* expression with the ESTIMATE, immune, and stromal scores in different cancers. The cancer types with negative correlations are highlighted in green, and those with positive correlations are highlighted in red. Correlation analyses were performed using Spearman’s rank‐correlation test. (B–C) Heatmaps depicting the relationship between *UPK1A-AS1* expression and infiltration levels of major immune‐cell subsets (B), as well as immune‐related genes including chemokines and their receptors (C), with all correlation coefficients and *p*‐values derived from Spearman’s rank‐correlation test.  ^∗^ *p* < 0.05,  ^∗∗^ *p* < 0.01, and  ^∗∗∗^ *p* < 0.001. (D) Gene Ontology (GO) enrichment analysis summarizing immune‐related biological processes associated with differential *UPK1A-AS1* expression.

**Figure:**
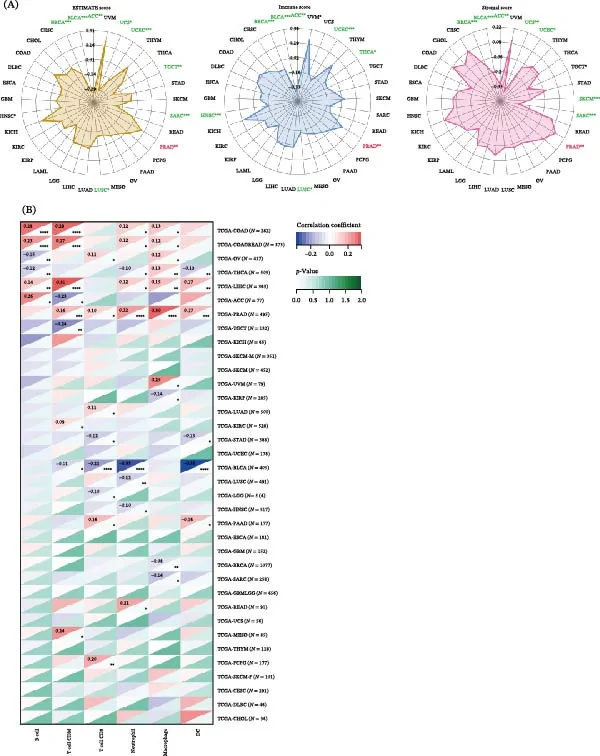

**Figure:**
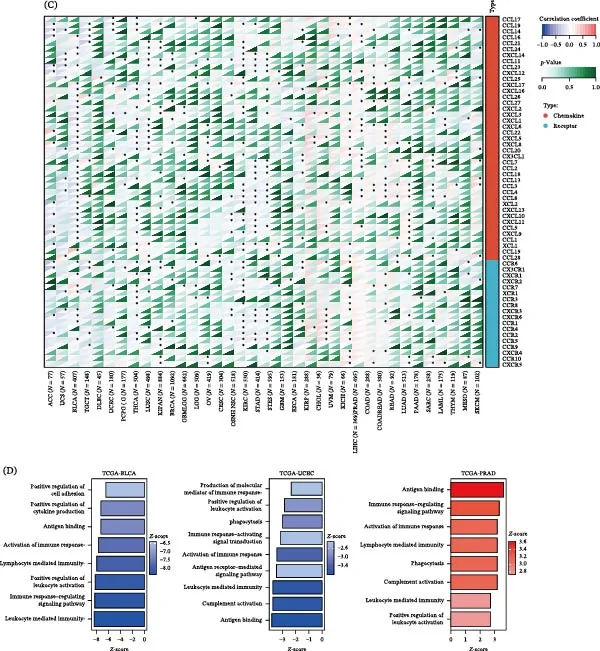

We further examined the association between *UPK1A-AS1* expression and immune‐cell infiltration levels via the TIMER algorithm implemented in Sangerbox. Consistently, *UPK1A-AS1* expression was positively correlated with the infiltration of multiple immune‐cell types in COAD, COADREAD, LIHC, and PRAD, whereas a negative correlation was observed in BLCA (Figure 5B). Interestingly, the correlation between *UPK1A-AS1* and CD4^+^ T‐cell infiltration was relatively high in COAD (*r* = 0.29), COADREAD (*r* = 0.27), and LIHC (*r* = 0.31), suggesting a potential association of *UPK1A-AS1* with CD4^+^ T‐cell infiltration within the tumor microenvironment. Collectively, these findings suggest that the *UPK1A-AS1* expression is closely associated with immune‐cell infiltration across multiple tumor types.

To further explore whether *UPK1A-AS1* might influence the tumor immune microenvironment by regulating chemokine signaling networks, we analyzed its correlation with various chemokines and their receptors across cancers. The results revealed widespread and significant associations between *UPK1A-AS1* and multiple C─C‐ and C─X─C‐motif chemokines and receptors. Negative correlations were predominant in ACC, BLCA, THCA, LUSC, KIRC, STAD, and STES, whereas positive correlations were observed in UVM, LIHC, PRAD, COAD, COADREAD, and LUAD (Figure 5C). These results suggest that *UPK1A-AS1* expression is associated with chemokine signaling in a cancer type‐dependent manner, implying a potential link to distinct tumor immune microenvironmental features.

To further elucidate the functional differences associated with *UPK1A-AS1*, GO and KEGG enrichment analyses were performed on the basis of the log_2_‐fold‐change values of DEGs between the high‐ and low‐expression groups. Consistent with the immune correlation results, the downregulated pathways in the BLCA and UCEC cohorts were related primarily to immune activation and leukocyte function, including positive regulation of cell adhesion, positive regulation of cytokine production, antigen binding, activation of the immune response, lymphocyte‐mediated immunity, positive regulation of leukocyte activation, the immune response‐regulating signaling pathway, and leukocyte‐mediated immunity. In contrast, in PRAD, high *UPK1A-AS1* expression was associated with the upregulation of multiple immune‐related processes, such as antigen binding, the immune response‐regulating signaling pathway, the activation of the immune response, lymphocyte‐mediated immunity, phagocytosis, complement activation, and leukocyte‐mediated immunity (Figure 5D). Collectively, these results indicate that *UPK1A-AS1* plays a multifaceted role in shaping the tumor immune landscape, reflecting its potential involvement in both immune regulation and tumor progression across diverse cancer types.

### 3.5. *UPK1A-AS1* Expression is Correlated With Immunogenic Features and Immunotherapy Response Across Cancers

Tumor mutational burden (TMB) and microsatellite instability (MSI) are key indicators of genomic instability and mutation frequency, both of which influence the formation of neoantigens (NEO) and the efficacy of immune checkpoint blockade [25]. To investigate the relationship between *UPK1A-AS1* and tumor immunogenicity, correlations between *UPK1A-AS1* expression and TMB, MSI, and NEO were analyzed across multiple cancer types via Sangerbox. In CHOL, *UPK1A-AS1* expression was negatively correlated, and high *UPK1A-AS1* expression may harbor lower mutational and NEO loads, thereby reducing immune recognition. A similar pattern of negative correlation with both TMB and MSI was observed in STAD and STES, further supporting the potential association between *UPK1A-AS1* upregulation and reduced genomic alteration. In contrast, *UPK1A-AS1* expression was positively correlated with both TMB and MSI in KICH (Figure 6A), suggesting a cancer type‐specific association with increased genomic instability and immunogenic features.

Figure 6Associations between *UPK1A-AS1* expression and tumor immunogenicity or immunotherapy response. (A) Correlations between *UPK1A-AS1* expression and tumor mutational burden (TMB), microsatellite instability (MSI), and neoantigen (NEO) load across cancer types, with correlation coefficients and *p*‐values derived from Pearson’s correlation test. (B) Correlation heatmap of *UPK1A-AS1* with immune checkpoint‐related genes, with correlation coefficients and *p*‐values derived from Spearman’s rank‐correlation test.  ^∗^ indicates statistical significance. (C,D) ROC analysis evaluating the predictive potential of *UPK1A-AS1* for the immune checkpoint blockade (anti‐PD‐L1) response. (E) Kaplan–Meier survival curves showing poorer outcomes in immune checkpoint inhibitor‐treated patients with high *UPK1A-AS1* expression across multiple cohorts, with survival differences evaluated by the log‐rank test.

**Figure:**
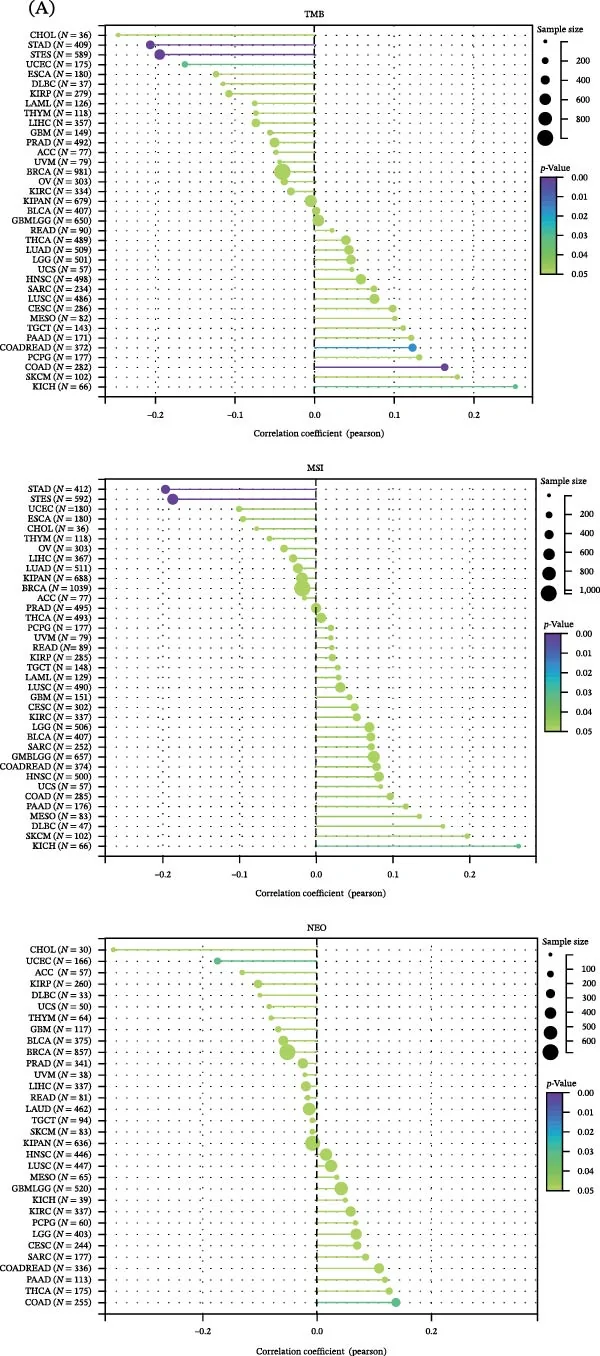

**Figure:**
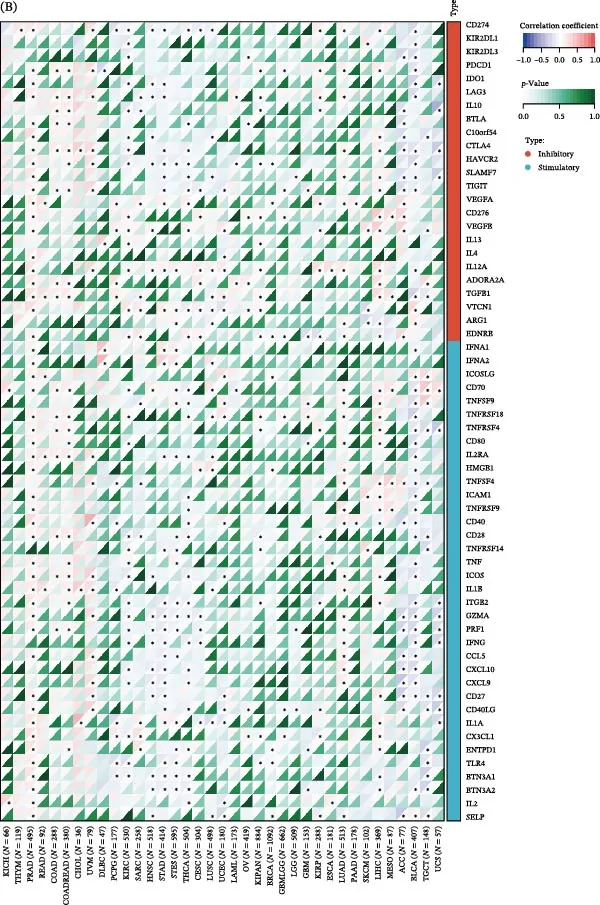

**Figure:**
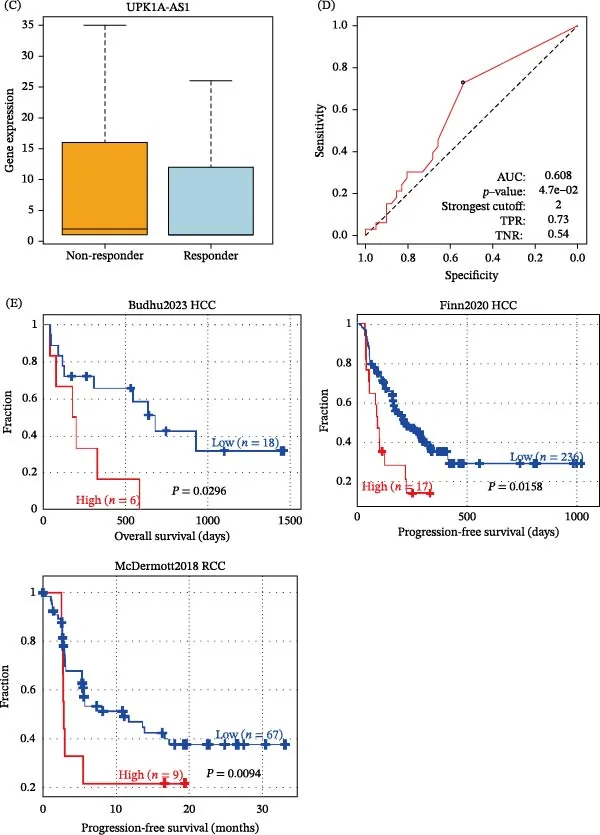

Analysis of immune checkpoint‐related genes revealed that *UPK1A-AS1* expression was broadly and positively correlated with the expression of multiple immune checkpoint molecules in PRAD, COAD, COADREAD, UVM, and LIHC (Figure 6B). These findings suggest that *UPK1A-AS1* expression is associated with distinct immune checkpoint‐related expression patterns and immunoregulatory features within the tumor microenvironment.

To further assess the clinical relevance of *UPK1A-AS1* in immune checkpoint blockade, we analyzed a cohort of patients with multiple cancers receiving anti‐PD‐L1 (*CD274*) therapy via the ROC Plotter. *UPK1A-AS1* expression was modestly higher in nonresponders than in responders (fold change = 1.7), although this difference did not reach statistical significance (*p* = 0.10) (Figure 6C). Receiver operating characteristic (ROC) analysis yielded an area under the curve (AUC) of 0.608 (*p* = 0.047), indicating a modest discriminatory potential for predicting immunotherapy response (Figure 6D). Despite the limited statistical significance, elevated *UPK1A-AS1* expression tended to be associated with reduced therapeutic benefit, a relationship that may differ across cancer types and requires further validation.

Survival analysis of the CIDE database further supported these findings. In multiple ICI‐treated cohorts, including Budhu2023 HCC, Finn2020 HCC, and McDermott2018 RCC, patients with high *UPK1A-AS1* expression consistently exhibited worse outcomes, characterized by shorter OS or PFS than those with low‐*UPK1A-AS1* expression (Figure 6E). Collectively, these results suggest that *UPK1A-AS1* upregulation may be linked to reduced tumor immunogenicity, diminished immunotherapy benefit, and unfavorable clinical outcomes.

### 3.6. UPK1A‐AS1 Promotes Hypoxia‐Induced Sorafenib Resistance by Suppressing Apoptosis

Hypoxia represents a defining feature of the tumor microenvironment and promotes malignant progression through the activation of hypoxia‐inducible factors (HIF‐1α and HIF‐2α) [26], which coordinate metabolic reprogramming, cellular plasticity, and immune evasion. Hypoxia‐regulated lncRNAs play crucial roles in this adaptive process, reshaping tumor metabolism and cellular programs to support survival, proliferation, and resistance under low‐oxygen conditions [27]. Interestingly, a previous study reported that *UPK1A-AS1* is upregulated under hypoxia in lung cancer cells [28]. To further delineate the hypoxia‐associated expression pattern of *UPK1A-AS1*, we examined multiple hypoxia‐treatment datasets available from the Single‐Cell Expression Atlas [29–31]. *UPK1A-AS1* expression was consistently and markedly upregulated across diverse human tumor cell models, including SW684, MDA‐MB‐468, 93T449, A2780, A2780cis, and SKUT1 (Figure 7A), indicating that *UPK1A-AS1* is transcriptionally induced under hypoxic stress. Importantly, HCC is characterized by a hypoxic tumor microenvironment, a critical feature resulting from uncontrolled tumor proliferation that outpaces oxygen supply and drives disease progression and therapeutic resistance [32]. Therefore, we next focused our investigation on HCC. Consistent with these observations, quantitative RT‐PCR analysis revealed a significant increase in *UPK1A-AS1* transcript levels in HCC cell lines Huh7 and MHCC‐97H following hypoxic exposure compared with normoxic conditions (Figure 7B), confirming the hypoxia‐responsive nature of *UPK1A-AS1*.

Figure 7Hypoxia induces *UPK1A-AS1* expression and promotes sorafenib resistance in HCC. (A) *UPK1A-AS1* upregulation under hypoxic conditions across multiple cancer cell models from the Single‐Cell Expression Atlas. (B) qRT‐PCR validation showing increased *UPK1A-AS1* expression in Huh7 and MHCC‐97H cells under hypoxia. (C) Knockdown efficiency of *UPK1A-AS1* siRNAs in MHCC‐97H cells. (D,E) Flow cytometry via Annexin V‐FITC/PI staining showing that hypoxia attenuates sorafenib‐induced apoptosis, whereas *UPK1A-AS1* knockdown restores apoptotic sensitivity. (F) qRT‐PCR analysis of UPK1A‐AS1 expression in 19 paired HCC tissues and matched adjacent nontumor tissues.  ^∗^ *p* < 0.05,  ^∗∗^ *p* < 0.01, and  ^∗∗∗^ *p* < 0.001.

**Figure:**
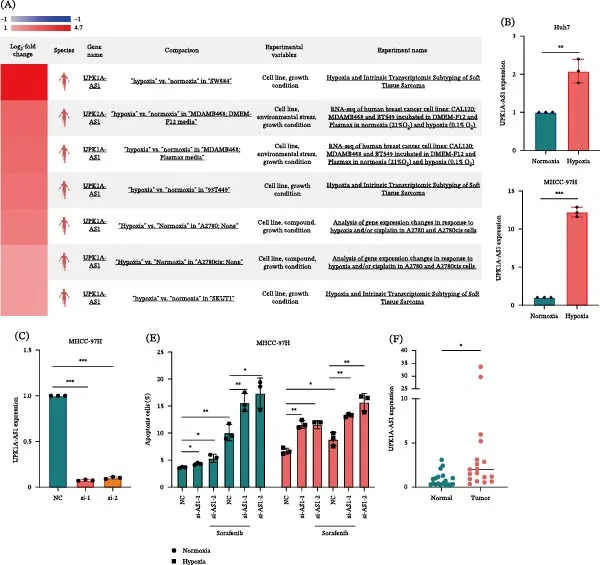

**Figure:**
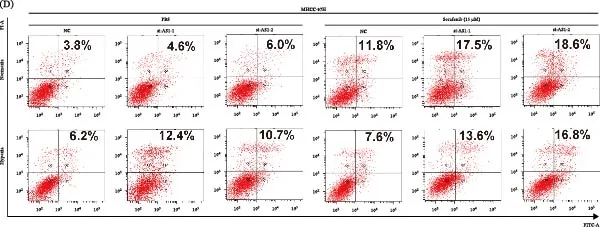

Sorafenib is a multikinase inhibitor that has long been used as a first‐line systemic therapy for advanced HCC [33]. However, despite its clinical utility, a significant proportion of patients exhibit primary or acquired resistance to sorafenib, which limits its therapeutic effectiveness and contributes to treatment failure in advanced HCC patients [34]. Because hypoxia reportedly diminishes sorafenib‐induced apoptosis via the activation of HIF‐dependent survival pathways [35], we next investigated whether *UPK1A-AS1* contributes to this adaptive resistance. MHCC‐97H cells were transfected with two independent siRNAs targeting *UPK1A-AS1* (si‐AS1‐1 and si‐AS1‐2), both of which efficiently suppressed the *UPK1A-AS1* expression (Figure 7C). Under normoxic conditions, Annexin V/PI staining revealed that sorafenib markedly promoted apoptosis in MHCC‐97H cells, and *UPK1A-AS1* knockdown further increased the sensitivity of HCC cells to sorafenib. In contrast, hypoxia substantially reduced sorafenib‐induced apoptosis, indicating hypoxia‐mediated resistance to sorafenib. Notably, silencing *UPK1A-AS1* under hypoxic conditions effectively restored the apoptotic response, thereby reversing hypoxia‐induced sorafenib resistance (Figure 7D,E). To further validate *UPK1A-AS1* expression in HCC, we examined its expression by qRT‐PCR in 19 pairs of HCC tissues and matched adjacent nontumor tissues. *UPK1A-AS1* was significantly upregulated in HCC tissues relative to the matched adjacent nontumor tissues (Figure 7F), establishing its aberrant expression in patient‐derived HCC specimens and providing a clinical context for its functional role in HCC. Collectively, these results demonstrate that *UPK1A-AS1* is upregulated in HCC and induced by hypoxia in HCC cells and that it attenuates sorafenib‐induced apoptosis, thereby contributing to hypoxia‐associated sorafenib resistance.

### 3.7. *UPK1A-AS1* Inhibition Potentiates Sorafenib Efficacy *In Vivo*

**Figure 8:**
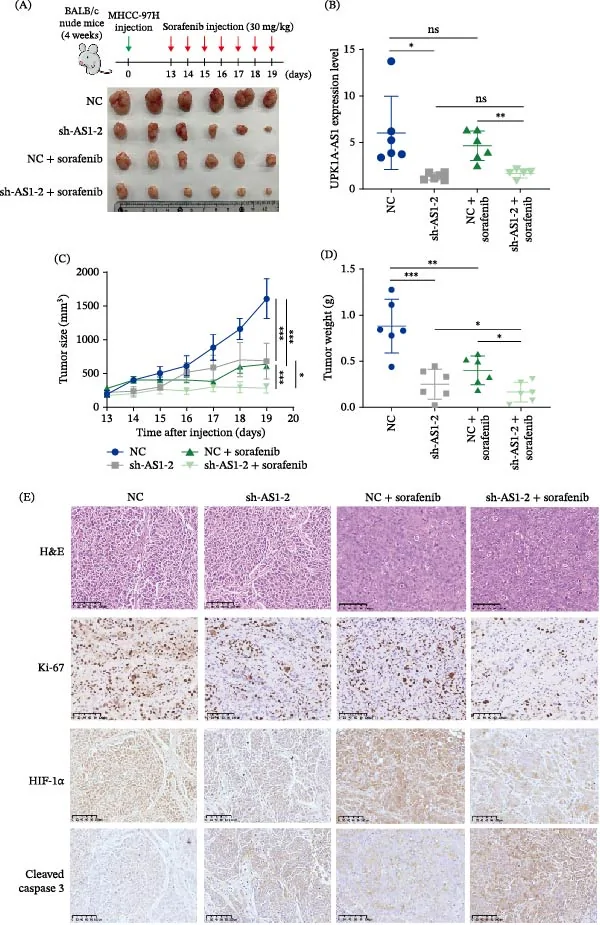
*UPK1A-AS1* inhibition potentiates the therapeutic effect of sorafenib in vivo. (A) Representative images of xenograft tumors derived from MHCC‐97H cells subjected to different treatments. (B) Quantitative RT‐PCR analysis of *UPK1A-AS1* expression levels in xenograft tumor tissues. (C) Tumor growth curves recorded during the treatment period. (D) Final tumor weights measured at the endpoint of the experiment. (E) Representative immunohistochemical images of Ki‐67, HIF‐1α, and cleaved caspase‐3 in xenograft tumors.  ^∗^ *p* < 0.05,  ^∗∗^ *p* < 0.01, and  ^∗∗∗^ *p* < 0.001.

To assess the in vivo function of *UPK1A-AS1* in HCC growth and response to sorafenib, MHCC‐97H cells with stable *UPK1A-AS1* knockdown were subcutaneously implanted into nude mice (Figure 8A and Figure S4A). Compared with that in the control tumors, the *UPK1A-AS1* expression was markedly lower in the knockdown tumors (Figure 8B). In the absence of treatment, *UPK1A-AS1* depletion alone significantly suppressed tumor growth. Sorafenib monotherapy also slowed tumor progression, although to a lesser extent. Strikingly, the combination of *UPK1A-AS1* knockdown and sorafenib administration had the strongest inhibitory effect on tumor development (Figure 8A). The tumor growth curves revealed sustained suppression in the combination group (Figure 8C), and the endpoint tumor weights were significantly lower than those in either monotherapy group (Figure 8D). These data demonstrate that *UPK1A-AS1* promotes HCC growth in vivo and attenuates the antitumor efficacy of sorafenib, whereas its inhibition markedly enhances therapeutic sensitivity.

To further assess the effects of *UPK1A-AS1* knockdown and sorafenib treatment on tumor apoptosis and hypoxia‐associated signaling, xenograft tumors from each group were subjected to histological and immunohistochemical analyses. Ki‐67 immunostaining revealed diminished proliferative activity in the UPK1A‐AS1‐knockdown and sorafenib‐treated groups compared with the control group, with the lowest proportion of Ki‐67‐positive cells observed in the tumors from the combined treatment group. Immunohistochemical analysis of HIF‐1α and cleaved caspase‐3 further showed that *UPK1A-AS1* knockdown reduced HIF‐1α expression and enhanced sorafenib‐induced apoptosis in xenograft tumors. (Figure 8E). Together, these results indicate that *UPK1A-AS1* depletion enhances the proapoptotic and antiproliferative effects of sorafenib and attenuates hypoxia‐associated signaling in vivo, further supporting the notion that *UPK1A-AS1* inhibition enhances the therapeutic efficacy of sorafenib in HCC.

## 4. Discussion

In the present study, we conducted a comprehensive evaluation of *UPK1A-AS1* across multiple cancer types to elucidate its clinical relevance and potential biological functions. By integrating transcriptomic profiles with survival outcomes, genomic features, and tumor microenvironmental characteristics, we observed that *UPK1A-AS1* expression is significantly associated with prognosis and treatment‐related phenotypes in a tumor context‐dependent manner. In addition to survival analyses, we systematically assessed immune and stromal components via ESTIMATE‐ and TIMER‐based approaches and identified distinct immune microenvironmental patterns linked to *UPK1A-AS1* expression across cancers. In addition, hypoxia‐related analyses and experimental validation revealed that *UPK1A-AS1* is responsive to hypoxic stress and functionally contributes to hypoxia‐associated drug tolerance. Collectively, these results suggest that *UPK1A-AS1* is not only a prognostic marker but also linked to tumor stress adaptation and therapeutic response in a cancer type‐dependent manner.

Notably, our study identified an association between *UPK1A-AS1* and drug resistance‐related pathways. KEGG enrichment analysis of genes that presented significant co‐mutation with *UPK1A-AS1* in pan‐cancer samples revealed “platinum drug resistance” among the enriched pathways, indicating that *UPK1A-AS1*‐associated genomic alterations may converge on chemoresistance‐related programs. This observation is supported by mechanistic evidence from pancreatic cancer, where cancer‐associated fibroblast‐derived IL‐8 was shown to induce *UPK1A-AS1* expression through NF‐κB signaling, thereby promoting oxaliplatin resistance by enhancing DNA double‐strand break repair via Ku70/Ku80‐dependent nonhomologous end joining (NHEJ) [7]. In this context, *UPK1A-AS1* functions as a molecular scaffold that stabilizes Ku70/Ku80 interactions and facilitates the recruitment of the NHEJ machinery to damaged chromatin, ultimately reducing chemotherapy‐induced apoptosis. These findings are consistent with our observation that *UPK1A-AS1* is associated with resistance‐related molecular programs, particularly in tumors treated with platinum‐based chemotherapeutic agents. Importantly, recent mechanistic studies further support the concept that NHEJ efficiency can be modulated by accessory factors, including lncRNAs, which may stabilize DNA end synapses and promote efficient end joining [36, 37]. More broadly, exosomal lncRNAs have emerged as mediators of therapeutic resistance through intercellular communication and the regulation of DNA repair, apoptosis suppression, drug efflux, epithelial–mesenchymal transition, and tumor microenvironment remodeling [38]. This notion is further supported by a recent genetic study in mouse intestinal tumoroids showing that *Mir34a* loss reduced 5‐fluorouracil‐induced apoptosis, whereas deletion of *Csf1r*, a direct target of *Mir34a*, increased apoptosis and counteracted *Mir34a* inactivation‐associated resistance, demonstrating that noncoding RNA networks can modulate chemosensitivity through apoptosis control [39]. Taken together, these data support the cautious interpretation that *UPK1A-AS1* may contribute to a repair‐competent cellular state that compromises the efficacy of platinum‐based chemotherapy in specific tumor contexts.

In parallel, our results highlight hypoxia as an important upstream regulator and functional context for *UPK1A-AS1*. Previous transcriptomic profiling studies identified *UPK1A-AS1* as a prominent hypoxia‐induced lncRNA in lung cancer cells, with experimental evidence that HIF‐1 α perturbation attenuates its upregulation under hypoxia [28]. In line with these observations, we found that hypoxia attenuated sorafenib‐induced apoptosis, whereas silencing *UPK1A-AS1* restored the apoptotic response in HCC cells. Moreover, in vivo experiments using an HCC xenograft model revealed that *UPK1A-AS1* depletion suppressed tumor growth and significantly enhanced the antitumor efficacy of sorafenib. Our clinical validation further confirmed that *UPK1A-AS1* is upregulated in human HCC tissues. These findings suggest that *UPK1A-AS1* may act as a mediator of hypoxia‐associated therapeutic tolerance, extending its relevance beyond platinum‐based chemotherapy to targeted therapies while highlighting its potential as a therapeutic target in HCC.

Interestingly, the functional role of *UPK1A-AS1* appears to be highly context‐dependent. In contrast to its resistance‐associated and protumorigenic roles reported in pancreatic and liver cancers, *UPK1A-AS1* has been described as a tumor‐suppressive lncRNA in ESCC, where it is downregulated and inhibits proliferation and invasion by acting as a competing endogenous RNA for *miR-1248* [8]. This discrepancy underscores an important point for interpretation: *UPK1A-AS1* should not be viewed as a uniform oncogenic or tumor‐suppressive factor across cancers. Instead, its biological effects are likely shaped by tissue‐specific transcriptional programs, cellular localization, and microenvironmental pressures such as hypoxia and stromal inflammation.

Several limitations of this study should be acknowledged. First, although our analyses established robust associations between *UPK1A-AS1* expression and clinical or therapeutic phenotypes, causal relationships require further tumor‐specific mechanistic validation. Second, bulk transcriptomic data cannot fully resolve the cellular sources of *UPK1A-AS1* expression or disentangle tumor cell‐intrinsic effects from stromal or immune contributions. Of note, data from the CIDE database indicated that *UPK1A-AS1* is expressed in both tumor cells and T‐cell subsets, suggesting that the observed negative correlations between *UPK1A-AS1* expression and immune infiltration may not directly reflect immune suppression mediated by tumor‐derived *UPK1A-AS1*. The importance of cellular origin is further illustrated by recent genetic evidence showing that myeloid‐specific *Mir34a* loss promotes the accumulation of tumor‐supportive M2‐like macrophages and CD4^+^Foxp3^+^ regulatory T cells while reducing Granzyme B‐positive CD8^+^ T cells in intestinal tumors [40]. This example demonstrates that noncoding RNA‐associated immune phenotypes may originate from specific nonmalignant cellular compartments rather than from tumor cells alone. Accordingly, cell type‐resolved analyses will be necessary to determine whether *UPK1A-AS1* directly regulates immune‐cell states or whether its bulk‐tissue associations primarily reflect differences in cellular composition. Future studies integrating single‐cell or spatial transcriptomic approaches will be valuable to clarify these issues. Third, the associations between *UPK1A-AS1* expression and immune infiltration, immune checkpoint molecules, or other immune‐related genes were derived from bulk transcriptomic data; therefore, these findings demonstrate correlations rather than direct causal regulation, and further experimental validation is required to establish the functional relevance. Fourth, the prognostic analyses for HCC in this study were derived from TCGA‐LIHC cohort, which mainly comprises patients who underwent surgical resection rather than sorafenib treatment. Future validation in independent cohorts of sorafenib‐treated HCC patients would be valuable to assess the predictive value of *UPK1A-AS1* for targeted therapy response. Finally, given the context‐dependent nature of *UPK1A-AS1* function, its translational potential as a biomarker or therapeutic target should be evaluated cautiously and in a cancer type‐specific manner.

Our study provides a comprehensive pan‐cancer perspective on *UPK1A-AS1* and demonstrates that this lncRNA is closely associated with prognosis, tumor microenvironmental features, and therapeutic resistance. By integrating multiomics analyses with functional experiments, we further suggest that *UPK1A-AS1* participates in tumor stress adaptation and may shape treatment responsiveness in a context‐dependent manner. These findings highlight *UPK1A-AS1* as a biologically and clinically relevant lncRNA whose roles across cancer types warrant further mechanistic dissection and careful evaluation for tumor‐specific translational applications.

## 5. Conclusions

In conclusion, our pan‐cancer analyses identified *UPK1A-AS1* as a clinically relevant lncRNA characterized by dysregulated expression across multiple malignancies and significant associations with poor prognosis, genomic alterations, and immune microenvironment heterogeneity in a cancer type‐dependent manner. In HCC, we further demonstrated that hypoxia induces *UPK1A-AS1* expression and promotes sorafenib resistance by suppressing apoptosis. Our findings provide a foundation for future investigations into the context‐dependent roles and functional mechanisms of *UPK1A-AS1* in different tumor types.

**Nomenclature**
lncRNAs: Long noncoding RNAs
*UPK1A-AS1*: UPK1A antisense RNA 1
HCC: Hepatocellular carcinoma
TCGA: The Cancer Genome Atlas Project
GTEx: Genotype‐Tissue Expression Project
CIDE: Cancer immunology data engine
cBioPortal: cBioPortal for cancer genomics
ESCC: Esophageal squamous cell carcinoma
CNVs: Copy‐number variations
DEGs: Differentially expressed genes
KEGG: Kyoto Encyclopedia of Genes and Genomes
GO: Gene Ontology
TIMER: Tumor immune estimation resource
ESTIMATE: Estimation of STromal and Immune cells in MAlignant Tumours using Expression data
OS: Overall survival
PFS: Progression‐free survival
DFS: Disease‐free survival
DSS: Disease‐specific survival
DMEM: Dulbecco’s Modified Eagle’s Medium
FBS: Fetal bovine serum
HRP: Horseradish peroxidase
DAB: Diaminobenzidine
ESCA: Esophageal carcinoma
LUAD: Lung adenocarcinoma
OV: Ovarian cancer
PAAD: Pancreatic ductal adenocarcinoma
BRCA: Breast cancer
LUSC: Lung squamous cell carcinoma
SARC: Sarcoma
UCEC: Uterine corpus endometrial carcinoma
LIHC: Liver hepatocellular carcinoma
LAML: Acute myeloid leukemia
MESO: Mesothelioma
CHOL: Cholangiocarcinoma
UCS: Uterine carcinosarcoma
UVM: Uveal melanoma
BLCA: Bladder carcinoma
TGCT: Testicular germ cell tumor
SKCM: Skin cutaneous melanoma
GBM: Glioblastoma multiforme
GBMLGG: Glioma
LGG: Lower grade glioma
KIRP: Kidney renal papillary cell carcinoma
KIPAN: Pan‐kidney cohort
PRAD: Prostate adenocarcinoma
KIRC: Kidney renal clear cell carcinoma
KICH: Kidney chromophobe
COADREAD: Colon and rectal adenocarcinoma
STES: Stomach and esophageal carcinoma
COAD: Colon adenocarcinoma
WT: Wilms tumor
THCA: Thyroid carcinoma
STAD: Stomach adenocarcinoma
ACC: Adrenocortical carcinoma
HNSC: Head and neck squamous cell carcinoma
READ: Rectum adenocarcinoma
PCPG: Pheochromocytoma and paraganglioma
TMB: Tumor mutational burden
MSI: Microsatellite instability
NEO: Neoantigens
ROC: Receiver operating characteristic
AUC: Area under the curve
NHEJ: Nonhomologous end joining.

## Author Contributions

The coordination, conception, and design of the study were provided by Dong‐Yan Zhang and Quan‐Hui Hu. The experiments were performed by Ze‐Kai Li, Min‐Rui Luo, Shu‐Jie Fang, and Yi‐Da Wu. Shu‐Jie Fang and Yi‐Da Wu assisted in data collection. Data analysis was carried out by Ze‐Kai Li, Min‐Rui Luo, and Dong‐Yan Zhang. Ze‐Kai Li and Min‐Rui Luo wrote the manuscript, were responsible for the manuscript revision, and confirm the authenticity of all the raw data.

## Funding

This research was supported by the National Natural Science Foundation of China (Grant 82272719), the Natural Science Foundation of Guangdong Province (Grants 2023A1515012724 and 2024A1515013249), and the Science and Technology Projects in Guangzhou (Grant 2024A04J5205).

## Disclosure

All the authors read and approved the final manuscript.

## Ethics Statement

All experiments were conducted in accordance with the ethical standards of the Ethics Committee of Nanfang Hospital, Southern Medical University.

## Consent

The authors have nothing to report.

## Conflicts of Interest

The authors declare no conflicts of interest.

## Supporting Information

Additional supporting information can be found online in the Supporting Information section.

## Supporting information


**Supporting Information** Figure S1: Single‐cell distribution of *UPK1A-AS1* expression across tumor microenvironment cell types. Figure S2: Association between *UPK1A-AS1* expression and clinicopathological characteristics across cancers. (A) Expression levels of *UPK1A-AS1* in tumors with different distant metastasis statuses. (B) Expression levels of *UPK1A-AS1* across different histological grades. (C) Expression levels of *UPK1A-AS1* in male and female samples.  ^∗^ *p* < 0.05,  ^∗∗^ *p* < 0.01,  ^∗∗∗∗^ *p* < 0.0001, and  ^−^ *p* ≥ 0.05. Figure S3: Survival analysis of *UPK1A-AS1* alterations across cancers. (A) Disease‐free survival analysis of patients with or without *UPK1A-AS1* alterations. (B) Disease‐specific survival analysis of patients with or without *UPK1A-AS1* alterations. Figure S4: Photographs of tumor‐bearing nude mice under different treatment conditions. Table S1: Co‐mutation genes associated with *UPK1A-AS1* identified from cBioPortal and used for pathway enrichment analysis. Table S2: Differentially expressed genes associated with *UPK1A-AS1* in BLCA, UCEC, and PRAD.

## Data Availability

The data that support the findings of this study are available from the corresponding author upon reasonable request.
