# Supplementary material for: Integrative Pan‐Cancer Characterization of lncRNA UPK1A‐AS1 and Its Role in Hypoxia‐Associated Sorafenib Resistance in Hepatocellular Carcinoma
Source: Anal Cell Pathol (Amst). 2026 Sep 1;2026:1554526. doi: 10.1155/ancp/1554526 (PMC13532061; doi:10.1155/ancp/1554526)
Supplement: Supplementary file 1 [file ANCP-2026-1554526-s001.zip › Supplement Figures.docx]

.

**Supplementary Figure S1. Single-cell distribution of *UPK1A-AS1* expression across tumor microenvironment cell types.**

Trajectory plot showing the expression pattern of *UPK1A-AS1* across major cell lineages within the tumor microenvironment, including immune, stromal, and cancer cell populations. The cell differentiation trajectories are depicted with branch connections, and the expression levels are indicated by the color intensity on the scale bar. Trajectory plot showing the expression pattern of *UPK1A-AS1* across major cell lineages within the tumor microenvironment, including immune, stromal, and cancer cell populations. The cell differentiation trajectories are depicted with branch connections, and the expression levels are indicated by the color intensity on the scale bar.

**Supplementary Figure S2. Association of *UPK1A-AS1* expression with clinicopathological characteristics across cancers.**

(A) Boxplot showing the expression levels of *UPK1A-AS1* in tumors with different distant metastasis statuses (M stage). (B) Boxplot illustrating *UPK1A-AS1* expression across different histological grades (G1–G4). (C) Boxplot showing *UPK1A-AS1* expression in male and female samples. All comparisons in (A)–(C) were performed using the unpaired Student's t‑test. * p < 0.05, ** p < 0.01, **** p < 0.0001, – p ≥ 0.05. A p-value less than 0.05 was considered significant.

**Supplementary Figure S3. Survival analysis of *UPK1A-AS1* alterations across cancers.**

(A) Kaplan–Meier curves comparing disease-free survival (DFS) between patients with and without *UPK1A-AS1* alterations. (B) Kaplan–Meier curves comparing disease-specific survival (DSS) between the altered and unaltered groups. A p value less than 0.05 was considered significant.

**Supplementary Figure S4. Photographs of tumor-bearing nude mice under different treatment conditions.**

Whole-body views of tumor-bearing nude mice receiving control, *UPK1A-AS1* knockdown, sorafenib, or combined treatment.
