# Supplementary material for: Integrative Pan‐Cancer Characterization of lncRNA UPK1A‐AS1 and Its Role in Hypoxia‐Associated Sorafenib Resistance in Hepatocellular Carcinoma
Source: Anal Cell Pathol (Amst). 2026 Sep 1;2026:1554526. doi: 10.1155/ancp/1554526 (PMC13532061; doi:10.1155/ancp/1554526)
Supplement: Supplementary file 1 [file ANCP-2026-1554526-s001.zip › Table S1.docx]

| **Table S1 Co-mutation genes associated with *UPK1A-AS1* identified from cBioPortal used for pathway enrichment analysis.** | | | | | | | |
| --- | --- | --- | --- | --- | --- | --- | --- |
| Gene | Cytoband | Altered group | Unaltered group | Log2 Ratio | p-Value | q-Value | Enriched in |
| HSPB6 | 19q13.12 | 220 (95.65%) | 9 (0.08%) | >10 | ＜1E-300 | 0 | Altered group |
| LINC01766 | 19q13.12 | 223 (96.96%) | 5 (0.05%) | >10 | ＜1E-300 | 0 | Altered group |
| TEX14BP | 19q13.12 | 215 (93.48%) | 3 (0.03%) | >10 | ＜1E-300 | 0 | Altered group |
| TMEM147-AS1 | 19q13.12 | 221 (96.09%) | 9 (0.08%) | >10 | ＜1E-300 | 0 | Altered group |
| SDHAF1 | 19q13.12 | 199 (86.52%) | 10 (0.09%) | 9.86 | ＜1E-300 | 0 | Altered group |
| LOC101927572 | 19q13.12 | 196 (85.22%) | 10 (0.09%) | 9.84 | ＜1E-300 | 0 | Altered group |
| OVOL3 | 19q13.12 | 190 (82.61%) | 13 (0.12%) | 9.41 | ＜1E-300 | 0 | Altered group |
| LINC01531 | 19q13.12 | 214 (93.04%) | 17 (0.16%) | 9.2 | ＜1E-300 | 0 | Altered group |
| COX6B1 | 19q13.12 | 229 (99.57%) | 25 (0.23%) | 8.74 | ＜1E-300 | 0 | Altered group |
| PSENEN | 19q13.12 | 220 (95.65%) | 26 (0.24%) | 8.62 | ＜1E-300 | 0 | Altered group |
| HCST | 19q13.12 | 205 (89.13%) | 25 (0.23%) | 8.58 | ＜1E-300 | 0 | Altered group |
| ETV2 | 19q13.12 | 226 (98.26%) | 30 (0.28%) | 8.46 | ＜1E-300 | 0 | Altered group |
| COX7A1 | 19q13.12 | 185 (80.43%) | 27 (0.25%) | 8.32 | ＜1E-300 | 0 | Altered group |
| ZNF30-AS1 | 19q13.11 | 196 (85.22%) | 29 (0.27%) | 8.3 | ＜1E-300 | 0 | Altered group |
| LINC00904 | 19q13.11 | 193 (83.91%) | 29 (0.27%) | 8.28 | ＜1E-300 | 0 | Altered group |
| CYKILR |  | 172 (74.78%) | 26 (0.24%) | 8.27 | 3.62E-296 | 1.25E-293 | Altered group |
| LINC00665 | 19q13.12 | 172 (74.78%) | 26 (0.24%) | 8.27 | 3.62E-296 | 1.25E-293 | Altered group |
| ACOT4P1 |  | 168 (73.04%) | 27 (0.25%) | 8.18 | 1.48E-286 | 4.90E-284 | Altered group |
| ZNF566-AS1 | 19q13.12 | 162 (70.43%) | 27 (0.25%) | 8.13 | 1.18E-273 | 3.66E-271 | Altered group |
| HPN-AS1 | 19q13.11-q13.12 | 203 (88.26%) | 34 (0.32%) | 8.12 | ＜1E-300 | 0 | Altered group |
| KRTDAP | 19q13.12 | 217 (94.35%) | 37 (0.34%) | 8.1 | ＜1E-300 | 0 | Altered group |
| TMEM147 | 19q13.12 | 221 (96.09%) | 39 (0.36%) | 8.05 | ＜1E-300 | 0 | Altered group |
| U2AF1L4 | 19q13.12 | 221 (96.09%) | 39 (0.36%) | 8.05 | ＜1E-300 | 0 | Altered group |
| TYROBP | 19q13.12 | 205 (89.13%) | 37 (0.34%) | 8.01 | ＜1E-300 | 0 | Altered group |
| POLR2I | 19q13.12 | 189 (82.17%) | 35 (0.33%) | 7.98 | ＜1E-300 | 0 | Altered group |
| LINC01801 | 19q13.11 | 191 (83.04%) | 36 (0.34%) | 7.95 | ＜1E-300 | 0 | Altered group |
| SCGB2B3P | 19q13.11 | 179 (77.83%) | 34 (0.32%) | 7.94 | 4.20E-305 | 1.65E-302 | Altered group |
| FXYD7 | 19q13.12 | 204 (88.70%) | 40 (0.37%) | 7.89 | ＜1E-300 | 0 | Altered group |
| HAMP | 19q13.12 | 205 (89.13%) | 41 (0.38%) | 7.87 | ＜1E-300 | 0 | Altered group |
| ZNF529-AS1 | 19q13.12 | 162 (70.43%) | 34 (0.32%) | 7.8 | 4.15E-268 | 1.24E-265 | Altered group |
| THAP8 | 19q13.12 | 196 (85.22%) | 42 (0.39%) | 7.77 | ＜1E-300 | 0 | Altered group |
| UPK1A | 19q13.12 | 229 (99.57%) | 50 (0.47%) | 7.74 | ＜1E-300 | 0 | Altered group |
| SCGB1B2P | 19q13.11 | 178 (77.39%) | 39 (0.36%) | 7.73 | 4.74E-299 | 1.77E-296 | Altered group |
| LIN37 | 19q13.12 | 220 (95.65%) | 49 (0.46%) | 7.71 | ＜1E-300 | 0 | Altered group |
| FXYD1 | 19q13.12 | 204 (88.70%) | 46 (0.43%) | 7.69 | ＜1E-300 | 0 | Altered group |
| ZNF567-DT | 19q13.12 | 158 (68.70%) | 37 (0.34%) | 7.64 | 1.61E-257 | 4.56E-255 | Altered group |
| TBCB | 19q13.12 | 189 (82.17%) | 45 (0.42%) | 7.61 | 1.11e-319 | 4.93e-317 | Altered group |
| ZNF790-AS1 | 19q13.12 | 156 (67.83%) | 39 (0.36%) | 7.54 | 5.70E-252 | 1.60E-249 | Altered group |
| ALKBH6 | 19q13.12 | 197 (85.65%) | 50 (0.47%) | 7.52 | ＜1E-300 | 0 | Altered group |
| IGFLR1 | 19q13.12 | 220 (95.65%) | 56 (0.52%) | 7.52 | ＜1E-300 | 0 | Altered group |
| FXYD3 | 19q13.12 | 203 (88.26%) | 54 (0.50%) | 7.45 | ＜1E-300 | 0 | Altered group |
| CAPNS1 | 19q13.12 | 187 (81.30%) | 53 (0.49%) | 7.36 | 0.00E+00 | 5.05E-307 | Altered group |
| FFAR1 | 19q13.12 | 209 (90.87%) | 60 (0.56%) | 7.34 | ＜1E-300 | 0 | Altered group |
| USF2 | 19q13.12 | 205 (89.13%) | 60 (0.56%) | 7.32 | ＜1E-300 | 0 | Altered group |
| FFAR2 | 19q13.12 | 215 (93.48%) | 68 (0.63%) | 7.2 | ＜1E-300 | 0 | Altered group |
| SYNE4 | 19q13.12 | 199 (86.52%) | 66 (0.62%) | 7.14 | ＜1E-300 | 0 | Altered group |
| ZBTB32 | 19q13.12 | 225 (97.83%) | 76 (0.71%) | 7.11 | ＜1E-300 | 0 | Altered group |
| NFKBID | 19q13.12 | 206 (89.57%) | 70 (0.65%) | 7.1 | ＜1E-300 | 0 | Altered group |
| FXYD5 | 19q13.12 | 204 (88.70%) | 71 (0.66%) | 7.07 | ＜1E-300 | 0 | Altered group |
| SCGB2B2 | 19q13.12 | 178 (77.39%) | 62 (0.58%) | 7.07 | 5.14E-284 | 1.68E-281 | Altered group |
| PROSER3 | 19q13.12 | 220 (95.65%) | 77 (0.72%) | 7.06 | ＜1E-300 | 0 | Altered group |
| SCN1B | 19q13.11 | 199 (86.52%) | 73 (0.68%) | 6.99 | ＜1E-300 | 0 | Altered group |
| LINC01535 | 19q13.12 | 144 (62.61%) | 53 (0.49%) | 6.99 | 4.21E-219 | 1.03E-216 | Altered group |
| ZNF146 | 19q13.1 | 182 (79.13%) | 68 (0.63%) | 6.96 | 2.34E-289 | 7.88E-287 | Altered group |
| RBM42 | 19q13.12 | 225 (97.83%) | 87 (0.81%) | 6.91 | ＜1E-300 | 0 | Altered group |
| FAM187B | 19q13.12 | 203 (88.26%) | 91 (0.85%) | 6.7 | ＜1E-300 | 0 | Altered group |
| HAUS5 | 19q13.12 | 224 (97.39%) | 101 (0.94%) | 6.69 | ＜1E-300 | 0 | Altered group |
| GAPDHS | 19q13.12 | 221 (96.09%) | 101 (0.94%) | 6.67 | ＜1E-300 | 0 | Altered group |
| ZNF793-AS1 | 19q13.12 | 135 (58.70%) | 62 (0.58%) | 6.67 | 4.98E-197 | 1.13E-194 | Altered group |
| ZNF565 | 19q13.12 | 182 (79.13%) | 84 (0.78%) | 6.66 | 6.47E-281 | 2.09E-278 | Altered group |
| LRFN3 | 19q13.12 | 204 (88.70%) | 96 (0.89%) | 6.63 | ＜1E-300 | 0 | Altered group |
| WTIP | 19q13.11 | 177 (76.96%) | 84 (0.78%) | 6.62 | 3.88E-270 | 1.17E-267 | Altered group |
| FFAR3 | 19q13.12 | 212 (92.17%) | 101 (0.94%) | 6.61 | ＜1E-300 | 0 | Altered group |
| LGI4 | 19q13.12 | 204 (88.70%) | 98 (0.91%) | 6.6 | ＜1E-300 | 0 | Altered group |
| PRODH2 | 19q13.12 | 215 (93.48%) | 105 (0.98%) | 6.58 | ＜1E-300 | 0 | Altered group |
| CLIP3 | 19q13.12 | 197 (85.65%) | 98 (0.91%) | 6.55 | 7.72E-308 | 3.13E-305 | Altered group |
| DMKN | 19q13.12 | 219 (95.22%) | 110 (1.03%) | 6.54 | ＜1E-300 | 0 | Altered group |
| PDCD2L | 19q13.11 | 174 (75.65%) | 90 (0.84%) | 6.49 | 6.13E-261 | 1.76E-258 | Altered group |
| SBSN | 19q13.12 | 220 (95.65%) | 116 (1.08%) | 6.47 | ＜1E-300 | 0 | Altered group |
| HPN | 19q13.11 | 200 (86.96%) | 106 (0.99%) | 6.46 | 3.90e-311 | 0.00E+00 | Altered group |
| ZNF302 | 19q13.11 | 180 (78.26%) | 96 (0.89%) | 6.45 | 6.84E-271 | 2.09E-268 | Altered group |
| ZNF571-AS1 | 19q13.12 | 134 (58.26%) | 73 (0.68%) | 6.42 | 5.58E-190 | 1.20E-187 | Altered group |
| ZNF30 | 19q13.13 | 196 (85.22%) | 118 (1.10%) | 6.28 | 1.43E-296 | 5.10E-294 | Altered group |
| ZNF181 | 19q13.11 | 185 (80.43%) | 111 (1.03%) | 6.28 | 4.45E-275 | 1.40E-272 | Altered group |
| ZNF260 | 19q13.12 | 164 (71.30%) | 99 (0.92%) | 6.27 | 1.76E-236 | 4.71E-234 | Altered group |
| CEBPA-DT | 19q13.11 | 145 (63.04%) | 88 (0.82%) | 6.26 | 3.93E-204 | 9.25E-202 | Altered group |
| LSR | 19q13.12 | 207 (90.00%) | 129 (1.20%) | 6.23 | 6.24e-318 | 2.72e-315 | Altered group |
| WDR87BP | 19q13.13 | 128 (55.65%) | 84 (0.78%) | 6.15 | 2.29E-174 | 4.53E-172 | Altered group |
| ZNF792 | 19q13.11 | 199 (86.52%) | 134 (1.25%) | 6.11 | 6.15E-297 | 2.23E-294 | Altered group |
| ZNF566 | 19q13.12 | 166 (72.17%) | 112 (1.04%) | 6.11 | 3.28E-235 | 8.68E-233 | Altered group |
| GRAMD1A | 19q13.11 | 200 (86.96%) | 138 (1.29%) | 6.08 | 1.09E-297 | 4.02E-295 | Altered group |
| ZNF850 | 19q13.12 | 160 (69.57%) | 114 (1.06%) | 6.03 | 1.30E-222 | 3.25E-220 | Altered group |
| ZNF529 | 19q13.12 | 164 (71.30%) | 119 (1.11%) | 6.01 | 1.42E-228 | 3.64E-226 | Altered group |
| KCTD15 | 19q13.11 | 155 (67.39%) | 114 (1.06%) | 5.99 | 5.78E-213 | 1.40E-210 | Altered group |
| DPY19L3-DT | 19q13.11 | 135 (58.70%) | 102 (0.95%) | 5.95 | 3.41E-180 | 7.05E-178 | Altered group |
| RAB4B-EGLN2 | 19q13.2 | 70 (30.43%) | 53 (0.49%) | 5.95 | 3.66E-88 | 3.72E-86 | Altered group |
| APLP1 | 19q13.12 | 210 (91.30%) | 159 (1.48%) | 5.95 | 1.14e-313 | 4.88e-311 | Altered group |
| CEBPG | 19q13.11 | 146 (63.48%) | 112 (1.04%) | 5.93 | 9.75E-197 | 2.19E-194 | Altered group |
| MIA-RAB4B | 19q13.2 | 70 (30.43%) | 54 (0.50%) | 5.92 | 8.29E-88 | 8.38E-86 | Altered group |
| ZFP14 | 19q13.12 | 172 (74.78%) | 134 (1.25%) | 5.9 | 4.93E-239 | 1.33E-236 | Altered group |
| ZFP82 | 19q13.12 | 169 (73.48%) | 133 (1.24%) | 5.89 | 2.17E-233 | 5.67E-231 | Altered group |
| CEBPA | 19q13.11 | 145 (63.04%) | 114 (1.06%) | 5.89 | 3.55E-194 | 7.81E-192 | Altered group |
| UBA2 | 19q13.11 | 177 (76.96%) | 140 (1.30%) | 5.88 | 4.72E-247 | 1.31E-244 | Altered group |
| LINC01782 | 19q13.11 | 128 (55.65%) | 102 (0.95%) | 5.87 | 1.09E-167 | 2.02E-165 | Altered group |
| CYP2G1P | 19q13.2 | 64 (27.83%) | 51 (0.48%) | 5.87 | 2.01E-79 | 1.98E-77 | Altered group |
| MAG | 19q13.1 | 206 (89.57%) | 167 (1.56%) | 5.85 | 3.90E-301 | 1.50E-298 | Altered group |
| ZNF599 | 19q13.11 | 186 (80.87%) | 151 (1.41%) | 5.84 | 5.94E-262 | 1.73E-259 | Altered group |
| GPI | 19q13.11 | 174 (75.65%) | 143 (1.33%) | 5.83 | 6.42E-240 | 1.76E-237 | Altered group |
| LINC01533 | 19q13.11 | 129 (56.09%) | 106 (0.99%) | 5.83 | 4.45E-168 | 8.39E-166 | Altered group |
| LSM14A | 19q13.11 | 169 (73.48%) | 141 (1.31%) | 5.81 | 1.26E-230 | 3.27E-228 | Altered group |
| RGS9BP | 19q13.11 | 137 (59.57%) | 115 (1.07%) | 5.8 | 2.87E-179 | 5.87E-177 | Altered group |
| KIRREL2 | 19q13.12 | 210 (91.30%) | 179 (1.67%) | 5.77 | 1.11E-306 | 4.43E-304 | Altered group |
| ZNF461 | 19q13.13 | 163 (70.87%) | 140 (1.30%) | 5.76 | 3.02E-219 | 7.49E-217 | Altered group |
| ARHGAP33 | 19q13.12 | 217 (94.35%) | 189 (1.76%) | 5.74 | 4.88e-321 | 2.21e-318 | Altered group |
| CD22 | 19q13.12 | 209 (90.87%) | 192 (1.79%) | 5.67 | 5.26E-300 | 2.00E-297 | Altered group |
| FAAP24 | 19q13.11 | 141 (61.30%) | 130 (1.21%) | 5.66 | 1.42E-181 | 2.97E-179 | Altered group |
| ZNF829 | 19q13.12 | 154 (66.96%) | 143 (1.33%) | 5.65 | 3.14E-201 | 7.25E-199 | Altered group |
| LINC01480 | 19q13.2 | 43 (18.70%) | 40 (0.37%) | 5.65 | 4.64E-51 | 4.24E-49 | Altered group |
| ATP4A | 19q13.12 | 221 (96.09%) | 207 (1.93%) | 5.64 | ＜1E-300 | 0 | Altered group |
| PDCD5 | 19q13.11 | 138 (60.00%) | 130 (1.21%) | 5.63 | 3.22E-176 | 6.48E-174 | Altered group |
| NUDT19 | 19q13.11 | 136 (59.13%) | 130 (1.21%) | 5.61 | 1.11E-172 | 2.18E-170 | Altered group |
| CYP2B7P | 19q13.2 | 64 (27.83%) | 61 (0.57%) | 5.61 | 3.40E-76 | 3.28E-74 | Altered group |
| C19ORF33 | 19q13.2 | 125 (54.35%) | 123 (1.15%) | 5.57 | 1.06E-155 | 1.79E-153 | Altered group |
| ZNF383 | 19q13.13 | 145 (63.04%) | 143 (1.33%) | 5.56 | 9.09E-185 | 1.93E-182 | Altered group |
| ZNF345 | 19q13.12 | 155 (67.39%) | 154 (1.44%) | 5.55 | 1.02E-199 | 2.33E-197 | Altered group |
| ZNF790 | 19q13.12 | 158 (68.70%) | 159 (1.48%) | 5.53 | 8.34E-204 | 1.94E-201 | Altered group |
| PPP1R14A | 19q13.1 | 124 (53.91%) | 125 (1.16%) | 5.53 | 2.11E-153 | 3.55E-151 | Altered group |
| LINC03103 | 19q12 | 127 (55.22%) | 130 (1.21%) | 5.51 | 4.70E-157 | 8.01E-155 | Altered group |
| LNROP | 19q13.2 | 40 (17.39%) | 41 (0.38%) | 5.51 | 2.31E-46 | 2.03E-44 | Altered group |
| ZNF382 | 19q13.12 | 162 (70.43%) | 167 (1.56%) | 5.5 | 5.91E-209 | 1.42E-206 | Altered group |
| ZNF567 | 19q13.12 | 160 (69.57%) | 165 (1.54%) | 5.5 | 8.82E-206 | 2.10E-203 | Altered group |
| CCER2 | 19q13.2 | 113 (49.13%) | 117 (1.09%) | 5.49 | 2.74E-137 | 4.08E-135 | Altered group |
| LGALS7 | 19q13.13 | 115 (50.00%) | 121 (1.13%) | 5.47 | 1.87E-139 | 2.83E-137 | Altered group |
| GARRE1 | 19q13.11 | 172 (74.78%) | 182 (1.70%) | 5.46 | 9.13E-224 | 2.31E-221 | Altered group |
| PEPD | 19q13.11 | 151 (65.65%) | 160 (1.49%) | 5.46 | 1.22E-190 | 2.66E-188 | Altered group |
| IFNL4 | 19q13.2 | 114 (49.57%) | 121 (1.13%) | 5.46 | 8.44E-138 | 1.27E-135 | Altered group |
| ERICH4 | 19q13.2 | 44 (19.13%) | 47 (0.44%) | 5.45 | 1.74E-50 | 1.58E-48 | Altered group |
| PSMD8 | 19q13.2 | 125 (54.35%) | 134 (1.25%) | 5.44 | 1.64E-152 | 2.73E-150 | Altered group |
| MIA | 19q13.2 | 69 (30.00%) | 74 (0.69%) | 5.44 | 5.19E-80 | 5.16E-78 | Altered group |
| TDRD12 | 19q13.11 | 139 (60.43%) | 154 (1.44%) | 5.4 | 5.57E-171 | 1.07E-168 | Altered group |
| WDR62 | 19q13.12 | 197 (85.65%) | 219 (2.04%) | 5.39 | 1.42E-264 | 4.18E-262 | Altered group |
| ZNF420 | 19q13.12 | 149 (64.78%) | 168 (1.57%) | 5.37 | 8.23E-185 | 1.76E-182 | Altered group |
| LGALS7B | 19q13.2 | 115 (50.00%) | 130 (1.21%) | 5.37 | 5.84E-137 | 8.65E-135 | Altered group |
| TTC9B | 19q13.2 | 94 (40.87%) | 106 (0.99%) | 5.37 | 9.61E-110 | 1.08E-107 | Altered group |
| EID2B | 19q13.2 | 111 (48.26%) | 126 (1.17%) | 5.36 | 1.65E-131 | 2.36E-129 | Altered group |
| SPRED3 | 19q13 | 123 (53.48%) | 142 (1.32%) | 5.34 | 5.75E-147 | 9.09E-145 | Altered group |
| LEUTX | 19q13.2 | 105 (45.65%) | 122 (1.14%) | 5.33 | 6.64E-123 | 8.28E-121 | Altered group |
| BLVRB | 19q13.2 | 89 (38.70%) | 103 (0.96%) | 5.33 | 9.56E-103 | 1.03E-100 | Altered group |
| NPHS1 | 19q13.12 | 213 (92.61%) | 248 (2.31%) | 5.32 | 2.27E-293 | 7.75E-291 | Altered group |
| SLC7A10 | 19q13.11 | 146 (63.48%) | 170 (1.58%) | 5.32 | 6.09E-179 | 1.24E-176 | Altered group |
| ZNF781 | 19q13.12 | 132 (57.39%) | 155 (1.44%) | 5.31 | 1.15E-158 | 2.01E-156 | Altered group |
| EID2 | 19q13.2 | 112 (48.70%) | 134 (1.25%) | 5.29 | 5.08E-131 | 7.21E-129 | Altered group |
| ACTMAP | 19q13.2 | 78 (33.91%) | 93 (0.87%) | 5.29 | 1.58E-88 | 1.61E-86 | Altered group |
| SYCN | 19q13.2 | 113 (49.13%) | 136 (1.27%) | 5.28 | 4.03E-132 | 5.82E-130 | Altered group |
| LGALS16 | 19q13.2 | 110 (47.83%) | 132 (1.23%) | 5.28 | 2.59E-128 | 3.53E-126 | Altered group |
| CHST8 | 19q13.11 | 159 (69.13%) | 192 (1.79%) | 5.27 | 1.57E-196 | 3.49E-194 | Altered group |
| KCNK6 | 19q13.2 | 125 (54.35%) | 151 (1.41%) | 5.27 | 6.09E-148 | 9.68E-146 | Altered group |
| EIF3K | 19q13.2 | 121 (52.61%) | 146 (1.36%) | 5.27 | 1.31E-142 | 2.02E-140 | Altered group |
| ZNF793 | 19q13.12 | 138 (60.00%) | 168 (1.57%) | 5.26 | 1.55E-165 | 2.85E-163 | Altered group |
| YIF1B | 19q13.2 | 126 (54.78%) | 153 (1.43%) | 5.26 | 4.30E-149 | 6.89E-147 | Altered group |
| CCNP | 19q13.2 | 95 (41.30%) | 116 (1.08%) | 5.26 | 1.08E-108 | 1.20E-106 | Altered group |
| SERTAD1 | 19q13.2 | 91 (39.57%) | 111 (1.03%) | 5.26 | 9.08E-104 | 9.90E-102 | Altered group |
| MRPS12 | 19q13.2 | 114 (49.57%) | 140 (1.30%) | 5.25 | 1.02E-132 | 1.49E-130 | Altered group |
| ZNF571 | 19q13.12 | 136 (59.13%) | 169 (1.58%) | 5.23 | 7.30E-162 | 1.32E-159 | Altered group |
| ZNF875 | 19q13.12 | 136 (59.13%) | 170 (1.58%) | 5.22 | 1.30E-161 | 2.34E-159 | Altered group |
| SERTAD3 | 19q13.2 | 88 (38.26%) | 110 (1.03%) | 5.22 | 2.05E-99 | 2.16E-97 | Altered group |
| ZNF570 | 19q13.12 | 136 (59.13%) | 173 (1.61%) | 5.2 | 7.28E-161 | 1.30E-158 | Altered group |
| RPS16 | 19q13.2 | 112 (48.70%) | 142 (1.32%) | 5.2 | 5.34E-129 | 7.35E-127 | Altered group |
| PCAT19 | 19q13.2 | 43 (18.70%) | 55 (0.51%) | 5.19 | 5.53E-47 | 4.91E-45 | Altered group |
| MED29 | 19q13.2 | 114 (49.57%) | 147 (1.37%) | 5.18 | 5.61E-131 | 7.91E-129 | Altered group |
| SELENOV | 19q13.2 | 112 (48.70%) | 145 (1.35%) | 5.17 | 2.90E-128 | 3.92E-126 | Altered group |
| DPF1 | 19q13.2 | 123 (53.48%) | 160 (1.49%) | 5.16 | 2.14E-142 | 3.28E-140 | Altered group |
| FBXO17 | 19q13.2 | 115 (50.00%) | 150 (1.40%) | 5.16 | 7.46E-132 | 1.07E-129 | Altered group |
| LRP3 | 19q13.11 | 144 (62.61%) | 189 (1.76%) | 5.15 | 1.23E-170 | 2.33E-168 | Altered group |
| ZNF527 | 19q13.12 | 139 (60.43%) | 183 (1.71%) | 5.15 | 1.62E-163 | 2.95E-161 | Altered group |
| WDR88 | 19q13.11 | 145 (63.04%) | 192 (1.79%) | 5.14 | 1.22E-171 | 2.37E-169 | Altered group |
| SPINT2 | 19q13.2 | 125 (54.35%) | 165 (1.54%) | 5.14 | 1.83E-144 | 2.83E-142 | Altered group |
| IFNL1 | 19q13.2 | 116 (50.43%) | 154 (1.44%) | 5.14 | 1.69E-132 | 2.46E-130 | Altered group |
| FAM98C | 19q13.2 | 123 (53.48%) | 164 (1.53%) | 5.13 | 1.96E-141 | 2.98E-139 | Altered group |
| CLC | 19q13.2 | 106 (46.09%) | 141 (1.31%) | 5.13 | 9.89E-120 | 1.17E-117 | Altered group |
| LGALS14 | 19q13.2 | 106 (46.09%) | 141 (1.31%) | 5.13 | 9.89E-120 | 1.17E-117 | Altered group |
| ZNF573 | 19q13.12 | 130 (56.52%) | 174 (1.62%) | 5.12 | 1.47E-150 | 2.38E-148 | Altered group |
| RAB4B | 19q13.2 | 71 (30.87%) | 96 (0.89%) | 5.11 | 2.17E-77 | 2.12E-75 | Altered group |
| ZNF585A | 19q13.12 | 148 (64.35%) | 202 (1.88%) | 5.1 | 1.83E-174 | 3.66E-172 | Altered group |
| ECH1 | 19q13.2 | 115 (50.00%) | 159 (1.48%) | 5.08 | 1.00E-129 | 1.40E-127 | Altered group |
| ZNF225-AS1 | 19q13.31 | 42 (18.26%) | 58 (0.54%) | 5.08 | 6.98E-45 | 6.09E-43 | Altered group |
| ZNF568 | 19q13.12 | 154 (66.96%) | 214 (1.99%) | 5.07 | 3.67E-182 | 7.71E-180 | Altered group |
| GMFG | 19q13.2 | 115 (50.00%) | 160 (1.49%) | 5.07 | 1.70E-129 | 2.36E-127 | Altered group |
| RINL | 19q13.2 | 115 (50.00%) | 160 (1.49%) | 5.07 | 1.70E-129 | 2.36E-127 | Altered group |
| ZNF230-DT | 19q13.31 | 41 (17.83%) | 57 (0.53%) | 5.07 | 1.01E-43 | 8.64E-42 | Altered group |
| NCCRP1 | 19q13.2 | 114 (49.57%) | 159 (1.48%) | 5.06 | 3.87E-128 | 5.21E-126 | Altered group |
| ZFP30 | 19q13.12 | 132 (57.39%) | 186 (1.73%) | 5.05 | 4.49E-151 | 7.35E-149 | Altered group |
| LGALS13 | 19q13.2 | 113 (49.13%) | 160 (1.49%) | 5.04 | 2.49E-126 | 3.28E-124 | Altered group |
| GPATCH1 | 19q13.11 | 147 (63.91%) | 210 (1.96%) | 5.03 | 6.87E-171 | 1.31E-168 | Altered group |
| SNRPA | 19q13.2 | 76 (33.04%) | 109 (1.02%) | 5.02 | 9.97E-82 | 9.95E-80 | Altered group |
| NFKBIB | 19q13.2 | 114 (49.57%) | 166 (1.55%) | 5 | 1.47E-126 | 1.94E-124 | Altered group |
| ZNF585B | 19q13.12 | 146 (63.48%) | 216 (2.01%) | 4.98 | 7.76E-168 | 1.45E-165 | Altered group |
| TIMM50 | 19q13.2 | 112 (48.70%) | 165 (1.54%) | 4.98 | 1.21E-123 | 1.55E-121 | Altered group |
| CATED |  | 40 (17.39%) | 59 (0.55%) | 4.98 | 6.59E-42 | 5.62E-40 | Altered group |
| SLC7A9 | 19q13.11 | 140 (60.87%) | 209 (1.95%) | 4.97 | 3.08E-159 | 5.40E-157 | Altered group |
| DPY19L3 | 19q13.11 | 139 (60.43%) | 207 (1.93%) | 4.97 | 5.27E-158 | 9.12E-156 | Altered group |
| NUMBL | 19q13.2 | 80 (34.78%) | 119 (1.11%) | 4.97 | 2.27E-85 | 2.28E-83 | Altered group |
| FBXO27 | 19q13.2 | 113 (49.13%) | 169 (1.58%) | 4.96 | 2.48E-124 | 3.22E-122 | Altered group |
| C19ORF47 | 19q13.2 | 93 (40.43%) | 139 (1.30%) | 4.96 | 2.93E-100 | 3.13E-98 | Altered group |
| ERVK3-1 | 19q13.43 | 2 (0.87%) | 3 (0.03%) | 4.96 | 4.21E-03 | 0.0159 | Altered group |
| IFNL2 | 19q13.2 | 113 (49.13%) | 170 (1.58%) | 4.95 | 4.08E-124 | 5.24E-122 | Altered group |
| RHPN2 | 19q13.11 | 142 (61.74%) | 217 (2.02%) | 4.93 | 7.43E-161 | 1.31E-158 | Altered group |
| IFNL3 | 19q13.2 | 113 (49.13%) | 174 (1.62%) | 4.92 | 2.93E-123 | 3.71E-121 | Altered group |
| LGALS4 | 19q13.2 | 115 (50.00%) | 179 (1.67%) | 4.91 | 2.56E-125 | 3.35E-123 | Altered group |
| SIRT2 | 19q13.2 | 114 (49.57%) | 177 (1.65%) | 4.91 | 3.51E-124 | 4.54E-122 | Altered group |
| ZFP36 | 19q13.2 | 113 (49.13%) | 175 (1.63%) | 4.91 | 4.77E-123 | 5.97E-121 | Altered group |
| SEC1P | 19q13.33 | 20 (8.70%) | 31 (0.29%) | 4.91 | 5.22E-21 | 2.90E-19 | Altered group |
| CEP89 | 19q13.11 | 140 (60.87%) | 218 (2.03%) | 4.9 | 2.64E-157 | 4.54E-155 | Altered group |
| TMEM91 | 19q13.2 | 46 (20.00%) | 72 (0.67%) | 4.9 | 2.36E-47 | 2.10E-45 | Altered group |
| ZNF540 | 19q13.13 | 135 (58.70%) | 213 (1.99%) | 4.89 | 4.13E-150 | 6.65E-148 | Altered group |
| PSG8-AS1 | 19q13.2 | 40 (17.39%) | 63 (0.59%) | 4.89 | 4.57E-41 | 3.87E-39 | Altered group |
| NUCB1-AS1 | 19q13.33 | 21 (9.13%) | 33 (0.31%) | 4.89 | 6.49E-22 | 3.66E-20 | Altered group |
| ZNF569 | 19q13.12 | 136 (59.13%) | 215 (2.00%) | 4.88 | 2.49E-151 | 4.09E-149 | Altered group |
| FBL | 19q13.2 | 105 (45.65%) | 168 (1.57%) | 4.87 | 3.16E-112 | 3.59E-110 | Altered group |
| LIPE-AS1 | 19q13.2 | 44 (19.13%) | 70 (0.65%) | 4.87 | 4.80E-45 | 4.20E-43 | Altered group |
| ZNF607 | 19q13.1 | 132 (57.39%) | 216 (2.01%) | 4.83 | 1.27E-144 | 1.99E-142 | Altered group |
| GGN | 19q13.2 | 124 (53.91%) | 205 (1.91%) | 4.82 | 4.38E-134 | 6.44E-132 | Altered group |
| EGLN2 | 19q13.2 | 69 (30.00%) | 114 (1.06%) | 4.82 | 1.17E-70 | 1.11E-68 | Altered group |
| BLOC1S3 | 19q13.32 | 39 (16.96%) | 66 (0.62%) | 4.78 | 3.93E-39 | 3.25E-37 | Altered group |
| SARS2 | 19q13.2 | 113 (49.13%) | 193 (1.80%) | 4.77 | 2.14E-119 | 2.51E-117 | Altered group |
| B9D2 | 19q13.2 | 47 (20.43%) | 81 (0.75%) | 4.76 | 6.37E-47 | 5.64E-45 | Altered group |
| DMAC2 | 19q13.2 | 44 (19.13%) | 76 (0.71%) | 4.76 | 7.25E-44 | 6.25E-42 | Altered group |
| PSG10P | 19q13.2 | 40 (17.39%) | 69 (0.64%) | 4.76 | 6.96E-40 | 5.79E-38 | Altered group |
| PSMC4 | 19q13.11-q13.13 | 102 (44.35%) | 177 (1.65%) | 4.75 | 6.14E-106 | 6.78E-104 | Altered group |
| EML2-AS1 | 19q13.32 | 34 (14.78%) | 59 (0.55%) | 4.75 | 7.12E-34 | 5.20E-32 | Altered group |
| PAK4 | 19q13.2 | 117 (50.87%) | 205 (1.91%) | 4.73 | 3.36E-123 | 4.23E-121 | Altered group |
| DLL3 | 19q13.2 | 113 (49.13%) | 198 (1.85%) | 4.73 | 1.97E-118 | 2.27E-116 | Altered group |
| EXOSC5 | 19q13.2 | 45 (19.57%) | 79 (0.74%) | 4.73 | 1.25E-44 | 1.08E-42 | Altered group |
| RASGRP4 | 19q13.2 | 122 (53.04%) | 216 (2.01%) | 4.72 | 8.20E-129 | 1.12E-126 | Altered group |
| TGFB1 | 19q13.2 | 51 (22.17%) | 90 (0.84%) | 4.72 | 1.39E-50 | 1.26E-48 | Altered group |
| PINLYP | 19q13.31 | 39 (16.96%) | 69 (0.64%) | 4.72 | 1.45E-38 | 1.19E-36 | Altered group |
| RABAC1 | 19q13.2 | 39 (16.96%) | 69 (0.64%) | 4.72 | 1.45E-38 | 1.19E-36 | Altered group |
| CXCL17 | 19q13.2 | 42 (18.26%) | 75 (0.70%) | 4.71 | 2.11E-41 | 1.79E-39 | Altered group |
| CNFN | 19q13.2 | 41 (17.83%) | 73 (0.68%) | 4.71 | 1.87E-40 | 1.57E-38 | Altered group |
| PAF1 | 19q13.2 | 114 (49.57%) | 206 (1.92%) | 4.69 | 1.93E-118 | 2.24E-116 | Altered group |
| ACP7 | 19q13.2 | 115 (50.00%) | 210 (1.96%) | 4.68 | 3.26E-119 | 3.82E-117 | Altered group |
| COQ8B | 19q13.2 | 75 (32.61%) | 137 (1.28%) | 4.67 | 1.17E-74 | 1.12E-72 | Altered group |
| ANKRD27 | 19q13.11 | 143 (62.17%) | 264 (2.46%) | 4.66 | 3.56E-153 | 5.95E-151 | Altered group |
| ZNF507 | 19q13.11 | 137 (59.57%) | 252 (2.35%) | 4.66 | 1.07E-145 | 1.67E-143 | Altered group |
| CAPN12 | 19q13.2 | 118 (51.30%) | 218 (2.03%) | 4.66 | 2.69E-122 | 3.31E-120 | Altered group |
| PLD3 | 19q13.2 | 92 (40.00%) | 171 (1.59%) | 4.65 | 1.81E-92 | 1.87E-90 | Altered group |
| PAFAH1B3 | 19q13.1 | 41 (17.83%) | 76 (0.71%) | 4.65 | 6.57E-40 | 5.49E-38 | Altered group |
| MEIOSIN | 19q13.32 | 35 (15.22%) | 65 (0.61%) | 4.65 | 4.47E-34 | 3.30E-32 | Altered group |
| LRFN1 | 19q13.2 | 117 (50.87%) | 219 (2.04%) | 4.64 | 1.35E-120 | 1.62E-118 | Altered group |
| SAMD4B | 19q13.2 | 117 (50.87%) | 219 (2.04%) | 4.64 | 1.35E-120 | 1.62E-118 | Altered group |
| RPS19 | 19q13.2 | 38 (16.52%) | 71 (0.66%) | 4.64 | 6.79E-37 | 5.42E-35 | Altered group |
| DYRK1B | 19q13.2 | 110 (47.83%) | 208 (1.94%) | 4.62 | 4.49E-112 | 5.08E-110 | Altered group |
| B3GNT8 | 19q13.2 | 46 (20.00%) | 87 (0.81%) | 4.62 | 1.69E-44 | 1.47E-42 | Altered group |
| AKT2 | 19q13.2 | 95 (41.30%) | 181 (1.69%) | 4.61 | 5.56E-95 | 5.82E-93 | Altered group |
| HNRNPL | 19q13.2 | 116 (50.43%) | 224 (2.09%) | 4.59 | 3.40E-118 | 3.90E-116 | Altered group |
| ITPKC | 19q13.2 | 78 (33.91%) | 151 (1.41%) | 4.59 | 2.56E-76 | 2.49E-74 | Altered group |
| HIPK4 | 19q13.2 | 92 (40.00%) | 180 (1.68%) | 4.58 | 7.09E-91 | 7.26E-89 | Altered group |
| SAXO3 | 19q13.33 | 18 (7.83%) | 35 (0.33%) | 4.58 | 1.08E-17 | 5.29E-16 | Altered group |
| CCDC97 | 19q13.2 | 50 (21.74%) | 98 (0.91%) | 4.57 | 7.91E-48 | 7.08E-46 | Altered group |
| KMT2B | 19q13.12 | 224 (97.39%) | 443 (4.13%) | 4.56 | 2.49E-279 | 7.92E-277 | Altered group |
| ZNF575 | 19q13.31 | 40 (17.39%) | 79 (0.74%) | 4.56 | 4.33E-38 | 3.54E-36 | Altered group |
| CEACAM22P | 19q13.31 | 39 (16.96%) | 77 (0.72%) | 4.56 | 3.82E-37 | 3.06E-35 | Altered group |
| SHKBP1 | 19q13.2 | 86 (37.39%) | 173 (1.61%) | 4.54 | 1.22E-83 | 1.22E-81 | Altered group |
| CARD8-AS1 | 19q13.33 | 19 (8.26%) | 38 (0.35%) | 4.54 | 1.95E-18 | 1.00E-16 | Altered group |
| CGB3 | 19q13.33 | 18 (7.83%) | 36 (0.34%) | 4.54 | 1.59E-17 | 7.72E-16 | Altered group |
| SNAR-G1 | 19q13.33 | 18 (7.83%) | 36 (0.34%) | 4.54 | 1.59E-17 | 7.72E-16 | Altered group |
| SNAR-G2 | 19q13.33 | 18 (7.83%) | 36 (0.34%) | 4.54 | 1.59E-17 | 7.72E-16 | Altered group |
| CATSPERG | 19q13.2 | 125 (54.35%) | 253 (2.36%) | 4.53 | 8.75E-127 | 1.16E-124 | Altered group |
| MAP4K1 | 19q13.2 | 120 (52.17%) | 242 (2.26%) | 4.53 | 4.33E-121 | 5.28E-119 | Altered group |
| LINC01532 | 19q12 | 106 (46.09%) | 215 (2.00%) | 4.52 | 5.58E-105 | 6.13E-103 | Altered group |
| LINC02987 | 19q11 | 75 (32.61%) | 154 (1.44%) | 4.51 | 1.06E-71 | 1.01E-69 | Altered group |
| APOC1P1 | 19q13.32 | 39 (16.96%) | 80 (0.75%) | 4.51 | 1.21E-36 | 9.61E-35 | Altered group |
| VSTM2B-DT | 19q12 | 121 (52.61%) | 250 (2.33%) | 4.5 | 3.03E-121 | 3.72E-119 | Altered group |
| LINC00906 | 19q12 | 102 (44.35%) | 210 (1.96%) | 4.5 | 4.80E-100 | 5.09E-98 | Altered group |
| LINC00662 | 19q11 | 75 (32.61%) | 155 (1.44%) | 4.5 | 1.55E-71 | 1.47E-69 | Altered group |
| CYP2F1 | 19q13.2 | 60 (26.09%) | 124 (1.16%) | 4.5 | 1.33E-56 | 1.22E-54 | Altered group |
| SNAR-A1 | 19q13.33 | 17 (7.39%) | 35 (0.33%) | 4.5 | 1.88E-16 | 8.29E-15 | Altered group |
| SNAR-A12 | 19q13.33 | 17 (7.39%) | 35 (0.33%) | 4.5 | 1.88E-16 | 8.29E-15 | Altered group |
| SNAR-C1 | 19q13.32 | 17 (7.39%) | 35 (0.33%) | 4.5 | 1.88E-16 | 8.29E-15 | Altered group |
| SNAR-C2 | 19q13.32 | 17 (7.39%) | 35 (0.33%) | 4.5 | 1.88E-16 | 8.29E-15 | Altered group |
| SNAR-C3 | 19q13.32 | 17 (7.39%) | 35 (0.33%) | 4.5 | 1.88E-16 | 8.29E-15 | Altered group |
| SNAR-C4 | 19q13.32 | 17 (7.39%) | 35 (0.33%) | 4.5 | 1.88E-16 | 8.29E-15 | Altered group |
| NAPA-AS1 | 19q13.32-q13.33 | 16 (6.96%) | 33 (0.31%) | 4.5 | 1.52E-15 | 6.13E-14 | Altered group |
| APOC4 | 19q13.32 | 39 (16.96%) | 81 (0.75%) | 4.49 | 1.76E-36 | 1.39E-34 | Altered group |
| DACT3-AS1 | 19q13.32 | 20 (8.70%) | 43 (0.40%) | 4.44 | 7.27E-19 | 3.83E-17 | Altered group |
| WDR87 | 19q13.13 | 128 (55.65%) | 278 (2.59%) | 4.42 | 3.24E-127 | 4.33E-125 | Altered group |
| ACTN4 | 19q13.2 | 125 (54.35%) | 273 (2.54%) | 4.42 | 1.70E-123 | 2.16E-121 | Altered group |
| SNAR-E | 19q13.32 | 21 (9.13%) | 46 (0.43%) | 4.41 | 1.27E-19 | 6.92E-18 | Altered group |
| LOC93429 | 19q13.32 | 24 (10.43%) | 53 (0.49%) | 4.4 | 3.27E-22 | 1.86E-20 | Altered group |
| PTOV1-AS1 | 19q13.33 | 19 (8.26%) | 42 (0.39%) | 4.4 | 8.44E-18 | 4.17E-16 | Altered group |
| MAP3K10 | 19q13.2 | 97 (42.17%) | 217 (2.02%) | 4.38 | 7.50E-92 | 7.71E-90 | Altered group |
| CYP2S1 | 19q13.2 | 55 (23.91%) | 123 (1.15%) | 4.38 | 2.90E-50 | 2.61E-48 | Altered group |
| APOC2 | 19q13.32 | 41 (17.83%) | 92 (0.86%) | 4.38 | 2.84E-37 | 2.28E-35 | Altered group |
| SNORD23 | 19q13.32 | 17 (7.39%) | 38 (0.35%) | 4.38 | 5.53E-16 | 2.30E-14 | Altered group |
| ZNF780A | 19q13.2 | 101 (43.91%) | 228 (2.12%) | 4.37 | 9.52E-96 | 1.00E-93 | Altered group |
| ZNF473CR | 19q13.33 | 16 (6.96%) | 36 (0.34%) | 4.37 | 4.45E-15 | 1.71E-13 | Altered group |
| BCKDHA | 19q13.2 | 48 (20.87%) | 109 (1.02%) | 4.36 | 1.72E-43 | 1.47E-41 | Altered group |
| TRAPPC6A | 19q13.32 | 40 (17.39%) | 91 (0.85%) | 4.36 | 3.54E-36 | 2.76E-34 | Altered group |
| IGSF23 | 19q13.31 | 42 (18.26%) | 96 (0.89%) | 4.35 | 6.42E-38 | 5.21E-36 | Altered group |
| ZNF428 | 19q13.31 | 39 (16.96%) | 89 (0.83%) | 4.35 | 3.10E-35 | 2.37E-33 | Altered group |
| GNG8 | 19q13.32 | 21 (9.13%) | 48 (0.45%) | 4.35 | 2.55E-19 | 1.38E-17 | Altered group |
| CD177 | 19q13.31 | 40 (17.39%) | 93 (0.87%) | 4.33 | 7.01E-36 | 5.40E-34 | Altered group |
| SNORD88A | 19q13.33 | 19 (8.26%) | 44 (0.41%) | 4.33 | 1.68E-17 | 8.14E-16 | Altered group |
| SNORD88B | 19q13.33 | 19 (8.26%) | 44 (0.41%) | 4.33 | 1.68E-17 | 8.14E-16 | Altered group |
| PLEKHF1 | 19q12 | 127 (55.22%) | 297 (2.77%) | 4.32 | 9.00E-123 | 1.12E-120 | Altered group |
| UQCRFS1 | 19q12 | 112 (48.70%) | 262 (2.44%) | 4.32 | 3.62E-106 | 4.02E-104 | Altered group |
| CYP2A6 | 19q13.2 | 69 (30.00%) | 161 (1.50%) | 4.32 | 1.25E-62 | 1.17E-60 | Altered group |
| CYP2A7 | 19q13.2 | 67 (29.13%) | 156 (1.45%) | 4.32 | 9.07E-61 | 8.44E-59 | Altered group |
| CEACAM20 | 19q13.31 | 39 (16.96%) | 91 (0.85%) | 4.32 | 6.13E-35 | 4.63E-33 | Altered group |
| RNU6-66P | 13q22.1 | 24 (10.43%) | 56 (0.52%) | 4.32 | 9.18E-22 | 5.17E-20 | Altered group |
| POP4 | 19q12 | 125 (54.35%) | 294 (2.74%) | 4.31 | 2.93E-120 | 3.50E-118 | Altered group |
| CEACAM21 | 19q13.2 | 46 (20.00%) | 109 (1.02%) | 4.3 | 5.50E-41 | 4.64E-39 | Altered group |
| SNORD88C | 19q13.33 | 19 (8.26%) | 45 (0.42%) | 4.3 | 2.34E-17 | 1.12E-15 | Altered group |
| C19ORF12 | 19q12 | 126 (54.78%) | 300 (2.80%) | 4.29 | 7.62E-121 | 9.25E-119 | Altered group |
| ZNF546 | 19q13.2 | 101 (43.91%) | 241 (2.25%) | 4.29 | 8.50E-94 | 8.86E-92 | Altered group |
| DEDD2 | 19q13.2 | 42 (18.26%) | 100 (0.93%) | 4.29 | 2.48E-37 | 2.00E-35 | Altered group |
| APOC1 | 19q13.32 | 41 (17.83%) | 98 (0.91%) | 4.29 | 2.18E-36 | 1.71E-34 | Altered group |
| CD79A | 19q13.2 | 39 (16.96%) | 93 (0.87%) | 4.29 | 1.20E-34 | 8.98E-33 | Altered group |
| SNRPD2 | 19q13.2-q13.3 | 37 (16.09%) | 88 (0.82%) | 4.29 | 6.45E-33 | 4.58E-31 | Altered group |
| VSTM2B | 19q12 | 121 (52.61%) | 290 (2.70%) | 4.28 | 5.42E-115 | 6.19E-113 | Altered group |
| ZNF780B | 19q13.2 | 100 (43.48%) | 241 (2.25%) | 4.27 | 2.05E-92 | 2.12E-90 | Altered group |
| TMEM277P | 19q13.33 | 19 (8.26%) | 46 (0.43%) | 4.27 | 3.25E-17 | 1.53E-15 | Altered group |
| PPP1R37 | 19q13.32 | 40 (17.39%) | 98 (0.91%) | 4.25 | 3.67E-35 | 2.80E-33 | Altered group |
| LYPD5 | 19q13.31 | 40 (17.39%) | 99 (0.92%) | 4.24 | 5.07E-35 | 3.85E-33 | Altered group |
| PTOV1-AS2 | 19q13.33 | 17 (7.39%) | 42 (0.39%) | 4.24 | 2.09E-15 | 8.30E-14 | Altered group |
| ETHE1 | 19q13.31 | 40 (17.39%) | 100 (0.93%) | 4.22 | 6.97E-35 | 5.24E-33 | Altered group |
| ZNF350-AS1 | 19q13.41 | 24 (10.43%) | 60 (0.56%) | 4.22 | 3.37E-21 | 1.88E-19 | Altered group |
| ZNF649-AS1 | 19q13.41 | 24 (10.43%) | 60 (0.56%) | 4.22 | 3.37E-21 | 1.88E-19 | Altered group |
| SIGLEC10-AS1 | 19q13.41 | 20 (8.70%) | 50 (0.47%) | 4.22 | 7.67E-18 | 3.81E-16 | Altered group |
| INAFM1 | 19q13.32 | 14 (6.09%) | 35 (0.33%) | 4.22 | 7.62E-13 | 2.46E-11 | Altered group |
| APOC4-APOC2 | 19q13.32 | 39 (16.96%) | 98 (0.91%) | 4.21 | 6.04E-34 | 4.44E-32 | Altered group |
| LYPD4 | 19q13.2 | 39 (16.96%) | 100 (0.93%) | 4.19 | 1.13E-33 | 8.21E-32 | Altered group |
| CGB8 | 19q13.33 | 18 (7.83%) | 46 (0.43%) | 4.19 | 4.90E-16 | 2.05E-14 | Altered group |
| SIGLEC16 | 19q13.33 | 16 (6.96%) | 41 (0.38%) | 4.19 | 2.27E-14 | 8.23E-13 | Altered group |
| CEACAM7 | 19q13.2 | 45 (19.57%) | 116 (1.08%) | 4.18 | 8.87E-39 | 7.33E-37 | Altered group |
| CEACAM4 | 19q13.2 | 44 (19.13%) | 113 (1.05%) | 4.18 | 5.74E-38 | 4.67E-36 | Altered group |
| TOMM40 | 19q13.32 | 42 (18.26%) | 109 (1.02%) | 4.17 | 4.40E-36 | 3.41E-34 | Altered group |
| GEMIN7 | 19q13.32 | 39 (16.96%) | 101 (0.94%) | 4.17 | 1.54E-33 | 1.12E-31 | Altered group |
| OPA3 | 19q13.32 | 36 (15.65%) | 93 (0.87%) | 4.17 | 5.18E-31 | 3.48E-29 | Altered group |
| SNAR-B1 | 19q13.33 | 17 (7.39%) | 44 (0.41%) | 4.17 | 3.89E-15 | 1.50E-13 | Altered group |
| SNAR-D | 19q13.33 | 17 (7.39%) | 44 (0.41%) | 4.17 | 3.89E-15 | 1.50E-13 | Altered group |
| PLEKHG2 | 19q13.2 | 112 (48.70%) | 295 (2.75%) | 4.15 | 1.74E-101 | 1.86E-99 | Altered group |
| APOE | 19q13.32 | 41 (17.83%) | 108 (1.01%) | 4.15 | 5.18E-35 | 3.92E-33 | Altered group |
| ZNF576 | 19q13.31 | 38 (16.52%) | 100 (0.93%) | 4.15 | 1.79E-32 | 1.24E-30 | Altered group |
| SNAR-F | 19q13.33 | 16 (6.96%) | 42 (0.39%) | 4.15 | 3.07E-14 | 1.11E-12 | Altered group |
| GSK3A | 19q13.2 | 43 (18.70%) | 114 (1.06%) | 4.14 | 1.26E-36 | 9.95E-35 | Altered group |
| TEX101 | 19q13.31 | 43 (18.70%) | 114 (1.06%) | 4.14 | 1.26E-36 | 9.95E-35 | Altered group |
| RPL18 | 19q13.33 | 20 (8.70%) | 53 (0.49%) | 4.14 | 1.93E-17 | 9.29E-16 | Altered group |
| SUPT5H | 19q13.2 | 115 (50.00%) | 306 (2.85%) | 4.13 | 3.41E-104 | 3.74E-102 | Altered group |
| IGFL4 | 19q13.32 | 27 (11.74%) | 72 (0.67%) | 4.13 | 3.94E-23 | 2.26E-21 | Altered group |
| KLKP1 | 19q13.33 | 20 (8.70%) | 54 (0.50%) | 4.11 | 2.59E-17 | 1.23E-15 | Altered group |
| SIGLEC17P | 19q13.41 | 20 (8.70%) | 54 (0.50%) | 4.11 | 2.59E-17 | 1.23E-15 | Altered group |
| PPM1N | 19q13.32 | 36 (15.65%) | 98 (0.91%) | 4.1 | 2.33E-30 | 1.52E-28 | Altered group |
| SNAR-A10 | 19q13.33 | 22 (9.57%) | 60 (0.56%) | 4.1 | 7.38E-19 | 3.83E-17 | Altered group |
| SNAR-A11 | 19q13.33 | 22 (9.57%) | 60 (0.56%) | 4.1 | 7.38E-19 | 3.83E-17 | Altered group |
| SNAR-A14 | 19q13.33 | 22 (9.57%) | 60 (0.56%) | 4.1 | 7.38E-19 | 3.83E-17 | Altered group |
| SNAR-A3 | 19q13.33 | 22 (9.57%) | 60 (0.56%) | 4.1 | 7.38E-19 | 3.83E-17 | Altered group |
| SNAR-A4 | 19q13.33 | 22 (9.57%) | 60 (0.56%) | 4.1 | 7.38E-19 | 3.83E-17 | Altered group |
| SNAR-A5 | 19q13.33 | 22 (9.57%) | 60 (0.56%) | 4.1 | 7.38E-19 | 3.83E-17 | Altered group |
| SNAR-A6 | 19q13.33 | 22 (9.57%) | 60 (0.56%) | 4.1 | 7.38E-19 | 3.83E-17 | Altered group |
| SNAR-A7 | 19q13.33 | 22 (9.57%) | 60 (0.56%) | 4.1 | 7.38E-19 | 3.83E-17 | Altered group |
| SNAR-A8 | 19q13.33 | 22 (9.57%) | 60 (0.56%) | 4.1 | 7.38E-19 | 3.83E-17 | Altered group |
| SPACA6 | 19q13.41 | 21 (9.13%) | 57 (0.53%) | 4.1 | 4.38E-18 | 2.20E-16 | Altered group |
| LYPD3 | 19q13.31 | 43 (18.70%) | 118 (1.10%) | 4.09 | 4.13E-36 | 3.21E-34 | Altered group |
| ZNF702P | 19q13.41 | 23 (10.00%) | 63 (0.59%) | 4.09 | 1.24E-19 | 6.75E-18 | Altered group |
| ZNF816-ZNF321P | 19q13.41 | 23 (10.00%) | 63 (0.59%) | 4.09 | 1.24E-19 | 6.75E-18 | Altered group |
| GFY | 19q13.33 | 19 (8.26%) | 52 (0.48%) | 4.09 | 2.05E-16 | 8.99E-15 | Altered group |
| NKPD1 | 19q13.32 | 41 (17.83%) | 113 (1.05%) | 4.08 | 2.29E-34 | 1.71E-32 | Altered group |
| C19ORF84 | 19q13.41 | 21 (9.13%) | 58 (0.54%) | 4.08 | 5.86E-18 | 2.92E-16 | Altered group |
| C19ORF81 | 19q13.33 | 16 (6.96%) | 44 (0.41%) | 4.08 | 5.54E-14 | 1.97E-12 | Altered group |
| ZNF137P | 19q13.41 | 22 (9.57%) | 61 (0.57%) | 4.07 | 9.86E-19 | 5.10E-17 | Altered group |
| NANOS2 | 19q13.32 | 31 (13.48%) | 87 (0.81%) | 4.06 | 6.85E-26 | 4.05E-24 | Altered group |
| RPL13AP5 | 10q24.1 | 19 (8.26%) | 53 (0.49%) | 4.06 | 2.73E-16 | 1.18E-14 | Altered group |
| SNORD32A | 19q13.33 | 19 (8.26%) | 53 (0.49%) | 4.06 | 2.73E-16 | 1.18E-14 | Altered group |
| SNORD33 | 19q13.33 | 19 (8.26%) | 53 (0.49%) | 4.06 | 2.73E-16 | 1.18E-14 | Altered group |
| SNORD34 | 19q13.33 | 19 (8.26%) | 53 (0.49%) | 4.06 | 2.73E-16 | 1.18E-14 | Altered group |
| SNORD35A | 19q13.33 | 19 (8.26%) | 53 (0.49%) | 4.06 | 2.73E-16 | 1.18E-14 | Altered group |
| SNORD35B | 19q13.33 | 19 (8.26%) | 53 (0.49%) | 4.06 | 2.73E-16 | 1.18E-14 | Altered group |
| CYP2B6 | 19q13.2 | 67 (29.13%) | 189 (1.76%) | 4.05 | 2.77E-56 | 2.54E-54 | Altered group |
| ADM5 | 19q13.33 | 21 (9.13%) | 59 (0.55%) | 4.05 | 7.79E-18 | 3.86E-16 | Altered group |
| DMRTC2 | 19q13.2 | 39 (16.96%) | 111 (1.03%) | 4.03 | 2.94E-32 | 2.02E-30 | Altered group |
| SPACA6-AS1 | 19q13.41 | 20 (8.70%) | 57 (0.53%) | 4.03 | 6.13E-17 | 2.85E-15 | Altered group |
| NAPSB | 19q13.33 | 16 (6.96%) | 46 (0.43%) | 4.02 | 9.75E-14 | 3.40E-12 | Altered group |
| TMEM160 | 19q13.32 | 15 (6.52%) | 43 (0.40%) | 4.02 | 5.71E-13 | 1.87E-11 | Altered group |
| CYP2A13 | 19q13.2 | 59 (25.65%) | 171 (1.59%) | 4.01 | 1.01E-48 | 9.11E-47 | Altered group |
| SELENOW | 19q13.33 | 18 (7.83%) | 52 (0.48%) | 4.01 | 2.82E-15 | 1.12E-13 | Altered group |
| ZNF526 | 19q13.2 | 43 (18.70%) | 125 (1.16%) | 4 | 3.04E-35 | 2.33E-33 | Altered group |
| ZNF296 | 19q13.32 | 40 (17.39%) | 117 (1.09%) | 4 | 1.06E-32 | 7.46E-31 | Altered group |
| CCNE1 | 19q12 | 125 (54.35%) | 368 (3.43%) | 3.99 | 2.89E-110 | 3.25E-108 | Altered group |
| PRR19 | 19q13.2 | 42 (18.26%) | 123 (1.15%) | 3.99 | 2.59E-34 | 1.92E-32 | Altered group |
| MYPOP | 19q13.32 | 35 (15.22%) | 103 (0.96%) | 3.99 | 1.41E-28 | 8.92E-27 | Altered group |
| SIPA1L3 | 19q13.13-q13.2 | 133 (57.83%) | 393 (3.66%) | 3.98 | 1.52E-118 | 1.77E-116 | Altered group |
| CEACAM3 | 19q13.2 | 40 (17.39%) | 118 (1.10%) | 3.98 | 1.40E-32 | 9.76E-31 | Altered group |
| CLDND2 | 19q13.41 | 21 (9.13%) | 62 (0.58%) | 3.98 | 1.79E-17 | 8.66E-16 | Altered group |
| AP2S1 | 19q13.32 | 20 (8.70%) | 59 (0.55%) | 3.98 | 1.06E-16 | 4.84E-15 | Altered group |
| SPACA4 | 19q13.33 | 20 (8.70%) | 59 (0.55%) | 3.98 | 1.06E-16 | 4.84E-15 | Altered group |
| LINC01530 | 19q13.41 | 19 (8.26%) | 56 (0.52%) | 3.98 | 6.29E-16 | 2.61E-14 | Altered group |
| SRRM5 | 19q13.31 | 39 (16.96%) | 116 (1.08%) | 3.97 | 1.17E-31 | 8.00E-30 | Altered group |
| HAVCR1P1 | 19p12 | 71 (30.87%) | 213 (1.99%) | 3.96 | 2.71E-58 | 2.52E-56 | Altered group |
| PVR | 19q13.31 | 45 (19.57%) | 135 (1.26%) | 3.96 | 2.15E-36 | 1.69E-34 | Altered group |
| ZSWIM9 | 19q13.33 | 20 (8.70%) | 60 (0.56%) | 3.96 | 1.39E-16 | 6.29E-15 | Altered group |
| LTBP4 | 19q13.2 | 90 (39.13%) | 271 (2.53%) | 3.95 | 3.46E-75 | 3.32E-73 | Altered group |
| IRGQ | 19q13.31 | 41 (17.83%) | 124 (1.16%) | 3.95 | 4.93E-33 | 3.53E-31 | Altered group |
| CEACAM19 | 19q13.31 | 42 (18.26%) | 128 (1.19%) | 3.94 | 1.00E-33 | 7.30E-32 | Altered group |
| SMG9 | 19q13.31 | 41 (17.83%) | 125 (1.16%) | 3.94 | 6.43E-33 | 4.58E-31 | Altered group |
| CEACAM6 | 19q13.2 | 39 (16.96%) | 119 (1.11%) | 3.93 | 2.62E-31 | 1.77E-29 | Altered group |
| PRX | 19q13.2 | 92 (40.00%) | 284 (2.65%) | 3.92 | 3.26E-76 | 3.16E-74 | Altered group |
| CEACAM16 | 19q13.31-q13.32 | 45 (19.57%) | 139 (1.30%) | 3.92 | 6.23E-36 | 4.81E-34 | Altered group |
| KLC3 | 19q13.32 | 39 (16.96%) | 121 (1.13%) | 3.91 | 4.44E-31 | 2.99E-29 | Altered group |
| C19ORF73 | 19q13.33 | 18 (7.83%) | 56 (0.52%) | 3.91 | 8.21E-15 | 3.07E-13 | Altered group |
| CEACAM1 | 19q13.2 | 41 (17.83%) | 128 (1.19%) | 3.9 | 1.41E-32 | 9.82E-31 | Altered group |
| IRF2BP1 | 19q13.32 | 38 (16.52%) | 119 (1.11%) | 3.9 | 3.65E-30 | 2.37E-28 | Altered group |
| IGFL3 | 19q13.32 | 26 (11.30%) | 81 (0.75%) | 3.9 | 6.69E-21 | 3.71E-19 | Altered group |
| PGLYRP1 | 19q13.32 | 29 (12.61%) | 91 (0.85%) | 3.89 | 3.87E-23 | 2.22E-21 | Altered group |
| RNU6-35P | 4q25 | 27 (11.74%) | 85 (0.79%) | 3.89 | 1.43E-21 | 8.02E-20 | Altered group |
| RNU6-64P | 13q12.3 | 27 (11.74%) | 85 (0.79%) | 3.89 | 1.43E-21 | 8.02E-20 | Altered group |
| CALM3 | 19q13.32 | 21 (9.13%) | 66 (0.62%) | 3.89 | 5.14E-17 | 2.40E-15 | Altered group |
| CADM4 | 19q13.31 | 40 (17.39%) | 127 (1.18%) | 3.88 | 1.51E-31 | 1.03E-29 | Altered group |
| IGFL1 | 19q13.32 | 24 (10.43%) | 76 (0.71%) | 3.88 | 3.13E-19 | 1.67E-17 | Altered group |
| CGB5 | 19q13.33 | 19 (8.26%) | 60 (0.56%) | 3.88 | 1.80E-15 | 7.19E-14 | Altered group |
| CGB2 | 19q13.33 | 18 (7.83%) | 57 (0.53%) | 3.88 | 1.06E-14 | 3.93E-13 | Altered group |
| URI1 | 19q12 | 120 (52.17%) | 383 (3.57%) | 3.87 | 1.33E-101 | 1.43E-99 | Altered group |
| VASP | 19q13.32 | 36 (15.65%) | 115 (1.07%) | 3.87 | 2.41E-28 | 1.51E-26 | Altered group |
| IGFL2 | 19q13.32 | 27 (11.74%) | 86 (0.80%) | 3.87 | 1.85E-21 | 1.03E-19 | Altered group |
| NTN5 | 19q13.33 | 21 (9.13%) | 67 (0.62%) | 3.87 | 6.63E-17 | 3.07E-15 | Altered group |
| PLAUR | 19q13 | 39 (16.96%) | 125 (1.16%) | 3.86 | 1.24E-30 | 8.21E-29 | Altered group |
| CTU1 | 19q13.41 | 19 (8.26%) | 61 (0.57%) | 3.86 | 2.32E-15 | 9.20E-14 | Altered group |
| LHB | 19q13.33 | 18 (7.83%) | 58 (0.54%) | 3.86 | 1.36E-14 | 5.01E-13 | Altered group |
| POLR1G | 19q13.32 | 39 (16.96%) | 126 (1.17%) | 3.85 | 1.60E-30 | 1.05E-28 | Altered group |
| LILRP2 | 19q13.42 | 26 (11.30%) | 84 (0.78%) | 3.85 | 1.43E-20 | 7.91E-19 | Altered group |
| ZNF726 | 19p12 | 72 (31.30%) | 234 (2.18%) | 3.84 | 4.10E-57 | 3.79E-55 | Altered group |
| ZNF222 | 19q13.31 | 42 (18.26%) | 137 (1.28%) | 3.84 | 1.01E-32 | 7.13E-31 | Altered group |
| CCDC61 | 19q13.32 | 32 (13.91%) | 104 (0.97%) | 3.84 | 4.63E-25 | 2.70E-23 | Altered group |
| C19ORF48P | 19q13.33 | 19 (8.26%) | 62 (0.58%) | 3.84 | 2.97E-15 | 1.17E-13 | Altered group |
| PTH2 | 19q13.33 | 19 (8.26%) | 62 (0.58%) | 3.84 | 2.97E-15 | 1.17E-13 | Altered group |
| POU2F2 | 19q13.2 | 43 (18.70%) | 141 (1.31%) | 3.83 | 2.02E-33 | 1.46E-31 | Altered group |
| QPCTL | 19q13.32 | 35 (15.22%) | 115 (1.07%) | 3.83 | 3.20E-27 | 1.95E-25 | Altered group |
| LENG9 | 19q13.42 | 25 (10.87%) | 82 (0.76%) | 3.83 | 1.11E-19 | 6.05E-18 | Altered group |
| ZNF45 | 19q13.31 | 44 (19.13%) | 145 (1.35%) | 3.82 | 4.01E-34 | 2.97E-32 | Altered group |
| RELB | 19q13.32 | 43 (18.70%) | 142 (1.32%) | 3.82 | 2.59E-33 | 1.87E-31 | Altered group |
| ZNF155 | 19q13.31 | 42 (18.26%) | 139 (1.30%) | 3.82 | 1.67E-32 | 1.15E-30 | Altered group |
| FOSB | 19q13.32 | 36 (15.65%) | 119 (1.11%) | 3.82 | 6.56E-28 | 4.06E-26 | Altered group |
| GIPR | 19q13.32 | 35 (15.22%) | 116 (1.08%) | 3.82 | 4.09E-27 | 2.48E-25 | Altered group |
| DACT3 | 19q13.32 | 20 (8.70%) | 66 (0.62%) | 3.82 | 6.44E-16 | 2.67E-14 | Altered group |
| ERVV-2 | 19q13.41 | 21 (9.13%) | 70 (0.65%) | 3.81 | 1.39E-16 | 6.29E-15 | Altered group |
| CGB7 | 19q13.33 | 19 (8.26%) | 63 (0.59%) | 3.81 | 3.80E-15 | 1.48E-13 | Altered group |
| ZNF230 | 19q13.31 | 43 (18.70%) | 144 (1.34%) | 3.8 | 4.23E-33 | 3.04E-31 | Altered group |
| DBP | 19q13.33 | 20 (8.70%) | 67 (0.62%) | 3.8 | 8.22E-16 | 3.38E-14 | Altered group |
| ZNF223 | 19q13.31 | 43 (18.70%) | 145 (1.35%) | 3.79 | 5.39E-33 | 3.85E-31 | Altered group |
| EXOC3L2 | 19q13.32 | 40 (17.39%) | 135 (1.26%) | 3.79 | 1.10E-30 | 7.32E-29 | Altered group |
| ZNF818P | 19q13.42 | 21 (9.13%) | 71 (0.66%) | 3.79 | 1.77E-16 | 7.89E-15 | Altered group |
| BCL3 | 19q13.32 | 45 (19.57%) | 153 (1.43%) | 3.78 | 2.10E-34 | 1.57E-32 | Altered group |
| ERF | 19q13.2 | 43 (18.70%) | 146 (1.36%) | 3.78 | 6.86E-33 | 4.85E-31 | Altered group |
| PHLDB3 | 19q13.31 | 42 (18.26%) | 143 (1.33%) | 3.78 | 4.40E-32 | 3.02E-30 | Altered group |
| NOVA2 | 19q13.32 | 30 (13.04%) | 102 (0.95%) | 3.78 | 4.61E-23 | 2.63E-21 | Altered group |
| CKM | 19q13.32 | 40 (17.39%) | 137 (1.28%) | 3.77 | 1.78E-30 | 1.17E-28 | Altered group |
| ERCC1 | 19q13.32 | 39 (16.96%) | 133 (1.24%) | 3.77 | 8.88E-30 | 5.70E-28 | Altered group |
| SIX5 | 19q13.32 | 36 (15.65%) | 123 (1.15%) | 3.77 | 1.73E-27 | 1.06E-25 | Altered group |
| FAM90A27P | 19q13.42 | 22 (9.57%) | 75 (0.70%) | 3.77 | 3.80E-17 | 1.78E-15 | Altered group |
| HNRNPUL1 | 19q13.2 | 53 (23.04%) | 183 (1.71%) | 3.76 | 2.45E-40 | 2.05E-38 | Altered group |
| GPR4 | 19q13.32 | 36 (15.65%) | 124 (1.16%) | 3.76 | 2.19E-27 | 1.34E-25 | Altered group |
| CDC42EP5 | 19q13.42 | 25 (10.87%) | 86 (0.80%) | 3.76 | 2.90E-19 | 1.56E-17 | Altered group |
| TPM3P9 | 19q13.42 | 23 (10.00%) | 79 (0.74%) | 3.76 | 8.12E-18 | 4.02E-16 | Altered group |
| KDELR1 | 19q13.33 | 20 (8.70%) | 69 (0.64%) | 3.76 | 1.32E-15 | 5.36E-14 | Altered group |
| ZNF225 | 19q13.31 | 43 (18.70%) | 149 (1.39%) | 3.75 | 1.40E-32 | 9.76E-31 | Altered group |
| ZNF761 | 19q13.42 | 23 (10.00%) | 80 (0.75%) | 3.75 | 1.03E-17 | 5.04E-16 | Altered group |
| FGF21 | 19q13.33 | 21 (9.13%) | 73 (0.68%) | 3.75 | 2.84E-16 | 1.21E-14 | Altered group |
| LIN7B | 19q13.33 | 19 (8.26%) | 66 (0.62%) | 3.75 | 7.75E-15 | 2.91E-13 | Altered group |
| BSPH1 | 19q13.33 | 17 (7.39%) | 59 (0.55%) | 3.75 | 2.10E-13 | 7.16E-12 | Altered group |
| KCNN4 | 19q13.31 | 40 (17.39%) | 140 (1.30%) | 3.74 | 3.61E-30 | 2.35E-28 | Altered group |
| CBLC | 19q13.32 | 45 (19.57%) | 158 (1.47%) | 3.73 | 6.84E-34 | 5.01E-32 | Altered group |
| CEACAM8 | 19q13.2 | 38 (16.52%) | 136 (1.27%) | 3.7 | 2.26E-28 | 1.42E-26 | Altered group |
| MAMSTR | 19q13.33 | 20 (8.70%) | 72 (0.67%) | 3.7 | 2.63E-15 | 1.04E-13 | Altered group |
| NKG7 | 19q13.41 | 24 (10.43%) | 87 (0.81%) | 3.69 | 4.32E-18 | 2.17E-16 | Altered group |
| BAX | 19q13.33 | 23 (10.00%) | 83 (0.77%) | 3.69 | 2.04E-17 | 9.82E-16 | Altered group |
| XRCC1 | 19q13.31 | 42 (18.26%) | 153 (1.43%) | 3.68 | 4.47E-31 | 3.01E-29 | Altered group |
| FTL | 19q13.33 | 19 (8.26%) | 69 (0.64%) | 3.68 | 1.54E-14 | 5.63E-13 | Altered group |
| TMEM145 | 19q13.2 | 42 (18.26%) | 154 (1.44%) | 3.67 | 5.60E-31 | 3.74E-29 | Altered group |
| CGB1 | 19q13.33 | 19 (8.26%) | 70 (0.65%) | 3.66 | 1.92E-14 | 7.00E-13 | Altered group |
| EMP3 | 19q13.33 | 19 (8.26%) | 70 (0.65%) | 3.66 | 1.92E-14 | 7.00E-13 | Altered group |
| ZNF224 | 19q13.31 | 43 (18.70%) | 161 (1.50%) | 3.64 | 2.14E-31 | 1.45E-29 | Altered group |
| FOXA3 | 19q13.32 | 34 (14.78%) | 127 (1.18%) | 3.64 | 6.45E-25 | 3.75E-23 | Altered group |
| ZNF865 | 19q13.42 | 26 (11.30%) | 97 (0.90%) | 3.64 | 2.98E-19 | 1.60E-17 | Altered group |
| PSG5 | 19q13.31 | 45 (19.57%) | 169 (1.58%) | 3.63 | 8.18E-33 | 5.76E-31 | Altered group |
| GRWD1 | 19q13.33 | 22 (9.57%) | 83 (0.77%) | 3.63 | 2.32E-16 | 1.01E-14 | Altered group |
| PRRG2 | 19q13.33 | 20 (8.70%) | 76 (0.71%) | 3.62 | 6.33E-15 | 2.41E-13 | Altered group |
| SBK3 | 19q13.42 | 26 (11.30%) | 99 (0.92%) | 3.61 | 4.60E-19 | 2.45E-17 | Altered group |
| ZNF404 | 19q13.31 | 41 (17.83%) | 158 (1.47%) | 3.6 | 1.62E-29 | 1.03E-27 | Altered group |
| DMPK | 19q13.32 | 37 (16.09%) | 142 (1.32%) | 3.6 | 1.02E-26 | 6.14E-25 | Altered group |
| RFPL4AL1 | 19q13.42 | 26 (11.30%) | 100 (0.93%) | 3.6 | 5.69E-19 | 3.01E-17 | Altered group |
| TMEM238 | 19q13.42 | 26 (11.30%) | 100 (0.93%) | 3.6 | 5.69E-19 | 3.01E-17 | Altered group |
| ERVV-1 | 19q13.41 | 21 (9.13%) | 81 (0.75%) | 3.6 | 1.67E-15 | 6.70E-14 | Altered group |
| RRAS | 19q13.33 | 21 (9.13%) | 81 (0.75%) | 3.6 | 1.67E-15 | 6.70E-14 | Altered group |
| PPP5D1P | 19q13.32 | 20 (8.70%) | 77 (0.72%) | 3.6 | 7.83E-15 | 2.93E-13 | Altered group |
| TSHZ3 | 19q12 | 131 (56.96%) | 507 (4.73%) | 3.59 | 1.80E-103 | 1.95E-101 | Altered group |
| DHDH | 19q13.33 | 23 (10.00%) | 89 (0.83%) | 3.59 | 7.52E-17 | 3.47E-15 | Altered group |
| FKRP | 19q13.32 | 22 (9.57%) | 85 (0.79%) | 3.59 | 3.55E-16 | 1.50E-14 | Altered group |
| PTGIR | 19q13.32 | 21 (9.13%) | 82 (0.76%) | 3.58 | 2.06E-15 | 8.20E-14 | Altered group |
| SYNGR4 | 19q13.33 | 19 (8.26%) | 74 (0.69%) | 3.58 | 4.52E-14 | 1.61E-12 | Altered group |
| NECTIN2 | 19q13.32 | 45 (19.57%) | 178 (1.66%) | 3.56 | 5.57E-32 | 3.81E-30 | Altered group |
| ZNF284 | 19q13.31 | 43 (18.70%) | 170 (1.58%) | 3.56 | 1.46E-30 | 9.62E-29 | Altered group |
| NTF4 | 19q13.33 | 18 (7.83%) | 71 (0.66%) | 3.56 | 2.59E-13 | 8.76E-12 | Altered group |
| ZNF574 | 19q13.2 | 42 (18.26%) | 169 (1.58%) | 3.54 | 1.38E-29 | 8.78E-28 | Altered group |
| FBXO46 | 19q13.32 | 36 (15.65%) | 144 (1.34%) | 3.54 | 1.78E-25 | 1.04E-23 | Altered group |
| HSD17B14 | 19q13.33 | 21 (9.13%) | 84 (0.78%) | 3.54 | 3.11E-15 | 1.22E-13 | Altered group |
| RPS11 | 19q13.3 | 19 (8.26%) | 76 (0.71%) | 3.54 | 6.82E-14 | 2.41E-12 | Altered group |
| FAHD2CP | 2q11.2 | 9 (3.91%) | 36 (0.34%) | 3.54 | 3.10E-07 | 4.06E-06 | Altered group |
| DMWD | 19q13.32 | 36 (15.65%) | 145 (1.35%) | 3.53 | 2.18E-25 | 1.28E-23 | Altered group |
| TMEM86B | 19q13.42 | 29 (12.61%) | 117 (1.09%) | 3.53 | 1.45E-20 | 7.97E-19 | Altered group |
| BCL2L12 | 19q13.33 | 22 (9.57%) | 89 (0.83%) | 3.53 | 8.08E-16 | 3.33E-14 | Altered group |
