# Supplementary material for: Integrative Pan‐Cancer Characterization of lncRNA UPK1A‐AS1 and Its Role in Hypoxia‐Associated Sorafenib Resistance in Hepatocellular Carcinoma
Source: Anal Cell Pathol (Amst). 2026 Sep 1;2026:1554526. doi: 10.1155/ancp/1554526 (PMC13532061; doi:10.1155/ancp/1554526)
Supplement: Supplementary file 1 [file ANCP-2026-1554526-s001.zip › Table S2.docx]

**Table S2**

| **Sheet1 Differentially expressed genes associated with UPK1A-AS1 in BLCA.** | | | | | |
| --- | --- | --- | --- | --- | --- |
| gene_name | log₂FC | logCPM | F | PValue | padj |
| TTR | 4.6737028 | 4.2503885 | 101.5083 | 1.702E-21 | 1.318E-18 |
| TF | 3.8479534 | 4.6480161 | 105.89345 | 2.901E-22 | 3.074E-19 |
| PRSS2 | 3.4091652 | 5.3473781 | 55.85216 | 4.702E-13 | 1.796E-11 |
| GPC3 | 3.2694073 | 5.7549901 | 122.25317 | 4.51E-25 | 1.009E-21 |
| UGT2B15 | 3.1355835 | 2.4716014 | 71.132777 | 5.645E-16 | 6.857E-14 |
| KRT33A | 2.9948131 | 1.5496604 | 84.03366 | 2.308E-18 | 7.038E-16 |
| TAC3 | 2.9109932 | 5.9998318 | 44.028842 | 1.017E-10 | 1.762E-09 |
| FABP3 | 2.910334 | 5.0983349 | 129.16984 | 3.115E-26 | 1.568E-22 |
| GLP1R | 2.719403 | 0.3643327 | 111.06258 | 3.676E-23 | 4.934E-20 |
| TCAP | 2.689574 | 1.4816676 | 135.56082 | 2.719E-27 | 2.737E-23 |
| MYH7B | 2.4559833 | 3.3326229 | 95.807669 | 1.739E-20 | 1E-17 |
| PEG3 | 2.4431901 | 0.6384983 | 64.22733 | 1.145E-14 | 8.41E-13 |
| AP005432.2 | 2.3532675 | 3.2022829 | 59.31768 | 1.002E-13 | 5.066E-12 |
| CTNND2 | 2.3102772 | 0.9677708 | 57.583028 | 2.169E-13 | 9.532E-12 |
| PLPPR1 | 2.2549638 | 0.9441806 | 46.344655 | 3.503E-11 | 6.968E-10 |
| AL135999.3 | 2.2331472 | 1.4671289 | 60.47569 | 5.992E-14 | 3.35E-12 |
| GP6 | 2.1950502 | 0.302953 | 82.458708 | 4.479E-18 | 1.288E-15 |
| Z82217.1 | 2.1698484 | -0.786999 | 113.94168 | 1.174E-23 | 1.818E-20 |
| COL2A1 | 2.1371806 | 2.2200992 | 35.237265 | 6.186E-09 | 6.28E-08 |
| AL121895.1 | 2.1313206 | -0.941083 | 124.60252 | 1.812E-25 | 4.56E-22 |
| HSPD1P6 | 2.1200856 | 1.1259533 | 67.325881 | 2.949E-15 | 2.674E-13 |
| AC091544.2 | 2.1105676 | -0.14056 | 63.117747 | 1.865E-14 | 1.264E-12 |
| AC026369.1 | 2.1060435 | 2.8962732 | 40.991779 | 4.157E-10 | 5.902E-09 |
| CRTAC1 | 2.0964013 | 5.7725846 | 32.281203 | 2.52E-08 | 2.178E-07 |
| LEAP2 | 2.0873015 | 2.442218 | 73.892153 | 1.718E-16 | 2.572E-14 |
| RELN | 1.9985587 | 2.1353857 | 39.724717 | 7.505E-10 | 9.881E-09 |
| KSR2 | 1.9709644 | 2.354523 | 81.849848 | 5.791E-18 | 1.597E-15 |
| AC010329.2 | 1.9627833 | 0.9349161 | 31.923492 | 2.99E-08 | 2.524E-07 |
| UPK1A | 1.9531152 | 7.3256568 | 38.813373 | 1.149E-09 | 1.432E-08 |
| MSMB | 1.9503576 | 3.3890266 | 30.354158 | 6.341E-08 | 4.872E-07 |
| CTNNA3 | 1.9450346 | 0.028077 | 56.086004 | 4.235E-13 | 1.652E-11 |
| PDZD3 | 1.9441477 | 1.0372351 | 47.530961 | 2.034E-11 | 4.393E-10 |
| PCDH10 | 1.9268046 | 1.8020895 | 48.128035 | 1.548E-11 | 3.463E-10 |
| LINC02668 | 1.9227156 | 0.2808234 | 45.381344 | 5.453E-11 | 1.032E-09 |
| PM20D1 | 1.9103096 | 6.2888518 | 27.113737 | 3.031E-07 | 1.924E-06 |
| DAB1 | 1.8990531 | 3.0169178 | 39.095795 | 1.007E-09 | 1.274E-08 |
| SOX2-OT | 1.8876676 | 1.4194364 | 50.567348 | 5.098E-12 | 1.34E-10 |
| ACSM6 | 1.8852196 | 1.8410101 | 36.423949 | 3.532E-09 | 3.839E-08 |
| AL031668.2 | 1.885138 | 0.672059 | 73.77599 | 1.806E-16 | 2.654E-14 |
| CRH | 1.8850825 | 5.7011703 | 18.758931 | 1.864E-05 | 7.466E-05 |
| ERBB4 | 1.8776339 | 0.7900467 | 49.106091 | 9.91E-12 | 2.369E-10 |
| AC011503.1 | 1.8593146 | 1.0782633 | 41.58998 | 3.147E-10 | 4.624E-09 |
| AC021491.4 | 1.8567673 | 0.2288915 | 97.506129 | 8.677E-21 | 5.823E-18 |
| CLCNKB | 1.8546867 | -0.546054 | 71.114742 | 5.689E-16 | 6.858E-14 |
| SLCO4C1 | 1.8535295 | 1.9229368 | 52.831075 | 1.83E-12 | 5.598E-11 |
| UPK3A | 1.833516 | 6.3752253 | 33.428004 | 1.459E-08 | 1.346E-07 |
| AL606500.1 | 1.827454 | 2.2511692 | 25.802028 | 5.737E-07 | 3.386E-06 |
| SCNN1G | 1.8272452 | 5.6262073 | 52.794085 | 1.861E-12 | 5.666E-11 |
| B3GAT1-DT | 1.8229276 | 2.6993956 | 30.034862 | 7.392E-08 | 5.584E-07 |
| ADGRG2 | 1.8176438 | 1.4859456 | 54.433191 | 8.89E-13 | 3.086E-11 |
| COL9A1 | 1.8113348 | 0.8537113 | 41.119382 | 3.917E-10 | 5.624E-09 |
| HS3ST5 | 1.804321 | 0.9151196 | 41.008291 | 4.125E-10 | 5.868E-09 |
| FER1L5 | 1.7797458 | -0.787939 | 79.170261 | 1.802E-17 | 4.169E-15 |
| DKK4 | 1.7791454 | 2.2608217 | 30.982048 | 4.692E-08 | 3.745E-07 |
| AXDND1 | 1.7763561 | 0.5859212 | 47.462747 | 2.099E-11 | 4.513E-10 |
| EMX2 | 1.7751108 | 3.2406976 | 40.870021 | 4.399E-10 | 6.197E-09 |
| ZNF350-AS1 | 1.7740839 | 4.6605436 | 27.581704 | 2.415E-07 | 1.578E-06 |
| RDH10-AS1 | 1.7687762 | -0.08381 | 63.054787 | 1.917E-14 | 1.291E-12 |
| AGAP11 | 1.7622839 | -1.015039 | 60.588966 | 5.698E-14 | 3.222E-12 |
| AC020951.1 | 1.7613576 | -1.275816 | 74.659896 | 1.236E-16 | 1.99E-14 |
| SLC6A4 | 1.7574615 | 0.9039532 | 62.385315 | 2.575E-14 | 1.625E-12 |
| TRHDE-AS1 | 1.7560803 | 3.9714336 | 35.672492 | 5.036E-09 | 5.245E-08 |
| AL391650.2 | 1.7452926 | 1.0064856 | 29.43295 | 9.875E-08 | 7.2E-07 |
| AC092828.1 | 1.744627 | -0.509789 | 81.9571 | 5.535E-18 | 1.547E-15 |
| AADAC | 1.7443901 | 3.4865958 | 28.728901 | 1.387E-07 | 9.705E-07 |
| AC091544.4 | 1.7417166 | 0.6743084 | 45.331353 | 5.58E-11 | 1.053E-09 |
| EGFEM1P | 1.7374839 | -0.664824 | 53.371261 | 1.434E-12 | 4.582E-11 |
| BHMT | 1.7340158 | 5.5711995 | 23.419854 | 1.842E-06 | 9.562E-06 |
| AC011498.4 | 1.7265624 | -1.156765 | 67.78555 | 2.413E-15 | 2.279E-13 |
| LINC00967 | 1.726251 | 2.6050401 | 18.890678 | 1.745E-05 | 7.034E-05 |
| FAT3 | 1.7235416 | 1.9134008 | 53.40804 | 1.41E-12 | 4.514E-11 |
| GDF7 | 1.7221424 | 2.8981673 | 84.955232 | 1.567E-18 | 5.007E-16 |
| SGK2 | 1.7147637 | 3.3701823 | 46.679354 | 3.004E-11 | 6.127E-10 |
| PTCHD1 | 1.7037189 | 3.2503863 | 34.106266 | 1.057E-08 | 1.013E-07 |
| AC016717.2 | 1.6975924 | 0.263993 | 34.562946 | 8.514E-09 | 8.364E-08 |
| AC026803.3 | 1.6947664 | -1.387841 | 95.140797 | 2.287E-20 | 1.18E-17 |
| AC005856.1 | 1.6858977 | -0.901167 | 80.67663 | 9.511E-18 | 2.335E-15 |
| CEACAM7 | 1.6810239 | 4.5690351 | 18.38686 | 2.246E-05 | 8.843E-05 |
| AL117329.1 | 1.6683834 | 1.5547851 | 37.396977 | 2.234E-09 | 2.589E-08 |
| FRRS1L | 1.6577663 | -0.136 | 45.305255 | 5.647E-11 | 1.063E-09 |
| AC008679.1 | 1.652039 | -0.745483 | 40.44473 | 5.363E-10 | 7.385E-09 |
| CA4 | 1.6486395 | 2.1839147 | 26.307342 | 4.485E-07 | 2.714E-06 |
| AC015849.5 | 1.647467 | 1.0357691 | 59.347102 | 9.887E-14 | 5.013E-12 |
| AC019117.1 | 1.6425696 | 3.375018 | 43.910092 | 1.074E-10 | 1.847E-09 |
| LINC02302 | 1.6423877 | 0.1226112 | 35.045924 | 6.773E-09 | 6.803E-08 |
| TFF2 | 1.6412068 | 2.5446612 | 22.192308 | 3.374E-06 | 1.649E-05 |
| B3GAT1 | 1.633325 | 2.6877052 | 37.973639 | 1.704E-09 | 2.033E-08 |
| AL772337.3 | 1.6311285 | -0.415404 | 33.91715 | 1.157E-08 | 1.094E-07 |
| FAM189A1 | 1.6285131 | 2.2396856 | 35.030666 | 6.822E-09 | 6.845E-08 |
| HMGCS2 | 1.627997 | 8.0330688 | 21.392581 | 5.013E-06 | 2.348E-05 |
| NR1H4 | 1.6234364 | 1.5658952 | 22.918863 | 2.357E-06 | 1.192E-05 |
| KRT20 | 1.6211862 | 7.5603228 | 22.782704 | 2.521E-06 | 1.268E-05 |
| AC023421.2 | 1.6139986 | -0.203716 | 39.290113 | 9.195E-10 | 1.179E-08 |
| SNX31 | 1.6096967 | 6.2689665 | 31.073747 | 4.49E-08 | 3.595E-07 |
| LINC02038 | 1.6064647 | -0.612681 | 40.642087 | 4.892E-10 | 6.806E-09 |
| FMO9P | 1.6060927 | 4.8316856 | 25.208727 | 7.664E-07 | 4.376E-06 |
| SCUBE2 | 1.6054343 | 6.2649587 | 43.628313 | 1.224E-10 | 2.06E-09 |
| EVX1-AS | 1.6031349 | 0.3326049 | 29.796483 | 8.29E-08 | 6.178E-07 |
| PTPRT | 1.6023959 | 0.1949339 | 30.56318 | 5.735E-08 | 4.465E-07 |
| CHAD | 1.5906755 | 0.0666169 | 100.32712 | 2.749E-21 | 2.05E-18 |
| LINC01622 | 1.5898582 | -0.232979 | 36.433978 | 3.515E-09 | 3.825E-08 |
| PVALB | 1.5833731 | 3.8387056 | 18.202487 | 2.465E-05 | 9.602E-05 |
| KCNH2 | 1.5820765 | 2.9007633 | 40.661467 | 4.848E-10 | 6.76E-09 |
| AC007878.1 | 1.5731375 | -0.929639 | 71.746221 | 4.331E-16 | 5.448E-14 |
| AC010300.1 | 1.5730586 | 0.9187141 | 68.922729 | 1.471E-15 | 1.481E-13 |
| TSPEAR-AS1 | 1.572206 | 0.1960691 | 29.308611 | 1.048E-07 | 7.589E-07 |
| GABBR2 | 1.5716169 | 4.5994959 | 25.141317 | 7.92E-07 | 4.496E-06 |
| CACNA1I | 1.5700671 | 1.9850654 | 39.613253 | 7.906E-10 | 1.033E-08 |
| CTXND1 | 1.5680326 | 2.3520804 | 25.158306 | 7.855E-07 | 4.465E-06 |
| AC011487.2 | 1.5651997 | 4.7394758 | 18.200767 | 2.467E-05 | 9.608E-05 |
| SLC30A3 | 1.5584962 | 0.4733671 | 37.204041 | 2.446E-09 | 2.804E-08 |
| FREM2 | 1.5580112 | 4.0901816 | 23.093022 | 2.164E-06 | 1.104E-05 |
| BPIFB1 | 1.5569107 | 4.5995 | 16.505511 | 5.806E-05 | 0.0002055 |
| LINC02814 | 1.5558502 | 0.8607933 | 40.955118 | 4.228E-10 | 5.986E-09 |
| AC108519.1 | 1.5535821 | 0.5454664 | 45.951623 | 4.196E-11 | 8.152E-10 |
| FAM78B | 1.5534827 | 3.0696758 | 44.762401 | 7.251E-11 | 1.316E-09 |
| UPK2 | 1.5533347 | 8.562102 | 29.01299 | 1.209E-07 | 8.594E-07 |
| CTSE | 1.5485792 | 6.241542 | 19.715717 | 1.155E-05 | 4.893E-05 |
| AC000093.1 | 1.5428633 | -1.282403 | 55.509246 | 5.484E-13 | 2.018E-11 |
| MTUS2 | 1.5402443 | 2.8517035 | 28.30681 | 1.7E-07 | 1.16E-06 |
| LINC01668 | 1.5381839 | 0.2110477 | 28.896399 | 1.279E-07 | 9.015E-07 |
| KIF28P | 1.5294671 | 0.1693018 | 60.308609 | 6.452E-14 | 3.568E-12 |
| AC092279.2 | 1.5278774 | 0.2154116 | 97.154185 | 1.002E-20 | 6.303E-18 |
| AL023755.1 | 1.5251918 | -0.592911 | 39.528585 | 8.225E-10 | 1.07E-08 |
| ACOXL-AS1 | 1.5222102 | 0.9931845 | 75.366504 | 9.127E-17 | 1.584E-14 |
| CELF5 | 1.5201942 | -0.293887 | 43.262385 | 1.449E-10 | 2.376E-09 |
| TSPEAR-AS2 | 1.5152704 | 1.0059283 | 26.561111 | 3.964E-07 | 2.436E-06 |
| ADAMTS19 | 1.5126991 | -0.063534 | 28.975223 | 1.231E-07 | 8.731E-07 |
| AL442636.1 | 1.5046539 | -1.534096 | 40.223813 | 5.945E-10 | 8.075E-09 |
| SCNN1B | 1.5045909 | 6.6351082 | 40.796981 | 4.551E-10 | 6.385E-09 |
| MAB21L1 | 1.5042998 | 0.350639 | 31.475616 | 3.704E-08 | 3.038E-07 |
| EHD4-AS1 | 1.5038178 | -0.906946 | 60.524529 | 5.863E-14 | 3.297E-12 |
| AC110774.1 | 1.494717 | -0.642164 | 45.244333 | 5.808E-11 | 1.087E-09 |
| LINC00930 | 1.4928629 | 3.15496 | 23.384891 | 1.874E-06 | 9.708E-06 |
| AL355987.4 | 1.4922167 | 1.5155503 | 54.226013 | 9.759E-13 | 3.341E-11 |
| MAL2-AS1 | 1.4902252 | -1.252261 | 58.088553 | 1.731E-13 | 7.848E-12 |
| GCKR | 1.4808025 | 0.4350977 | 35.532453 | 5.38E-09 | 5.571E-08 |
| RPL37P1 | 1.4798138 | -1.284974 | 73.88334 | 1.725E-16 | 2.572E-14 |
| TRPM5 | 1.4788557 | -0.255554 | 32.744627 | 2.021E-08 | 1.783E-07 |
| AC092329.4 | 1.4787154 | 1.8358783 | 115.58701 | 6.133E-24 | 1.029E-20 |
| CD177 | 1.466229 | 2.7791342 | 21.79953 | 4.098E-06 | 1.965E-05 |
| SLC9A4 | 1.4608837 | 3.275984 | 25.978605 | 5.264E-07 | 3.136E-06 |
| TTC9B | 1.460717 | -0.547489 | 64.01091 | 1.259E-14 | 9.083E-13 |
| EML5 | 1.4564142 | -0.252091 | 37.628225 | 2.004E-09 | 2.352E-08 |
| MAL | 1.4545347 | 5.6262386 | 24.31591 | 1.186E-06 | 6.426E-06 |
| FXYD4 | 1.4539866 | 4.0196143 | 17.731992 | 3.123E-05 | 0.0001183 |
| DLX6-AS1 | 1.4517315 | 2.1962224 | 43.700922 | 1.183E-10 | 2.002E-09 |
| TMPRSS2 | 1.450924 | 6.6894778 | 37.122764 | 2.541E-09 | 2.894E-08 |
| SLC30A2 | 1.4492372 | 4.3073577 | 25.501337 | 6.643E-07 | 3.863E-06 |
| ABCG2 | 1.4427815 | 3.3972158 | 39.994888 | 6.615E-10 | 8.878E-09 |
| ERVE-1 | 1.4425733 | 2.6018486 | 37.981266 | 1.698E-09 | 2.027E-08 |
| AC011447.6 | 1.4407887 | -0.978352 | 59.119794 | 1.094E-13 | 5.436E-12 |
| LINC01671 | 1.4394283 | 2.2832765 | 33.357375 | 1.509E-08 | 1.384E-07 |
| MTND1P23 | 1.4388888 | 4.2978379 | 15.414184 | 0.0001011 | 0.0003345 |
| SYT3 | 1.4388176 | -0.826598 | 43.109364 | 1.556E-10 | 2.517E-09 |
| AL157786.1 | 1.4301752 | 0.126286 | 90.995638 | 1.264E-19 | 5.191E-17 |
| EMX2OS | 1.4279259 | 2.8312217 | 30.636506 | 5.537E-08 | 4.325E-07 |
| SGSM1 | 1.4246416 | 2.2238135 | 50.589874 | 5.047E-12 | 1.33E-10 |
| PRR26 | 1.4208539 | 0.1337634 | 46.416025 | 3.39E-11 | 6.77E-10 |
| IGDCC3 | 1.4158363 | 1.9387182 | 20.450709 | 8.004E-06 | 3.543E-05 |
| CRISP3 | 1.4155723 | 3.5821303 | 16.59082 | 5.56E-05 | 0.0001974 |
| AOC2 | 1.4132099 | 2.2602944 | 81.478082 | 6.776E-18 | 1.816E-15 |
| ZNF66 | 1.4121686 | 2.7430722 | 61.169997 | 4.406E-14 | 2.616E-12 |
| LINC02593 | 1.4107724 | 1.6337436 | 42.390291 | 2.171E-10 | 3.343E-09 |
| FUT9 | 1.4058562 | 2.966599 | 30.937798 | 4.792E-08 | 3.81E-07 |
| CD244 | 1.4042459 | 1.825099 | 33.998374 | 1.113E-08 | 1.058E-07 |
| TMEM97 | 1.404012 | 7.394264 | 77.875358 | 3.125E-17 | 6.486E-15 |
| SPINK1 | 1.402092 | 9.0838853 | 16.157849 | 6.926E-05 | 0.0002399 |
| PACSIN1 | 1.4017961 | 2.1073115 | 63.219226 | 1.783E-14 | 1.221E-12 |
| JPH4 | 1.4013871 | 0.8606504 | 48.325038 | 1.415E-11 | 3.193E-10 |
| SLC5A7 | 1.4011558 | 1.1873495 | 22.9626 | 2.307E-06 | 1.171E-05 |
| AL591848.3 | 1.3982938 | 0.9960691 | 73.091116 | 2.425E-16 | 3.367E-14 |
| ST3GAL5-AS1 | 1.3955769 | -1.076702 | 49.568343 | 8.029E-12 | 1.983E-10 |
| FGF12 | 1.3924781 | 2.6565839 | 49.065822 | 1.009E-11 | 2.403E-10 |
| PKHD1 | 1.3908495 | 2.038672 | 30.404738 | 6.189E-08 | 4.77E-07 |
| VIPR1-AS1 | 1.3905736 | -0.120399 | 56.822243 | 3.046E-13 | 1.262E-11 |
| KIF5C | 1.3905545 | 3.9578325 | 58.753693 | 1.287E-13 | 6.186E-12 |
| ANKRD20A11P | 1.3886815 | 0.1360553 | 30.590857 | 5.66E-08 | 4.409E-07 |
| TTC23L | 1.388044 | 0.5548732 | 68.61501 | 1.682E-15 | 1.652E-13 |
| SLC16A12 | 1.3792942 | -0.064812 | 27.595821 | 2.399E-07 | 1.568E-06 |
| OVGP1 | 1.3789334 | 2.7657491 | 65.964812 | 5.344E-15 | 4.513E-13 |
| PTH2R | 1.3768062 | 2.0495447 | 19.424938 | 1.335E-05 | 5.559E-05 |
| MYCL-AS1 | 1.3766828 | -1.03611 | 43.15659 | 1.522E-10 | 2.469E-09 |
| LINC02701 | 1.3751367 | 0.5833183 | 52.599539 | 2.031E-12 | 6.103E-11 |
| SLC9A2 | 1.3746246 | 4.2204775 | 38.185928 | 1.542E-09 | 1.856E-08 |
| TOX3 | 1.3739627 | 4.8403914 | 19.240379 | 1.464E-05 | 6.03E-05 |
| ITLN1 | 1.3733048 | 5.3181753 | 9.7683717 | 0.0019006 | 0.0045722 |
| G6PC | 1.3711483 | -0.894366 | 30.93358 | 4.802E-08 | 3.815E-07 |
| SCN4A | 1.3704175 | -0.676271 | 39.291385 | 9.19E-10 | 1.179E-08 |
| KIF1A | 1.369857 | 3.0080111 | 15.275834 | 0.0001085 | 0.0003557 |
| CYP1A1 | 1.3644321 | 7.5986639 | 8.3861373 | 0.0039816 | 0.0088173 |
| NLRP7 | 1.3620866 | 4.2810373 | 13.940995 | 0.0002151 | 0.0006549 |
| PKD1L1 | 1.3597781 | 2.3247579 | 43.319659 | 1.411E-10 | 2.321E-09 |
| SCN3A | 1.359354 | 0.1191233 | 28.97012 | 1.234E-07 | 8.749E-07 |
| MAGI1-IT1 | 1.3570046 | -1.553955 | 58.079395 | 1.738E-13 | 7.862E-12 |
| AC007998.4 | 1.3539756 | 2.1800825 | 26.855216 | 3.436E-07 | 2.153E-06 |
| AC107419.1 | 1.3518719 | -0.621532 | 26.769196 | 3.583E-07 | 2.229E-06 |
| AC127024.8 | 1.3514563 | -1.41309 | 64.236552 | 1.14E-14 | 8.41E-13 |
| AC018865.2 | 1.350866 | 0.4215125 | 20.691226 | 7.101E-06 | 3.189E-05 |
| HELLPAR | 1.3502339 | 1.2642286 | 62.799492 | 2.146E-14 | 1.415E-12 |
| KCNH8 | 1.3491799 | 0.6470075 | 28.480236 | 1.564E-07 | 1.077E-06 |
| TEPP | 1.347159 | 2.3069677 | 20.78501 | 6.778E-06 | 3.059E-05 |
| DMRT2 | 1.3454262 | 0.8947482 | 21.856774 | 3.983E-06 | 1.915E-05 |
| AC093690.1 | 1.3449138 | -1.413929 | 84.120429 | 2.225E-18 | 6.891E-16 |
| SLC26A7 | 1.3444997 | -0.507148 | 39.910197 | 6.882E-10 | 9.169E-09 |
| DPY19L2 | 1.3428275 | 1.6675703 | 46.811404 | 2.828E-11 | 5.833E-10 |
| CCSER1 | 1.3418785 | 3.1342751 | 54.128674 | 1.02E-12 | 3.461E-11 |
| POU4F1 | 1.3366295 | 0.0770106 | 22.935852 | 2.338E-06 | 1.184E-05 |
| SHISA2 | 1.3337755 | 4.1854803 | 22.209634 | 3.345E-06 | 1.636E-05 |
| AC010329.1 | 1.3311237 | 3.8170734 | 23.093018 | 2.164E-06 | 1.104E-05 |
| SELL | 1.3304246 | 5.8521204 | 25.535366 | 6.534E-07 | 3.806E-06 |
| ERICH5 | 1.3304244 | 3.0698545 | 45.24272 | 5.812E-11 | 1.087E-09 |
| LINC01933 | 1.3301706 | -1.00703 | 31.746087 | 3.255E-08 | 2.708E-07 |
| MALAT1 | 1.3294313 | 8.4077846 | 68.136957 | 2.071E-15 | 2.007E-13 |
| ONECUT2 | 1.3286654 | 3.282692 | 25.693428 | 6.049E-07 | 3.551E-06 |
| SCN7A | 1.3281701 | 0.4314386 | 20.731591 | 6.96E-06 | 3.134E-05 |
| AC022137.3 | 1.3257443 | -0.891898 | 56.855384 | 3.001E-13 | 1.248E-11 |
| DHRS2 | 1.3238882 | 10.110038 | 21.408973 | 4.972E-06 | 2.331E-05 |
| LRRC4B | 1.3223454 | 1.1827716 | 57.306317 | 2.454E-13 | 1.046E-11 |
| OTOGL | 1.3202845 | -0.252648 | 44.205115 | 9.376E-11 | 1.65E-09 |
| MAGEL2 | 1.3180789 | 0.2045345 | 30.743521 | 5.26E-08 | 4.133E-07 |
| AC129926.1 | 1.3171767 | 0.4722384 | 26.734448 | 3.644E-07 | 2.26E-06 |
| AP001625.2 | 1.3146946 | -1.274401 | 69.470412 | 1.16E-15 | 1.202E-13 |
| AL450263.1 | 1.3139528 | -1.887588 | 72.706353 | 2.862E-16 | 3.946E-14 |
| MIR3189 | 1.3099177 | -0.519654 | 32.70538 | 2.059E-08 | 1.814E-07 |
| AC131649.2 | 1.3087417 | 0.8163325 | 55.157168 | 6.422E-13 | 2.317E-11 |
| PTGDR2 | 1.3081542 | 0.4950671 | 24.071039 | 1.338E-06 | 7.171E-06 |
| ZNF737 | 1.3061372 | 5.3114116 | 44.867465 | 6.909E-11 | 1.264E-09 |
| MLC1 | 1.3046744 | -0.23665 | 36.343453 | 3.669E-09 | 3.951E-08 |
| IPO5P1 | 1.3046637 | 3.64633 | 93.187393 | 5.108E-20 | 2.337E-17 |
| TMEM191C | 1.3038986 | 0.1291183 | 62.521436 | 2.425E-14 | 1.54E-12 |
| AP001094.2 | 1.3018176 | -0.358888 | 47.333627 | 2.226E-11 | 4.747E-10 |
| LINC01977 | 1.3012452 | 0.2914579 | 69.672567 | 1.063E-15 | 1.114E-13 |
| IGLON5 | 1.2997514 | 0.4273101 | 28.655734 | 1.437E-07 | 1.001E-06 |
| WFDC13 | 1.2936067 | -0.997298 | 27.931086 | 2.039E-07 | 1.364E-06 |
| AC090061.1 | 1.2931788 | -0.482794 | 57.537732 | 2.213E-13 | 9.642E-12 |
| PI4KAP1 | 1.2877627 | 3.7454124 | 59.493844 | 9.263E-14 | 4.769E-12 |
| MYCL | 1.2872083 | 7.0965972 | 56.310449 | 3.83E-13 | 1.521E-11 |
| PROM1 | 1.2862596 | 3.2965347 | 17.242897 | 3.997E-05 | 0.0001471 |
| CECR2 | 1.2860438 | 3.1295979 | 28.229552 | 1.765E-07 | 1.2E-06 |
| ZNF730 | 1.2849089 | -0.220847 | 34.561851 | 8.518E-09 | 8.364E-08 |
| AC135178.4 | 1.2848894 | -0.27437 | 50.014567 | 6.554E-12 | 1.674E-10 |
| PPP1R3C | 1.2844916 | 4.9788463 | 41.039686 | 4.065E-10 | 5.791E-09 |
| MYRFL | 1.2839652 | -0.209627 | 34.236789 | 9.938E-09 | 9.595E-08 |
| AC133041.1 | 1.2837844 | -0.004217 | 20.12039 | 9.435E-06 | 4.097E-05 |
| SYN2 | 1.2835876 | 2.1125258 | 28.53917 | 1.52E-07 | 1.052E-06 |
| VGF | 1.2832982 | 1.5259108 | 31.964309 | 2.932E-08 | 2.481E-07 |
| SH3GL2 | 1.2829828 | 2.0796272 | 15.399541 | 0.0001019 | 0.0003366 |
| AC004593.1 | 1.2794536 | -1.304021 | 33.043467 | 1.752E-08 | 1.576E-07 |
| FTCD | 1.277964 | -0.146313 | 27.409132 | 2.626E-07 | 1.698E-06 |
| HIC2 | 1.2778518 | 4.6902806 | 68.993266 | 1.427E-15 | 1.443E-13 |
| SLC17A7 | 1.2775798 | 0.4428443 | 42.421127 | 2.14E-10 | 3.303E-09 |
| ADAM11 | 1.2766622 | 1.3245166 | 71.491149 | 4.835E-16 | 6.008E-14 |
| LINC01088 | 1.2757669 | 1.1608263 | 26.822689 | 3.491E-07 | 2.183E-06 |
| LINC01833 | 1.2678998 | 2.414589 | 22.556467 | 2.819E-06 | 1.404E-05 |
| GPC2 | 1.2657577 | 2.7726178 | 51.13498 | 3.941E-12 | 1.076E-10 |
| PWRN1 | 1.2642993 | 0.0027557 | 19.017753 | 1.637E-05 | 6.654E-05 |
| AC108134.1 | 1.2642542 | 1.7175763 | 56.155383 | 4.105E-13 | 1.611E-11 |
| DAAM2-AS1 | 1.2638291 | -0.76129 | 51.020595 | 4.151E-12 | 1.126E-10 |
| PALM3 | 1.2630934 | 4.9574459 | 29.60437 | 9.093E-08 | 6.704E-07 |
| PRSS43P | 1.261069 | -0.52855 | 25.447669 | 6.819E-07 | 3.955E-06 |
| PI4KAP2 | 1.2601624 | 3.501372 | 81.402649 | 6.996E-18 | 1.829E-15 |
| PACRG | 1.2584953 | 0.9650928 | 26.550756 | 3.984E-07 | 2.446E-06 |
| ZBTB8B | 1.2584743 | 1.4361284 | 38.686806 | 1.22E-09 | 1.509E-08 |
| ST8SIA2 | 1.2561842 | -0.087407 | 20.887649 | 6.441E-06 | 2.924E-05 |
| AC093535.1 | 1.2555424 | -1.03453 | 63.839291 | 1.358E-14 | 9.622E-13 |
| LINC00456 | 1.2542915 | 0.3369767 | 26.868194 | 3.414E-07 | 2.141E-06 |
| SNORD123 | 1.2536728 | -1.527407 | 49.737447 | 7.434E-12 | 1.855E-10 |
| AC008759.2 | 1.2530089 | -0.876053 | 38.451074 | 1.362E-09 | 1.666E-08 |
| TCEAL2 | 1.2517148 | 0.5381679 | 18.598315 | 2.02E-05 | 8.033E-05 |
| AC096751.2 | 1.2509831 | -0.679008 | 29.490073 | 9.607E-08 | 7.033E-07 |
| KIF5A | 1.2496562 | 1.1196589 | 36.927709 | 2.786E-09 | 3.128E-08 |
| THRB-AS1 | 1.2493374 | -0.92242 | 65.083466 | 7.862E-15 | 6.162E-13 |
| PPP1R14D | 1.2480643 | 2.1968757 | 17.943316 | 2.808E-05 | 0.0001079 |
| ZNF727 | 1.246361 | 1.6301156 | 27.23518 | 2.857E-07 | 1.827E-06 |
| LINC01612 | 1.245955 | 1.085822 | 15.831682 | 8.175E-05 | 0.0002773 |
| FSIP2-AS1 | 1.2443713 | -1.407735 | 53.335655 | 1.457E-12 | 4.642E-11 |
| AC025580.1 | 1.2442335 | 0.5581443 | 17.802364 | 3.014E-05 | 0.0001149 |
| AC006042.1 | 1.2441316 | 0.5820838 | 74.936285 | 1.098E-16 | 1.826E-14 |
| AC015799.1 | 1.2425907 | -1.182319 | 55.885806 | 4.632E-13 | 1.776E-11 |
| HSD17B1 | 1.2413787 | 2.7239111 | 36.090312 | 4.134E-09 | 4.405E-08 |
| AL591848.1 | 1.2412925 | -1.073347 | 32.154207 | 2.678E-08 | 2.294E-07 |
| ACER2 | 1.2411406 | 5.4923335 | 36.886051 | 2.841E-09 | 3.177E-08 |
| GRIK4 | 1.2393656 | 0.0572864 | 40.474379 | 5.29E-10 | 7.303E-09 |
| DLGAP1 | 1.2385976 | 1.2936116 | 21.417127 | 4.952E-06 | 2.324E-05 |
| SYTL5 | 1.2384889 | 3.6907277 | 27.84214 | 2.129E-07 | 1.415E-06 |
| AC022382.2 | 1.238358 | -1.30356 | 75.925743 | 7.184E-17 | 1.32E-14 |
| FBXW12 | 1.2382651 | -0.950441 | 47.93784 | 1.689E-11 | 3.728E-10 |
| AL158206.1 | 1.2373451 | 6.9073775 | 39.01304 | 1.047E-09 | 1.319E-08 |
| AC012511.1 | 1.2371248 | -1.20899 | 35.35498 | 5.851E-09 | 5.997E-08 |
| GPLD1 | 1.2366123 | 2.4739207 | 52.900787 | 1.773E-12 | 5.442E-11 |
| FSIP2-AS2 | 1.2338208 | -1.414819 | 38.904909 | 1.101E-09 | 1.378E-08 |
| SCHLAP1 | 1.2329714 | -0.215261 | 28.868603 | 1.296E-07 | 9.127E-07 |
| AC011472.4 | 1.2292381 | -1.428726 | 64.297843 | 1.11E-14 | 8.29E-13 |
| RGS9BP | 1.2268092 | -0.545242 | 43.828926 | 1.115E-10 | 1.906E-09 |
| TESC | 1.2263289 | 4.8865291 | 23.533626 | 1.742E-06 | 9.095E-06 |
| MYO1H | 1.2262815 | -1.129223 | 47.335551 | 2.224E-11 | 4.747E-10 |
| ADCY10 | 1.226071 | -0.540332 | 33.532413 | 1.389E-08 | 1.289E-07 |
| HSP90AB4P | 1.2222626 | -1.087899 | 43.062876 | 1.59E-10 | 2.566E-09 |
| AC005041.1 | 1.2214225 | -0.831613 | 27.393765 | 2.646E-07 | 1.709E-06 |
| LINC00482 | 1.2200793 | 1.7024069 | 26.096261 | 4.97E-07 | 2.978E-06 |
| BMP3 | 1.2198273 | 5.4970434 | 20.340619 | 8.455E-06 | 3.714E-05 |
| MEGF11 | 1.2148916 | -0.854161 | 52.055613 | 2.598E-12 | 7.604E-11 |
| KLHDC7A | 1.2103015 | 5.1847976 | 23.123424 | 2.131E-06 | 1.089E-05 |
| Z99572.1 | 1.2094366 | 2.7038989 | 102.45201 | 1.162E-21 | 9.744E-19 |
| USP3-AS1 | 1.2092094 | -0.120345 | 69.460684 | 1.165E-15 | 1.202E-13 |
| FAR2P1 | 1.2086689 | -0.141568 | 17.110619 | 4.274E-05 | 0.0001561 |
| CDH23 | 1.2051721 | 4.4983785 | 42.213151 | 2.357E-10 | 3.591E-09 |
| RN7SKP23 | 1.2042793 | -1.097055 | 63.662697 | 1.467E-14 | 1.025E-12 |
| AC003681.1 | 1.2022645 | 1.9667307 | 86.651326 | 7.703E-19 | 2.542E-16 |
| RPL7AP10 | 1.2010552 | -1.485837 | 58.575659 | 1.393E-13 | 6.569E-12 |
| LY6H | 1.1997764 | 0.2266836 | 22.113132 | 3.509E-06 | 1.709E-05 |
| PGLYRP2 | 1.1951637 | -0.663695 | 24.846216 | 9.15E-07 | 5.115E-06 |
| LONRF2 | 1.1949637 | 2.0290856 | 23.552613 | 1.726E-06 | 9.027E-06 |
| RAPSN | 1.1948924 | 0.2442349 | 34.759713 | 7.756E-09 | 7.657E-08 |
| AL031280.1 | 1.1944025 | -0.078457 | 20.457462 | 7.977E-06 | 3.535E-05 |
| TBX2-AS1 | 1.1943046 | 3.5349251 | 30.939063 | 4.789E-08 | 3.809E-07 |
| AC025917.1 | 1.1924818 | -1.088105 | 44.502968 | 8.172E-11 | 1.465E-09 |
| PRR36 | 1.1921395 | 3.7979924 | 32.93042 | 1.849E-08 | 1.654E-07 |
| BCAS1 | 1.1905977 | 6.4241176 | 24.591868 | 1.036E-06 | 5.726E-06 |
| AL356740.1 | 1.1900925 | -0.274175 | 28.487305 | 1.558E-07 | 1.074E-06 |
| AL161772.1 | 1.1889788 | 1.4616232 | 46.472446 | 3.303E-11 | 6.612E-10 |
| ACP3 | 1.1869772 | 3.2826885 | 33.198284 | 1.628E-08 | 1.483E-07 |
| AZGP1 | 1.186881 | 3.3940259 | 17.716705 | 3.147E-05 | 0.0001191 |
| AC007881.4 | 1.184115 | -1.202741 | 67.373299 | 2.889E-15 | 2.631E-13 |
| CYP4F12 | 1.1834595 | 5.2620309 | 24.464002 | 1.103E-06 | 6.042E-06 |
| KRT12 | 1.1807538 | -0.453175 | 24.704414 | 9.807E-07 | 5.449E-06 |
| WFDC21P | 1.1792545 | 4.4947496 | 19.47571 | 1.302E-05 | 5.439E-05 |
| B3GALT5 | 1.1791943 | 3.6365011 | 20.4458 | 8.023E-06 | 3.548E-05 |
| TEX45 | 1.1771453 | 1.0410489 | 29.639265 | 8.941E-08 | 6.613E-07 |
| AC074135.1 | 1.1767657 | -1.275945 | 25.370925 | 7.08E-07 | 4.081E-06 |
| AP001207.3 | 1.1767361 | 1.7921042 | 28.569145 | 1.498E-07 | 1.039E-06 |
| AC116317.1 | 1.1764829 | -1.677487 | 53.614755 | 1.285E-12 | 4.199E-11 |
| AC110619.1 | 1.1725458 | 3.3461464 | 24.652489 | 1.006E-06 | 5.574E-06 |
| ACADL | 1.171815 | 0.6855201 | 25.166283 | 7.824E-07 | 4.453E-06 |
| EVX1 | 1.1710179 | 2.7416581 | 17.954198 | 2.792E-05 | 0.0001074 |
| AL161668.3 | 1.1699535 | -1.152904 | 41.408556 | 3.424E-10 | 4.999E-09 |
| CYP2C18 | 1.1683634 | 1.1239798 | 16.152309 | 6.945E-05 | 0.0002406 |
| AC006330.1 | 1.1667144 | -1.628489 | 70.561911 | 7.227E-16 | 8.189E-14 |
| FADS6 | 1.1654528 | 1.5849568 | 15.006357 | 0.0001245 | 0.0004018 |
| RHOQ-AS1 | 1.1637671 | -1.143896 | 71.02905 | 5.904E-16 | 7.045E-14 |
| ZNF91 | 1.1636051 | 4.8409138 | 97.154803 | 1.002E-20 | 6.303E-18 |
| ERBB2 | 1.1632418 | 9.2711605 | 59.212591 | 1.05E-13 | 5.269E-12 |
| SYT16 | 1.162817 | 1.9145441 | 27.931654 | 2.039E-07 | 1.364E-06 |
| AC090398.2 | 1.1612957 | -0.720643 | 51.945247 | 2.731E-12 | 7.875E-11 |
| TNK2-AS1 | 1.1608548 | -0.774085 | 65.958985 | 5.358E-15 | 4.513E-13 |
| GAS2 | 1.1606137 | 1.1565675 | 36.123811 | 4.069E-09 | 4.349E-08 |
| SLC38A4 | 1.1585486 | 4.2922052 | 14.487299 | 0.0001625 | 0.0005093 |
| AC025031.4 | 1.1575619 | -0.855035 | 69.758899 | 1.023E-15 | 1.088E-13 |
| AP001628.1 | 1.1566685 | -1.237404 | 63.173261 | 1.82E-14 | 1.242E-12 |
| SULT1A2 | 1.1559012 | -0.590012 | 24.874486 | 9.024E-07 | 5.059E-06 |
| AL356740.2 | 1.1558979 | -1.53191 | 29.670927 | 8.806E-08 | 6.52E-07 |
| ERP27 | 1.1554208 | 5.2301221 | 32.832944 | 1.937E-08 | 1.723E-07 |
| HNF1B | 1.1533281 | 4.1660174 | 20.492517 | 7.839E-06 | 3.479E-05 |
| LRRIQ4 | 1.1519736 | -0.40027 | 34.285461 | 9.711E-09 | 9.407E-08 |
| LINC01341 | 1.1451958 | 1.9030651 | 36.763874 | 3.009E-09 | 3.338E-08 |
| ACSM3 | 1.1448712 | 3.0199869 | 42.960939 | 1.666E-10 | 2.67E-09 |
| F5 | 1.1445546 | 4.3949475 | 20.919214 | 6.34E-06 | 2.881E-05 |
| COLGALT2 | 1.1439957 | 1.1632427 | 26.548678 | 3.988E-07 | 2.446E-06 |
| MFSD13B | 1.1429579 | -1.66436 | 62.734742 | 2.208E-14 | 1.438E-12 |
| AC021146.12 | 1.1422979 | -0.961503 | 20.423973 | 8.111E-06 | 3.578E-05 |
| CCNI2 | 1.1416599 | 1.7067498 | 24.105685 | 1.315E-06 | 7.06E-06 |
| TPH1 | 1.141655 | -0.762465 | 49.920936 | 6.839E-12 | 1.727E-10 |
| GDF9 | 1.139312 | -0.102174 | 75.555236 | 8.419E-17 | 1.487E-14 |
| BAMBI | 1.1363656 | 6.2888498 | 30.118152 | 7.102E-08 | 5.387E-07 |
| AC016738.1 | 1.1344278 | -0.346773 | 41.469661 | 3.328E-10 | 4.88E-09 |
| TSBP1-AS1 | 1.1338368 | -1.068634 | 33.37728 | 1.495E-08 | 1.375E-07 |
| FSIP2 | 1.1334873 | 2.4834198 | 29.000695 | 1.216E-07 | 8.636E-07 |
| AC073534.1 | 1.133439 | -0.798269 | 52.255657 | 2.373E-12 | 7.049E-11 |
| RHOU | 1.1319796 | 6.3853318 | 56.309155 | 3.832E-13 | 1.521E-11 |
| AC092279.1 | 1.1310981 | 2.8496172 | 70.344652 | 7.94E-16 | 8.795E-14 |
| LINC01224 | 1.1306696 | 1.7025005 | 21.857938 | 3.981E-06 | 1.915E-05 |
| ZNF492 | 1.1305679 | -0.135524 | 19.794817 | 1.11E-05 | 4.723E-05 |
| AC090954.1 | 1.129233 | -0.899792 | 23.032721 | 2.229E-06 | 1.135E-05 |
| SAMD11 | 1.1284378 | 1.9611293 | 34.249792 | 9.877E-09 | 9.554E-08 |
| MTDHP1 | 1.1283319 | -1.389309 | 55.360957 | 5.861E-13 | 2.133E-11 |
| RNF183 | 1.1281827 | 0.7097666 | 20.811057 | 6.69E-06 | 3.022E-05 |
| AC124944.3 | 1.1272788 | -0.725349 | 48.107096 | 1.563E-11 | 3.488E-10 |
| PLAC4 | 1.126733 | 0.6423707 | 17.454851 | 3.592E-05 | 0.0001337 |
| HIF3A | 1.1251303 | 2.0089235 | 19.535574 | 1.263E-05 | 5.296E-05 |
| RALGAPA2 | 1.1249537 | 6.5805746 | 87.446231 | 5.527E-19 | 1.918E-16 |
| PWWP3B | 1.1246208 | 2.1270329 | 24.398618 | 1.139E-06 | 6.213E-06 |
| DOC2A | 1.124474 | 0.8823898 | 22.367536 | 3.094E-06 | 1.525E-05 |
| RNASEH2B-AS1 | 1.1228339 | -0.592915 | 61.662106 | 3.544E-14 | 2.13E-12 |
| LINC01213 | 1.122134 | 1.4563553 | 25.385372 | 7.03E-07 | 4.06E-06 |
| AC016705.2 | 1.1210334 | 0.1623935 | 22.276402 | 3.237E-06 | 1.587E-05 |
| TBX2 | 1.1177977 | 7.0671635 | 36.399685 | 3.573E-09 | 3.873E-08 |
| CASQ1 | 1.1165023 | 3.3244213 | 19.84111 | 1.085E-05 | 4.628E-05 |
| AC027348.1 | 1.1157013 | -1.032412 | 27.634129 | 2.355E-07 | 1.544E-06 |
| GRHL3 | 1.114939 | 6.8311651 | 31.91566 | 3.001E-08 | 2.531E-07 |
| GCNT4 | 1.1130258 | 4.3290877 | 45.218081 | 5.879E-11 | 1.098E-09 |
| AC018755.4 | 1.1129756 | 0.1066746 | 18.238046 | 2.421E-05 | 9.453E-05 |
| COL4A3 | 1.1126789 | 0.835384 | 21.745557 | 4.209E-06 | 2.011E-05 |
| AL109614.1 | 1.1125184 | -0.899223 | 46.224673 | 3.701E-11 | 7.312E-10 |
| AC106845.1 | 1.1095119 | -0.976143 | 34.500927 | 8.768E-09 | 8.568E-08 |
| SSPOP | 1.1094182 | 0.3973893 | 43.877608 | 1.091E-10 | 1.872E-09 |
| AP000350.6 | 1.109152 | 0.0357974 | 22.833034 | 2.459E-06 | 1.241E-05 |
| SEMA5A | 1.1089442 | 6.7045723 | 39.690708 | 7.625E-10 | 1.001E-08 |
| AL691432.1 | 1.1061388 | 0.4084419 | 66.430652 | 4.359E-15 | 3.799E-13 |
| TMEM132E | 1.1046334 | -0.470879 | 35.413116 | 5.693E-09 | 5.861E-08 |
| TFF3 | 1.1035184 | 3.9243581 | 11.372285 | 0.0008157 | 0.0021548 |
| AC108112.1 | 1.1026053 | -0.929133 | 22.027125 | 3.661E-06 | 1.774E-05 |
| TXK | 1.1003377 | 1.2696936 | 64.230126 | 1.143E-14 | 8.41E-13 |
| AC013394.1 | 1.0981931 | -0.310767 | 43.74007 | 1.162E-10 | 1.973E-09 |
| ADGRB1 | 1.0979979 | 0.961983 | 25.951765 | 5.333E-07 | 3.172E-06 |
| JPX | 1.0952237 | 0.6541099 | 74.302853 | 1.44E-16 | 2.278E-14 |
| CAPN9 | 1.0952052 | 1.7418572 | 15.803027 | 8.295E-05 | 0.0002809 |
| FAM3D | 1.0943091 | 3.1101985 | 14.194094 | 0.0001888 | 0.0005832 |
| OR13A1 | 1.094065 | 1.0537362 | 29.225204 | 1.091E-07 | 7.861E-07 |
| AC127070.4 | 1.0938582 | -1.338503 | 59.148328 | 1.08E-13 | 5.381E-12 |
| TEKT5 | 1.0932736 | 0.9159933 | 23.644584 | 1.649E-06 | 8.66E-06 |
| KCNN1 | 1.0923432 | 0.0098697 | 26.226849 | 4.664E-07 | 2.814E-06 |
| AP000553.2 | 1.0916748 | -1.548085 | 39.147745 | 9.828E-10 | 1.251E-08 |
| DOCK3 | 1.0914647 | 3.4882708 | 36.522702 | 3.371E-09 | 3.698E-08 |
| MYLK3 | 1.0913458 | -0.914049 | 54.622106 | 8.167E-13 | 2.864E-11 |
| RGL3 | 1.0904237 | 4.9077854 | 29.385731 | 1.01E-07 | 7.336E-07 |
| LINC02575 | 1.0894484 | 0.850919 | 12.796531 | 0.0003884 | 0.0011093 |
| OVOL1-AS1 | 1.0881274 | -0.686257 | 35.614976 | 5.174E-09 | 5.372E-08 |
| AC072052.1 | 1.0879803 | -1.212306 | 48.380867 | 1.379E-11 | 3.134E-10 |
| AP000553.7 | 1.0876795 | -1.860096 | 40.063241 | 6.408E-10 | 8.628E-09 |
| AC005972.3 | 1.0876254 | -1.08153 | 45.244854 | 5.807E-11 | 1.087E-09 |
| AC011297.1 | 1.0854143 | 0.8522145 | 10.502972 | 0.0012883 | 0.0032461 |
| KCNC3 | 1.0849322 | 3.422107 | 53.539873 | 1.329E-12 | 4.308E-11 |
| CPAMD8 | 1.0843017 | 4.0514386 | 35.4324 | 5.641E-09 | 5.814E-08 |
| B3GALT5-AS1 | 1.0835989 | 1.8105335 | 16.872315 | 4.821E-05 | 0.0001739 |
| SCIN | 1.0826376 | 4.4123233 | 29.427899 | 9.899E-08 | 7.215E-07 |
| PCAT7 | 1.08262 | 2.471872 | 44.000053 | 1.031E-10 | 1.782E-09 |
| SCGB1B2P | 1.0825022 | 0.0553807 | 46.660199 | 3.031E-11 | 6.175E-10 |
| PRH2 | 1.0796422 | -1.232531 | 55.44813 | 5.636E-13 | 2.059E-11 |
| AC129507.2 | 1.0737164 | -1.427297 | 27.847287 | 2.124E-07 | 1.412E-06 |
| TPBGL | 1.0734438 | 0.9495379 | 28.230302 | 1.764E-07 | 1.2E-06 |
| AP001574.1 | 1.0731877 | -1.496359 | 30.308723 | 6.481E-08 | 4.967E-07 |
| AC010615.1 | 1.072473 | 0.0337645 | 53.952162 | 1.104E-12 | 3.703E-11 |
| USP32P1 | 1.072165 | -0.898341 | 18.658451 | 1.96E-05 | 7.824E-05 |
| AL355377.3 | 1.0703745 | -1.174972 | 40.606948 | 4.973E-10 | 6.908E-09 |
| FER1L4 | 1.0685272 | 8.0636727 | 25.851814 | 5.599E-07 | 3.312E-06 |
| PABPC4-AS1 | 1.0678965 | -1.43531 | 52.98127 | 1.71E-12 | 5.295E-11 |
| AC008982.2 | 1.0676345 | -1.27644 | 47.39654 | 2.163E-11 | 4.632E-10 |
| AC006059.1 | 1.0629184 | 0.0224672 | 43.154197 | 1.524E-10 | 2.47E-09 |
| LINC02018 | 1.0623897 | 0.0184302 | 50.519748 | 5.21E-12 | 1.364E-10 |
| IGSF5 | 1.0613666 | -0.948535 | 23.273803 | 1.979E-06 | 1.02E-05 |
| MIR429 | 1.0611154 | -0.636743 | 29.627832 | 8.991E-08 | 6.644E-07 |
| RASL10A | 1.0602285 | 1.2982372 | 39.143023 | 9.85E-10 | 1.253E-08 |
| SEMA6A-AS1 | 1.0601972 | -0.428604 | 51.928012 | 2.752E-12 | 7.925E-11 |
| VN1R83P | 1.0574848 | -1.44974 | 44.903717 | 6.794E-11 | 1.248E-09 |
| AC025539.1 | 1.0572903 | -0.868011 | 27.606288 | 2.387E-07 | 1.563E-06 |
| PSCA | 1.0572402 | 10.125971 | 13.04196 | 0.000342 | 0.0009896 |
| KRT33B | 1.0568163 | 0.6651831 | 11.908412 | 0.0006163 | 0.0016766 |
| MTRNR2L12 | 1.0567246 | 1.1603676 | 35.081878 | 6.658E-09 | 6.698E-08 |
| TRBV30 | 1.056139 | -0.332777 | 16.304063 | 6.43E-05 | 0.0002251 |
| LINC01767 | 1.053628 | -0.712017 | 25.027949 | 8.372E-07 | 4.719E-06 |
| KRTAP5-8 | 1.0530586 | -0.568182 | 18.211609 | 2.453E-05 | 9.561E-05 |
| AC096751.1 | 1.0521824 | -0.862596 | 21.863309 | 3.97E-06 | 1.911E-05 |
| PNMA6A | 1.0516804 | -0.282789 | 20.703974 | 7.056E-06 | 3.17E-05 |
| AC008736.1 | 1.0509863 | 2.3155304 | 40.418828 | 5.428E-10 | 7.464E-09 |
| TLX2 | 1.0498619 | -0.529007 | 26.018245 | 5.163E-07 | 3.08E-06 |
| AC073957.3 | 1.0494597 | 2.3329145 | 72.659952 | 2.92E-16 | 3.971E-14 |
| SHROOM1 | 1.04911 | 5.576954 | 62.473563 | 2.477E-14 | 1.568E-12 |
| CAPN5 | 1.0486201 | 6.385446 | 44.83721 | 7.005E-11 | 1.28E-09 |
| CNGA1 | 1.0486059 | 4.0171688 | 28.966263 | 1.237E-07 | 8.762E-07 |
| SLC4A4 | 1.0485961 | 2.817491 | 18.465106 | 2.16E-05 | 8.535E-05 |
| GDA | 1.04819 | 2.8847164 | 14.893639 | 0.0001319 | 0.0004226 |
| AC114488.1 | 1.0450443 | 0.5623081 | 41.768806 | 2.896E-10 | 4.312E-09 |
| AC018665.1 | 1.0448354 | 1.3945888 | 56.914825 | 2.923E-13 | 1.221E-11 |
| AL731567.1 | 1.0433806 | 0.5610973 | 39.922918 | 6.841E-10 | 9.139E-09 |
| AC006262.1 | 1.0421983 | 1.750356 | 15.506812 | 9.646E-05 | 0.0003216 |
| AP003068.4 | 1.0419477 | -0.78469 | 33.195209 | 1.63E-08 | 1.484E-07 |
| ZNF321P | 1.041735 | 2.2027409 | 63.432473 | 1.624E-14 | 1.127E-12 |
| KCNQ1OT1 | 1.0387961 | 3.0681059 | 41.197896 | 3.777E-10 | 5.454E-09 |
| FAM47E | 1.0386197 | 0.3044747 | 65.743913 | 5.887E-15 | 4.831E-13 |
| HTRA4 | 1.036705 | 0.0157149 | 21.263062 | 5.345E-06 | 2.487E-05 |
| EHMT2-AS1 | 1.0343356 | -0.733659 | 58.177416 | 1.664E-13 | 7.594E-12 |
| COL9A3 | 1.0331071 | 2.9804598 | 26.207476 | 4.708E-07 | 2.835E-06 |
| AUXG01000058.1 | 1.0315122 | 0.8552057 | 31.770538 | 3.217E-08 | 2.682E-07 |
| VWA5B2 | 1.0303836 | 0.0890284 | 22.955192 | 2.316E-06 | 1.174E-05 |
| LIPC | 1.0301983 | 2.6262011 | 21.849569 | 3.997E-06 | 1.921E-05 |
| SNX32 | 1.030097 | -0.437632 | 57.47934 | 2.271E-13 | 9.833E-12 |
| AP000866.5 | 1.0298674 | -1.635815 | 37.10241 | 2.566E-09 | 2.917E-08 |
| AVPR1A | 1.028925 | 2.7627687 | 27.01293 | 3.183E-07 | 2.01E-06 |
| CCR7 | 1.0278687 | 4.8301545 | 17.816734 | 2.993E-05 | 0.0001142 |
| AC139887.4 | 1.027231 | -1.430614 | 55.678123 | 5.084E-13 | 1.895E-11 |
| ARSDP1 | 1.026345 | -1.14929 | 14.433861 | 0.000167 | 0.0005213 |
| AP000331.1 | 1.0260743 | -0.609167 | 20.944895 | 6.26E-06 | 2.848E-05 |
| GSTM1 | 1.0260729 | 6.5234903 | 5.4026846 | 0.0205895 | 0.0377268 |
| AL445309.1 | 1.0243383 | -1.500972 | 36.008358 | 4.297E-09 | 4.557E-08 |
| TTC6 | 1.0232356 | 1.6978414 | 29.596597 | 9.127E-08 | 6.725E-07 |
| LNC-LBCS | 1.0217525 | 0.7586504 | 23.760654 | 1.558E-06 | 8.226E-06 |
| BMX | 1.0214136 | 1.3148041 | 17.366531 | 3.755E-05 | 0.0001392 |
| AL158175.1 | 1.0207834 | 1.8739093 | 8.9710903 | 0.0029078 | 0.0066683 |
| AC100860.1 | 1.0197118 | -0.233163 | 18.878997 | 1.755E-05 | 7.072E-05 |
| KRT7-AS | 1.0193281 | 3.696947 | 27.509459 | 2.501E-07 | 1.627E-06 |
| SNORD19C | 1.0192055 | -1.085992 | 59.886438 | 7.781E-14 | 4.166E-12 |
| AL359715.1 | 1.0178101 | 1.3269615 | 43.325091 | 1.408E-10 | 2.317E-09 |
| ARL14 | 1.0170014 | 3.40927 | 18.801452 | 1.824E-05 | 7.327E-05 |
| ANXA9 | 1.0165296 | 5.1164355 | 37.833041 | 1.82E-09 | 2.155E-08 |
| CCDC169 | 1.0157661 | 1.6228352 | 37.608542 | 2.023E-09 | 2.371E-08 |
| VWA5B1 | 1.0144761 | 1.6999037 | 10.274848 | 0.0014532 | 0.0036115 |
| TNFSF15 | 1.0138909 | 3.9242007 | 42.007939 | 2.592E-10 | 3.911E-09 |
| AL359715.2 | 1.0128406 | -0.067451 | 38.192641 | 1.537E-09 | 1.852E-08 |
| AL139407.1 | 1.0128102 | -0.652678 | 57.45529 | 2.296E-13 | 9.896E-12 |
| NIPAL1 | 1.0105463 | 5.1020086 | 52.031995 | 2.625E-12 | 7.659E-11 |
| STRC | 1.0105079 | -1.267598 | 26.989115 | 3.22E-07 | 2.03E-06 |
| UGT2B7 | 1.0096111 | 2.770588 | 9.5938427 | 0.0020854 | 0.0049638 |
| TMEM191B | 1.0093483 | -0.511407 | 24.398775 | 1.139E-06 | 6.213E-06 |
| ARHGEF26-AS1 | 1.0087586 | 1.5635196 | 21.306311 | 5.232E-06 | 2.441E-05 |
| CFAP91 | 1.0084351 | 0.0419222 | 20.676438 | 7.154E-06 | 3.209E-05 |
| AC005785.2 | 1.0083024 | -1.500566 | 59.711763 | 8.408E-14 | 4.443E-12 |
| RNF157-AS1 | 1.0080285 | 0.497833 | 27.656164 | 2.33E-07 | 1.53E-06 |
| AC087857.1 | 1.0069714 | 0.0341871 | 17.021786 | 4.47E-05 | 0.0001624 |
| NPPC | 1.0069055 | -0.086454 | 12.718904 | 0.0004043 | 0.0011486 |
| AC002059.1 | 1.0067414 | -0.11172 | 30.393049 | 6.224E-08 | 4.793E-07 |
| AC012618.3 | 1.0058576 | -0.574555 | 65.893463 | 5.514E-15 | 4.605E-13 |
| AC005393.1 | 1.0055234 | -0.845067 | 42.668068 | 1.908E-10 | 2.997E-09 |
| SBK1 | 1.0054097 | 4.6887918 | 24.448364 | 1.112E-06 | 6.077E-06 |
| ZNF300 | 1.004711 | 3.5367188 | 36.690428 | 3.115E-09 | 3.446E-08 |
| MUC20P1 | 1.004374 | 2.7252865 | 23.44455 | 1.82E-06 | 9.466E-06 |
| LRFN2 | 1.0038395 | 0.7927981 | 15.905282 | 7.875E-05 | 0.0002686 |
| GATM | 1.0022655 | 5.3521312 | 26.324671 | 4.447E-07 | 2.694E-06 |
| PPARG | 1.0007886 | 7.153532 | 36.211853 | 3.904E-09 | 4.186E-08 |
| CYP4Z2P | 1.0005539 | 0.8377345 | 17.148754 | 4.192E-05 | 0.0001534 |
| NKAIN1 | 1.0003585 | 0.8428088 | 19.598863 | 1.224E-05 | 5.145E-05 |
| CCDC71L | -1.000014 | 5.1829119 | 72.604239 | 2.991E-16 | 4.041E-14 |
| MIR155HG | -1.000153 | 0.4307663 | 36.400262 | 3.572E-09 | 3.873E-08 |
| CD8B | -1.000649 | 2.1625096 | 19.266253 | 1.445E-05 | 5.957E-05 |
| ARHGAP9 | -1.003235 | 3.2173559 | 37.410842 | 2.219E-09 | 2.577E-08 |
| ICAM1 | -1.003407 | 5.7876148 | 35.426463 | 5.657E-09 | 5.828E-08 |
| CHST15 | -1.003864 | 4.7523424 | 38.406742 | 1.391E-09 | 1.696E-08 |
| PILRA | -1.00411 | 2.4602121 | 43.186588 | 1.501E-10 | 2.443E-09 |
| IGLV2-14 | -1.004681 | 4.9069933 | 12.55174 | 0.000441 | 0.0012415 |
| CFI | -1.004688 | 1.8588772 | 37.654789 | 1.979E-09 | 2.326E-08 |
| MAMLD1 | -1.005191 | 1.7389906 | 54.627152 | 8.148E-13 | 2.864E-11 |
| WT1 | -1.005474 | 0.3536285 | 11.940857 | 0.000606 | 0.0016523 |
| FMO1 | -1.005657 | 1.3800356 | 27.534951 | 2.471E-07 | 1.609E-06 |
| CCL26 | -1.00574 | -1.215023 | 25.369165 | 7.086E-07 | 4.082E-06 |
| OSMR-AS1 | -1.005811 | -0.689492 | 33.591478 | 1.35E-08 | 1.258E-07 |
| AC008760.2 | -1.006067 | -0.086367 | 21.164776 | 5.613E-06 | 2.588E-05 |
| RAB7B | -1.007614 | 3.665699 | 41.452121 | 3.356E-10 | 4.913E-09 |
| RASSF10 | -1.008472 | 2.7996306 | 24.466084 | 1.102E-06 | 6.037E-06 |
| DPP4 | -1.008473 | 3.4836897 | 20.101933 | 9.523E-06 | 4.129E-05 |
| BTK | -1.009483 | 2.4846077 | 35.764297 | 4.822E-09 | 5.061E-08 |
| CLEC2B | -1.009863 | 4.6943269 | 32.959103 | 1.824E-08 | 1.634E-07 |
| TPD52L1 | -1.010901 | 4.9531223 | 53.577322 | 1.307E-12 | 4.257E-11 |
| C5AR1 | -1.011353 | 3.8978545 | 43.495124 | 1.301E-10 | 2.165E-09 |
| CHST11 | -1.01287 | 5.3733697 | 44.174683 | 9.508E-11 | 1.667E-09 |
| THEMIS2 | -1.013587 | 4.5862423 | 56.280682 | 3.881E-13 | 1.535E-11 |
| IGSF21 | -1.014148 | 1.2523096 | 23.395934 | 1.864E-06 | 9.667E-06 |
| CDK5R1 | -1.015789 | 3.2740263 | 49.369367 | 8.79E-12 | 2.147E-10 |
| C1orf162 | -1.015839 | 3.3949284 | 53.434637 | 1.394E-12 | 4.467E-11 |
| FAS | -1.016127 | 3.8948137 | 66.245799 | 4.726E-15 | 4.048E-13 |
| TPSD1 | -1.016751 | 0.1267466 | 15.060431 | 0.0001211 | 0.0003924 |
| DPT | -1.017072 | 2.1327915 | 11.858186 | 0.0006327 | 0.0017179 |
| MOB3B | -1.017818 | 4.0756301 | 56.22947 | 3.971E-13 | 1.567E-11 |
| CD79A | -1.018826 | 3.8533287 | 13.506532 | 0.000269 | 0.0007993 |
| AL353807.5 | -1.018836 | 0.9190117 | 45.782257 | 4.535E-11 | 8.728E-10 |
| BDKRB1 | -1.019424 | 1.1406465 | 33.418437 | 1.466E-08 | 1.351E-07 |
| AKR1B15 | -1.019739 | 2.815226 | 14.549019 | 0.0001574 | 0.0004954 |
| LDHAP7 | -1.020086 | -0.474144 | 34.062911 | 1.079E-08 | 1.032E-07 |
| PCOLCE2 | -1.020499 | 2.362526 | 20.324294 | 8.524E-06 | 3.742E-05 |
| TRIM22 | -1.021076 | 5.8551685 | 53.161088 | 1.577E-12 | 4.959E-11 |
| TBX21 | -1.021178 | 0.2887792 | 30.176572 | 6.905E-08 | 5.251E-07 |
| ADM | -1.021226 | 6.201203 | 54.355165 | 9.208E-13 | 3.18E-11 |
| ITGB3 | -1.021486 | 2.7781754 | 39.474586 | 8.435E-10 | 1.093E-08 |
| LINC01358 | -1.022071 | -0.828375 | 46.624765 | 3.081E-11 | 6.257E-10 |
| ADAM19 | -1.022588 | 5.4905731 | 32.632616 | 2.131E-08 | 1.872E-07 |
| SLA2 | -1.022631 | 1.521337 | 34.562435 | 8.516E-09 | 8.364E-08 |
| IGHV4-39 | -1.022783 | 4.4097748 | 12.957589 | 0.0003573 | 0.0010283 |
| ME1 | -1.02312 | 4.5391084 | 47.703315 | 1.88E-11 | 4.08E-10 |
| SLC7A5 | -1.026917 | 7.9958182 | 47.116768 | 2.459E-11 | 5.156E-10 |
| NLRP3 | -1.02764 | 1.6750498 | 38.303491 | 1.46E-09 | 1.77E-08 |
| AC018629.1 | -1.028768 | 2.727114 | 28.847033 | 1.31E-07 | 9.215E-07 |
| LINC01186 | -1.028826 | -0.337843 | 22.260585 | 3.262E-06 | 1.598E-05 |
| IGLV6-57 | -1.028895 | 4.0282677 | 11.971307 | 0.0005964 | 0.0016287 |
| XIRP1 | -1.029266 | 0.9949287 | 15.196778 | 0.000113 | 0.0003687 |
| IGHV3-20 | -1.029527 | 1.2205295 | 10.591277 | 0.0012296 | 0.0031186 |
| AC104024.2 | -1.029698 | -0.700987 | 25.808879 | 5.718E-07 | 3.375E-06 |
| KLRC1 | -1.030176 | 0.1273321 | 19.387205 | 1.361E-05 | 5.641E-05 |
| FADS3 | -1.030611 | 4.9801807 | 61.332852 | 4.1E-14 | 2.442E-12 |
| WAS | -1.030696 | 3.5656243 | 46.897149 | 2.719E-11 | 5.625E-10 |
| SERPINB5 | -1.031054 | 7.2078894 | 25.721264 | 5.967E-07 | 3.507E-06 |
| IFI16 | -1.031203 | 7.3324566 | 52.619832 | 2.013E-12 | 6.066E-11 |
| RGS18 | -1.031528 | 0.0769317 | 40.447868 | 5.355E-10 | 7.379E-09 |
| TRIM16L | -1.03318 | 3.9680496 | 46.094515 | 3.929E-11 | 7.701E-10 |
| TNFRSF17 | -1.033412 | 0.1251151 | 16.74196 | 5.15E-05 | 0.0001842 |
| CSPG4 | -1.033826 | 5.5775289 | 43.492554 | 1.303E-10 | 2.166E-09 |
| PTAFR | -1.035254 | 4.5161592 | 67.880939 | 2.315E-15 | 2.209E-13 |
| MYO15A | -1.035963 | 1.4308215 | 27.928228 | 2.042E-07 | 1.365E-06 |
| TPRXL | -1.038322 | 2.9032634 | 17.3123 | 3.86E-05 | 0.0001425 |
| MUCL1 | -1.038381 | 1.9981861 | 8.9988333 | 0.0028649 | 0.0065798 |
| IGHV3-21 | -1.038609 | 4.2699647 | 12.968284 | 0.0003553 | 0.001023 |
| IFI27 | -1.038627 | 8.6054065 | 28.764364 | 1.363E-07 | 9.555E-07 |
| SUSD3 | -1.03863 | 2.0761485 | 41.390102 | 3.454E-10 | 5.038E-09 |
| TRABD2A | -1.040552 | 2.2993557 | 21.415075 | 4.957E-06 | 2.326E-05 |
| SCIMP | -1.041022 | 1.2237852 | 32.213921 | 2.603E-08 | 2.243E-07 |
| LINC01160 | -1.041483 | -1.156286 | 34.279553 | 9.738E-09 | 9.425E-08 |
| IGHG1 | -1.042532 | 11.197767 | 12.988196 | 0.0003517 | 0.0010141 |
| LST1 | -1.043197 | 3.0646318 | 46.176652 | 3.784E-11 | 7.446E-10 |
| AOC1 | -1.043371 | 3.4412787 | 13.640086 | 0.0002511 | 0.000752 |
| S100A10 | -1.043452 | 8.6861478 | 51.899616 | 2.787E-12 | 8.016E-11 |
| ACP7 | -1.044554 | 1.7692284 | 17.691292 | 3.188E-05 | 0.0001202 |
| MT1A | -1.044878 | 0.7480592 | 13.631959 | 0.0002522 | 0.0007546 |
| RAB31 | -1.045084 | 6.7212868 | 72.676737 | 2.899E-16 | 3.97E-14 |
| MPEG1 | -1.045805 | 4.4292384 | 36.613001 | 3.231E-09 | 3.562E-08 |
| PAQR5 | -1.046603 | 2.7247352 | 31.184147 | 4.259E-08 | 3.437E-07 |
| EBI3 | -1.049035 | 1.4728025 | 33.343199 | 1.519E-08 | 1.392E-07 |
| FAM83A-AS1 | -1.049559 | 2.224663 | 19.377586 | 1.367E-05 | 5.666E-05 |
| TRBV7-9 | -1.050462 | -1.179188 | 23.118169 | 2.137E-06 | 1.091E-05 |
| HTR1D | -1.052957 | 1.2000369 | 21.245309 | 5.393E-06 | 2.505E-05 |
| FAP | -1.054546 | 3.8608498 | 28.156833 | 1.828E-07 | 1.237E-06 |
| SEC14L2 | -1.054915 | 3.9649254 | 68.811761 | 1.544E-15 | 1.539E-13 |
| CHRNA1 | -1.055051 | 0.2521412 | 24.545195 | 1.06E-06 | 5.846E-06 |
| ZNF385B | -1.055251 | 0.2169377 | 14.811488 | 0.0001376 | 0.000439 |
| TAP2 | -1.056245 | 5.6887477 | 96.182678 | 1.491E-20 | 8.83E-18 |
| SLC7A7 | -1.056245 | 3.8816093 | 48.041817 | 1.61E-11 | 3.578E-10 |
| PTPN22 | -1.056455 | 1.713872 | 39.291443 | 9.189E-10 | 1.179E-08 |
| IRF8 | -1.057645 | 3.7563135 | 28.55964 | 1.505E-07 | 1.043E-06 |
| TLR6 | -1.05791 | 1.8154266 | 44.667187 | 7.576E-11 | 1.368E-09 |
| SLC2A12 | -1.058061 | 1.6208321 | 25.417157 | 6.922E-07 | 4.007E-06 |
| RASGRP4 | -1.059489 | 0.5653347 | 54.674403 | 7.977E-13 | 2.817E-11 |
| HLA-DRB1 | -1.061066 | 8.6613572 | 36.524358 | 3.369E-09 | 3.698E-08 |
| DSE | -1.061277 | 5.1343819 | 64.734257 | 9.163E-15 | 7.013E-13 |
| FFAR4 | -1.06249 | 0.0293776 | 25.922711 | 5.409E-07 | 3.212E-06 |
| AC010198.2 | -1.06255 | 1.0641173 | 36.367607 | 3.627E-09 | 3.923E-08 |
| CXorf21 | -1.063302 | 0.4065486 | 42.166271 | 2.408E-10 | 3.662E-09 |
| GBP1P1 | -1.063302 | 2.8197372 | 50.422284 | 5.446E-12 | 1.422E-10 |
| PRNP | -1.063995 | 7.7485368 | 62.797257 | 2.148E-14 | 1.415E-12 |
| GGTA1 | -1.06575 | 2.1264907 | 40.022063 | 6.532E-10 | 8.772E-09 |
| FZD9 | -1.066007 | -0.109895 | 22.588477 | 2.774E-06 | 1.384E-05 |
| IGKV3D-11 | -1.066074 | -0.071708 | 15.475482 | 9.801E-05 | 0.0003259 |
| ALDH1A1 | -1.066324 | 5.8768152 | 16.289215 | 6.479E-05 | 0.0002265 |
| DEFB1 | -1.066479 | 3.9531198 | 16.520364 | 5.762E-05 | 0.0002041 |
| P2RY13 | -1.066667 | 1.1845936 | 33.845413 | 1.197E-08 | 1.127E-07 |
| BLACAT1 | -1.066907 | 1.6591099 | 21.583992 | 4.559E-06 | 2.161E-05 |
| CLMP | -1.067256 | 4.5933264 | 32.765982 | 2E-08 | 1.77E-07 |
| KYNU | -1.069645 | 5.3949335 | 36.42049 | 3.538E-09 | 3.843E-08 |
| MILR1 | -1.069662 | 2.0201568 | 47.902874 | 1.716E-11 | 3.779E-10 |
| GNGT2 | -1.070124 | 0.4635113 | 64.892545 | 8.548E-15 | 6.593E-13 |
| KLRD1 | -1.070339 | 1.8219851 | 33.384983 | 1.49E-08 | 1.37E-07 |
| IGKV3-7 | -1.070458 | -0.15131 | 14.149607 | 0.0001932 | 0.0005953 |
| LILRA2 | -1.07097 | 0.0555731 | 48.492562 | 1.311E-11 | 3.009E-10 |
| IGHGP | -1.071768 | 7.6959877 | 12.513429 | 0.0004498 | 0.0012633 |
| PKDCC | -1.072092 | 3.3998927 | 21.163385 | 5.616E-06 | 2.588E-05 |
| DTX1 | -1.072966 | 2.3042268 | 30.704004 | 5.361E-08 | 4.202E-07 |
| SLC38A5 | -1.073189 | 5.1156692 | 26.179653 | 4.773E-07 | 2.869E-06 |
| RGS4 | -1.074063 | 2.7220081 | 28.588632 | 1.484E-07 | 1.031E-06 |
| KCTD14 | -1.07467 | -0.30246 | 36.043365 | 4.227E-09 | 4.497E-08 |
| CR2 | -1.074837 | 3.4288997 | 8.1764706 | 0.0044586 | 0.0097187 |
| NPBWR1 | -1.075356 | 1.6500502 | 18.541334 | 2.079E-05 | 8.239E-05 |
| SPINK5 | -1.076851 | 4.342225 | 21.12256 | 5.731E-06 | 2.632E-05 |
| DNAJC5B | -1.079321 | 0.3533809 | 18.348267 | 2.29E-05 | 8.998E-05 |
| IL18RAP | -1.079464 | 0.2454281 | 43.197896 | 1.493E-10 | 2.434E-09 |
| SASH3 | -1.079983 | 3.4250802 | 36.896758 | 2.827E-09 | 3.165E-08 |
| IFIH1 | -1.081928 | 5.0269743 | 72.364532 | 3.316E-16 | 4.392E-14 |
| SNAI2 | -1.081989 | 5.5517773 | 46.868572 | 2.755E-11 | 5.693E-10 |
| MCEMP1 | -1.082874 | -0.706927 | 32.698469 | 2.066E-08 | 1.817E-07 |
| DAPL1 | -1.082989 | 2.4519514 | 10.699298 | 0.0011616 | 0.0029632 |
| ALOX5AP | -1.083723 | 3.8051824 | 40.366447 | 5.563E-10 | 7.617E-09 |
| RPSAP52 | -1.086175 | -0.295279 | 16.438662 | 6.006E-05 | 0.0002119 |
| PDCD1 | -1.086345 | 1.5298652 | 27.493703 | 2.52E-07 | 1.638E-06 |
| TRPV2 | -1.086511 | 3.8788658 | 53.784302 | 1.191E-12 | 3.946E-11 |
| EMP1 | -1.086889 | 7.3312825 | 60.78003 | 5.236E-14 | 2.994E-12 |
| SELPLG | -1.088065 | 4.2728387 | 46.179903 | 3.778E-11 | 7.446E-10 |
| PLK2 | -1.089889 | 6.1748936 | 75.13295 | 1.009E-16 | 1.692E-14 |
| CTLA4 | -1.090765 | 1.791457 | 35.742768 | 4.871E-09 | 5.106E-08 |
| AC079949.2 | -1.092311 | 0.6197206 | 15.502709 | 9.666E-05 | 0.0003222 |
| IGHV2-5 | -1.0934 | 3.4497647 | 13.534402 | 0.0002652 | 0.0007895 |
| STAB1 | -1.093735 | 6.3065606 | 46.028309 | 4.05E-11 | 7.922E-10 |
| PLAUR | -1.093742 | 5.9277431 | 69.309713 | 1.244E-15 | 1.271E-13 |
| VMO1 | -1.094034 | 1.8890386 | 41.601267 | 3.131E-10 | 4.607E-09 |
| PDZK1 | -1.094746 | 0.5470829 | 23.907099 | 1.45E-06 | 7.711E-06 |
| CRTAM | -1.095474 | 0.2142312 | 33.625243 | 1.329E-08 | 1.241E-07 |
| HTR7 | -1.096467 | 2.2865341 | 21.75795 | 4.183E-06 | 2.001E-05 |
| IL15 | -1.096468 | 2.4270544 | 76.236182 | 6.291E-17 | 1.218E-14 |
| IRX3 | -1.097128 | 4.498974 | 24.912792 | 8.857E-07 | 4.972E-06 |
| IL2RG | -1.098024 | 3.5830923 | 33.613073 | 1.336E-08 | 1.247E-07 |
| C12orf54 | -1.098496 | 0.4758375 | 20.352543 | 8.405E-06 | 3.696E-05 |
| TMIGD3 | -1.098921 | 1.0115943 | 45.744466 | 4.615E-11 | 8.855E-10 |
| AC012236.1 | -1.099161 | -0.04243 | 25.195587 | 7.713E-07 | 4.401E-06 |
| TNFRSF9 | -1.100056 | 1.7302752 | 37.636367 | 1.996E-09 | 2.344E-08 |
| SLCO2B1 | -1.100116 | 4.9490086 | 37.873262 | 1.786E-09 | 2.122E-08 |
| B4GALNT1 | -1.10114 | 2.4603616 | 25.44871 | 6.816E-07 | 3.954E-06 |
| ZNF114 | -1.101877 | 2.1611009 | 21.569317 | 4.592E-06 | 2.176E-05 |
| LINC01614 | -1.102589 | 0.577564 | 18.084133 | 2.616E-05 | 0.0001014 |
| APCDD1L-DT | -1.103048 | 0.9785868 | 13.897504 | 0.0002199 | 0.0006683 |
| IGHG4 | -1.103065 | 7.9865636 | 12.7887 | 0.00039 | 0.0011128 |
| AC004585.1 | -1.104266 | -0.098333 | 43.465268 | 1.32E-10 | 2.192E-09 |
| TNFAIP8L2 | -1.10468 | 1.8299166 | 50.758002 | 4.676E-12 | 1.241E-10 |
| CXCL2 | -1.104707 | 3.2290106 | 23.540273 | 1.736E-06 | 9.073E-06 |
| HLA-DQA1 | -1.104918 | 6.7010292 | 32.178454 | 2.647E-08 | 2.273E-07 |
| LAPTM5 | -1.104937 | 6.4607495 | 55.619169 | 5.22E-13 | 1.928E-11 |
| LINC00519 | -1.106256 | 0.4085234 | 31.937586 | 2.97E-08 | 2.51E-07 |
| S100B | -1.106377 | 2.1058564 | 36.996383 | 2.697E-09 | 3.045E-08 |
| TUBA4A | -1.106616 | 6.5532891 | 64.168257 | 1.175E-14 | 8.568E-13 |
| RAET1L | -1.108672 | 0.8000826 | 17.294832 | 3.894E-05 | 0.0001437 |
| IGHV1-69 | -1.109261 | 1.8867516 | 11.684486 | 0.0006928 | 0.0018654 |
| IL7R | -1.109473 | 4.535687 | 33.643778 | 1.317E-08 | 1.232E-07 |
| IFIT3 | -1.109521 | 5.9645414 | 41.553107 | 3.202E-10 | 4.698E-09 |
| SERPINE2 | -1.112123 | 5.3195082 | 29.305398 | 1.05E-07 | 7.596E-07 |
| ARSJ | -1.113866 | 2.4857297 | 34.120769 | 1.05E-08 | 1.008E-07 |
| TG | -1.115633 | 3.2056217 | 14.287074 | 0.00018 | 0.0005581 |
| AC011374.1 | -1.118571 | 0.8590889 | 15.209505 | 0.0001123 | 0.0003666 |
| FOSL1 | -1.118885 | 5.4951627 | 50.944219 | 4.297E-12 | 1.161E-10 |
| TNIP3 | -1.119245 | 0.519056 | 19.922448 | 1.041E-05 | 4.463E-05 |
| KLHDC8A | -1.119824 | 0.1706127 | 32.246811 | 2.562E-08 | 2.213E-07 |
| CD53 | -1.122475 | 4.9422343 | 42.247666 | 2.319E-10 | 3.539E-09 |
| TRGC2 | -1.122902 | -0.124812 | 30.404303 | 6.19E-08 | 4.77E-07 |
| CD8A | -1.122999 | 3.5693205 | 32.479478 | 2.293E-08 | 1.999E-07 |
| HAPLN3 | -1.123835 | 4.7171579 | 52.497951 | 2.127E-12 | 6.371E-11 |
| CLEC18B | -1.125762 | -0.499574 | 26.998132 | 3.206E-07 | 2.022E-06 |
| EVA1A | -1.125941 | 2.6334552 | 29.801667 | 8.269E-08 | 6.17E-07 |
| TBXAS1 | -1.126706 | 3.615443 | 49.577806 | 7.994E-12 | 1.977E-10 |
| CD300A | -1.127034 | 3.1334441 | 65.902016 | 5.493E-15 | 4.605E-13 |
| SENCR | -1.127881 | -0.49507 | 50.61892 | 4.98E-12 | 1.314E-10 |
| GUCY2C | -1.12853 | -0.107627 | 28.671718 | 1.426E-07 | 9.94E-07 |
| SCEL | -1.129283 | 3.6163332 | 14.445213 | 0.000166 | 0.0005187 |
| CD300LF | -1.130399 | 1.3851918 | 49.224769 | 9.388E-12 | 2.263E-10 |
| RNASE6 | -1.13052 | 3.255154 | 48.291354 | 1.437E-11 | 3.236E-10 |
| WNT3A | -1.130708 | -0.08813 | 18.334952 | 2.306E-05 | 9.055E-05 |
| CD180 | -1.13092 | 2.0627947 | 39.467731 | 8.462E-10 | 1.096E-08 |
| SH3GL3 | -1.130987 | 0.219001 | 14.255253 | 0.000183 | 0.0005664 |
| IGLC2 | -1.131149 | 7.013742 | 18.218594 | 2.445E-05 | 9.539E-05 |
| HCST | -1.132142 | -0.04853 | 41.073825 | 4.001E-10 | 5.716E-09 |
| C6orf141 | -1.133979 | 2.5282602 | 26.692734 | 3.718E-07 | 2.302E-06 |
| CD70 | -1.134141 | 1.2842645 | 19.390784 | 1.358E-05 | 5.635E-05 |
| IGLV3-1 | -1.135418 | 5.2242649 | 15.675075 | 8.854E-05 | 0.0002979 |
| LILRB3 | -1.135457 | 2.5009143 | 55.788556 | 4.838E-13 | 1.831E-11 |
| IGKV1-6 | -1.136144 | 2.4969327 | 15.69612 | 8.759E-05 | 0.000295 |
| NCKAP1L | -1.136572 | 4.5551275 | 41.808636 | 2.843E-10 | 4.246E-09 |
| TNF | -1.137466 | 1.1120335 | 28.941218 | 1.252E-07 | 8.859E-07 |
| ECM1 | -1.137814 | 5.6061561 | 51.669016 | 3.094E-12 | 8.772E-11 |
| WTAPP1 | -1.137895 | -1.012399 | 34.082449 | 1.069E-08 | 1.023E-07 |
| KLK6 | -1.138022 | 3.9272962 | 7.9518348 | 0.005035 | 0.0108239 |
| CD33 | -1.139519 | 1.2580751 | 47.521654 | 2.043E-11 | 4.407E-10 |
| SOX8 | -1.141383 | 0.2024603 | 29.098246 | 1.16E-07 | 8.277E-07 |
| OSGIN1 | -1.141538 | 3.8225625 | 46.593157 | 3.126E-11 | 6.324E-10 |
| TLR2 | -1.142225 | 4.3521284 | 70.180808 | 8.524E-16 | 9.325E-14 |
| FST | -1.14341 | 5.303614 | 31.104117 | 4.425E-08 | 3.556E-07 |
| LINC01094 | -1.143644 | 1.2113472 | 48.583573 | 1.258E-11 | 2.912E-10 |
| LAIR1 | -1.144126 | 4.2882238 | 50.391808 | 5.521E-12 | 1.44E-10 |
| SIT1 | -1.145882 | 1.4654236 | 31.113362 | 4.406E-08 | 3.543E-07 |
| VEGFC | -1.146039 | 4.2038193 | 60.651977 | 5.542E-14 | 3.151E-12 |
| PLAAT4 | -1.14611 | 6.0954896 | 33.241931 | 1.594E-08 | 1.456E-07 |
| AXL | -1.147107 | 5.7458712 | 51.508661 | 3.327E-12 | 9.354E-11 |
| SRPX | -1.147428 | 4.1189674 | 34.009299 | 1.107E-08 | 1.053E-07 |
| IL1RAP | -1.147676 | 4.82468 | 66.595188 | 4.057E-15 | 3.582E-13 |
| KRT15 | -1.148143 | 7.9778211 | 19.086023 | 1.582E-05 | 6.462E-05 |
| FOXD1 | -1.14893 | 1.0698519 | 20.608972 | 7.398E-06 | 3.304E-05 |
| TRDC | -1.149149 | 0.0212897 | 27.233068 | 2.86E-07 | 1.828E-06 |
| LUCAT1 | -1.149381 | 1.4623947 | 29.708401 | 8.649E-08 | 6.42E-07 |
| OXTR | -1.150472 | 2.1963148 | 28.21718 | 1.776E-07 | 1.206E-06 |
| LILRA1 | -1.150958 | -0.611867 | 50.209132 | 5.999E-12 | 1.55E-10 |
| FMN1 | -1.15168 | 2.7107711 | 51.946741 | 2.729E-12 | 7.875E-11 |
| NTRK2 | -1.152025 | 4.0525494 | 15.109835 | 0.0001181 | 0.0003836 |
| ZBED2 | -1.152218 | 2.9328856 | 19.409779 | 1.345E-05 | 5.597E-05 |
| IGHV4-55 | -1.152479 | -0.021984 | 17.898641 | 2.872E-05 | 0.0001101 |
| ATP1A2 | -1.153557 | 2.4977143 | 14.295204 | 0.0001793 | 0.0005561 |
| TAP1 | -1.153901 | 7.3642436 | 62.093817 | 2.929E-14 | 1.809E-12 |
| ADRB2 | -1.155024 | 2.3048881 | 44.395394 | 8.588E-11 | 1.526E-09 |
| SLC15A3 | -1.155091 | 5.0839987 | 70.478478 | 7.493E-16 | 8.426E-14 |
| NT5E | -1.155681 | 5.3367871 | 33.919579 | 1.155E-08 | 1.093E-07 |
| MALL | -1.160263 | 2.9879972 | 34.058733 | 1.081E-08 | 1.033E-07 |
| CCR5 | -1.16039 | 2.6923182 | 42.916842 | 1.701E-10 | 2.711E-09 |
| LAT2 | -1.162119 | 3.3761905 | 71.616448 | 4.58E-16 | 5.727E-14 |
| ABLIM2 | -1.162798 | 1.8779593 | 51.32737 | 3.612E-12 | 1E-10 |
| MMP10 | -1.163667 | 4.6650192 | 14.663954 | 0.0001484 | 0.0004697 |
| RPL29P19 | -1.163965 | 1.0152238 | 27.632211 | 2.357E-07 | 1.545E-06 |
| ADGRG5 | -1.16573 | 1.3873168 | 37.208919 | 2.441E-09 | 2.799E-08 |
| FABP5P7 | -1.166053 | 0.8071763 | 30.354575 | 6.34E-08 | 4.872E-07 |
| CCL18 | -1.167807 | 4.8906052 | 20.325694 | 8.518E-06 | 3.741E-05 |
| AC133785.1 | -1.168503 | -1.394951 | 26.857223 | 3.433E-07 | 2.152E-06 |
| SAMD9 | -1.16993 | 5.7855583 | 49.761 | 7.355E-12 | 1.839E-10 |
| NFAM1 | -1.170067 | 2.9641541 | 53.127595 | 1.601E-12 | 5.011E-11 |
| CD7 | -1.170752 | 3.5329692 | 42.232078 | 2.336E-10 | 3.562E-09 |
| HLA-DQB1 | -1.171066 | 6.6254191 | 38.941525 | 1.082E-09 | 1.361E-08 |
| COMP | -1.171845 | 6.4761142 | 15.090122 | 0.0001193 | 0.0003872 |
| PI3 | -1.172003 | 8.1880146 | 8.2500119 | 0.004285 | 0.00939 |
| RAB38 | -1.172765 | 4.6454666 | 45.80037 | 4.498E-11 | 8.664E-10 |
| IGKV1D-8 | -1.173364 | 0.2061283 | 16.256377 | 6.588E-05 | 0.0002299 |
| FCGR1CP | -1.173475 | -1.210213 | 35.6322 | 5.133E-09 | 5.331E-08 |
| NFE2 | -1.173859 | 0.5211748 | 29.21766 | 1.095E-07 | 7.884E-07 |
| IL32 | -1.177648 | 6.0457427 | 46.857195 | 2.769E-11 | 5.717E-10 |
| MAF | -1.180812 | 5.5199001 | 73.286343 | 2.23E-16 | 3.138E-14 |
| PCSK1 | -1.180961 | 1.2800252 | 23.635512 | 1.657E-06 | 8.692E-06 |
| KRTDAP | -1.181568 | 5.1446849 | 8.3582424 | 0.0040419 | 0.0089303 |
| IL36RN | -1.183259 | 2.5670344 | 14.824061 | 0.0001367 | 0.0004366 |
| CACNA2D4 | -1.18497 | 1.551173 | 70.95725 | 6.09E-16 | 7.212E-14 |
| MUC16 | -1.18885 | 5.0556866 | 10.345211 | 0.0014002 | 0.0034935 |
| TNFSF10 | -1.189338 | 7.5078875 | 60.905358 | 4.953E-14 | 2.874E-12 |
| MRO | -1.189363 | -0.344381 | 36.146934 | 4.025E-09 | 4.312E-08 |
| TNFSF13B | -1.190123 | 2.9642122 | 54.638429 | 8.107E-13 | 2.858E-11 |
| IGHV5-51 | -1.19033 | 4.6164204 | 16.807614 | 4.982E-05 | 0.0001788 |
| FCGR2A | -1.191452 | 5.0707678 | 51.334541 | 3.6E-12 | 9.986E-11 |
| SCO2 | -1.192123 | -0.658031 | 53.885449 | 1.138E-12 | 3.797E-11 |
| ANGPTL1 | -1.192218 | 1.8806334 | 22.380214 | 3.075E-06 | 1.516E-05 |
| IGLV1-40 | -1.193326 | 4.9626022 | 17.216743 | 4.05E-05 | 0.0001487 |
| STAP1 | -1.195273 | 0.3460676 | 25.507237 | 6.624E-07 | 3.854E-06 |
| KRT10 | -1.196286 | 6.9709816 | 48.96166 | 1.058E-11 | 2.504E-10 |
| UNC5A | -1.197267 | -0.623195 | 30.533894 | 5.817E-08 | 4.521E-07 |
| CORO6 | -1.197453 | 2.7025607 | 38.674922 | 1.226E-09 | 1.515E-08 |
| SIRPA | -1.198017 | 5.5639169 | 76.434148 | 5.78E-17 | 1.145E-14 |
| CYBB | -1.199213 | 5.2859104 | 38.922626 | 1.092E-09 | 1.369E-08 |
| SOX7 | -1.19946 | 3.282331 | 46.776508 | 2.874E-11 | 5.902E-10 |
| RHOD | -1.20229 | 5.639167 | 62.794322 | 2.15E-14 | 1.415E-12 |
| MYO1G | -1.204176 | 3.5623778 | 55.773022 | 4.872E-13 | 1.838E-11 |
| ENKUR | -1.204242 | -0.64583 | 52.004093 | 2.659E-12 | 7.745E-11 |
| FBXO2 | -1.204759 | 3.9679043 | 34.653164 | 8.157E-09 | 8.045E-08 |
| HAVCR2 | -1.205433 | 3.6878663 | 57.78056 | 1.986E-13 | 8.783E-12 |
| RUNX3 | -1.206149 | 3.7302057 | 51.993932 | 2.671E-12 | 7.77E-11 |
| PELATON | -1.207116 | 1.9439108 | 55.74412 | 4.936E-13 | 1.854E-11 |
| CSF1 | -1.207319 | 5.6229254 | 75.147795 | 1.002E-16 | 1.692E-14 |
| GBP1 | -1.207527 | 6.8724714 | 55.582058 | 5.308E-13 | 1.957E-11 |
| VNN2 | -1.208351 | 1.0963084 | 37.6912 | 1.945E-09 | 2.29E-08 |
| CLEC4E | -1.20863 | 1.0613283 | 34.019858 | 1.102E-08 | 1.049E-07 |
| PIK3AP1 | -1.208825 | 3.746122 | 50.840727 | 4.504E-12 | 1.21E-10 |
| TM4SF19-AS1 | -1.20968 | -0.852768 | 49.59475 | 7.933E-12 | 1.967E-10 |
| IGKV4-1 | -1.210571 | 5.8987148 | 16.923415 | 4.698E-05 | 0.0001698 |
| CASP1 | -1.21082 | 4.9018857 | 70.831704 | 6.43E-16 | 7.569E-14 |
| FOXL1 | -1.211727 | 3.1281511 | 34.944736 | 7.105E-09 | 7.087E-08 |
| ABCA12 | -1.212059 | 3.4912715 | 28.444686 | 1.591E-07 | 1.093E-06 |
| CDC25B | -1.213192 | 6.7135595 | 110.20291 | 5.176E-23 | 6.512E-20 |
| CCL4L2 | -1.213474 | 2.0406336 | 36.677504 | 3.134E-09 | 3.465E-08 |
| ADAMDEC1 | -1.215327 | 2.7745636 | 27.253997 | 2.831E-07 | 1.814E-06 |
| CD1C | -1.215838 | 1.1891856 | 31.987258 | 2.9E-08 | 2.457E-07 |
| CD44 | -1.216622 | 8.7567516 | 66.569714 | 4.102E-15 | 3.606E-13 |
| FCGR3A | -1.216763 | 5.8874332 | 36.869672 | 2.863E-09 | 3.2E-08 |
| AC099066.2 | -1.217122 | -0.054615 | 34.524196 | 8.671E-09 | 8.498E-08 |
| SPI1 | -1.219562 | 4.4751731 | 58.323094 | 1.559E-13 | 7.232E-12 |
| MRC1 | -1.219804 | 4.5648066 | 35.610471 | 5.185E-09 | 5.38E-08 |
| LAMB3 | -1.220904 | 8.5632536 | 47.207173 | 2.359E-11 | 4.988E-10 |
| ISG20 | -1.221506 | 4.5073652 | 71.814996 | 4.204E-16 | 5.356E-14 |
| SIGLEC7 | -1.222227 | 0.0224527 | 48.674373 | 1.207E-11 | 2.818E-10 |
| IGKV3-11 | -1.222306 | 4.9688584 | 17.651485 | 3.253E-05 | 0.0001224 |
| KRT79 | -1.222395 | 3.1763864 | 11.40383 | 0.0008023 | 0.0021237 |
| HCK | -1.222529 | 4.1245228 | 51.359395 | 3.56E-12 | 9.898E-11 |
| LINC01303 | -1.223715 | -0.976095 | 36.440548 | 3.505E-09 | 3.815E-08 |
| CAV1 | -1.224598 | 7.5817679 | 48.307954 | 1.426E-11 | 3.215E-10 |
| EDIL3-DT | -1.225204 | -0.544782 | 47.052683 | 2.532E-11 | 5.293E-10 |
| FERMT3 | -1.225482 | 4.6322967 | 65.982359 | 5.303E-15 | 4.504E-13 |
| IFITM1 | -1.225906 | 5.9120027 | 53.657863 | 1.26E-12 | 4.132E-11 |
| NRG1 | -1.226821 | 3.5494345 | 20.424944 | 8.107E-06 | 3.578E-05 |
| S100A9 | -1.226899 | 10.470731 | 20.839415 | 6.597E-06 | 2.987E-05 |
| FCGR1B | -1.227269 | -0.401982 | 39.13637 | 9.881E-10 | 1.256E-08 |
| IGLV1-36 | -1.23025 | 1.7099247 | 13.283932 | 0.0003018 | 0.0008847 |
| IGHV3-73 | -1.231639 | 2.1164106 | 15.806083 | 8.282E-05 | 0.0002805 |
| IFITM3 | -1.232099 | 8.8391007 | 78.147812 | 2.783E-17 | 5.897E-15 |
| MMP12 | -1.232154 | 5.5164995 | 21.500622 | 4.751E-06 | 2.242E-05 |
| DUSP7 | -1.23632 | 6.2194251 | 103.63161 | 7.213E-22 | 6.914E-19 |
| CSF2 | -1.236446 | 0.468051 | 23.989669 | 1.392E-06 | 7.428E-06 |
| AKR1C1 | -1.236639 | 6.8969452 | 23.008205 | 2.256E-06 | 1.147E-05 |
| IGF2BP2 | -1.237086 | 5.0573309 | 31.788632 | 3.189E-08 | 2.663E-07 |
| SLC16A1 | -1.237121 | 6.0849041 | 39.932128 | 6.812E-10 | 9.105E-09 |
| ITGB2 | -1.237438 | 6.1396057 | 49.123578 | 9.831E-12 | 2.356E-10 |
| SECTM1 | -1.238192 | 5.1958029 | 42.684988 | 1.894E-10 | 2.98E-09 |
| KREMEN2 | -1.238806 | 1.7727229 | 42.401489 | 2.159E-10 | 3.328E-09 |
| GJB2 | -1.241617 | 8.2637567 | 31.309852 | 4.01E-08 | 3.255E-07 |
| PITX1 | -1.243255 | 5.8757738 | 59.051948 | 1.127E-13 | 5.562E-12 |
| MGAM | -1.243593 | 1.7184303 | 20.079704 | 9.629E-06 | 4.167E-05 |
| MS4A6A | -1.244665 | 4.92327 | 55.823046 | 4.764E-13 | 1.813E-11 |
| CD86 | -1.244861 | 3.5003239 | 69.750462 | 1.027E-15 | 1.088E-13 |
| SNORD3A | -1.24496 | 4.066757 | 22.423663 | 3.01E-06 | 1.487E-05 |
| ADGRE1 | -1.246855 | 1.5360222 | 24.787916 | 9.415E-07 | 5.25E-06 |
| F13A1 | -1.248379 | 5.4225366 | 28.222471 | 1.771E-07 | 1.203E-06 |
| CYTH4 | -1.249986 | 3.8944159 | 56.548725 | 3.443E-13 | 1.4E-11 |
| SLAMF8 | -1.251366 | 1.6758602 | 39.683159 | 7.652E-10 | 1.003E-08 |
| OSCAR | -1.251455 | 2.3675978 | 61.052815 | 4.64E-14 | 2.747E-12 |
| TYROBP | -1.251514 | 5.3953705 | 58.733283 | 1.299E-13 | 6.226E-12 |
| CD80 | -1.251546 | 0.174 | 46.525213 | 3.224E-11 | 6.497E-10 |
| FKBP5 | -1.2529 | 5.9053814 | 78.519165 | 2.376E-17 | 5.143E-15 |
| ANKRD29 | -1.256032 | 1.6851536 | 37.447796 | 2.181E-09 | 2.538E-08 |
| IL21R | -1.256922 | 1.8832344 | 39.783242 | 7.303E-10 | 9.641E-09 |
| CCDC26 | -1.259241 | -1.229202 | 43.041576 | 1.605E-10 | 2.587E-09 |
| IL22RA2 | -1.259555 | -0.649593 | 24.186081 | 1.264E-06 | 6.812E-06 |
| EMP3 | -1.261781 | 5.1671878 | 60.162091 | 6.885E-14 | 3.756E-12 |
| KRT17P1 | -1.263763 | -1.011551 | 35.172235 | 6.38E-09 | 6.44E-08 |
| TNFSF9 | -1.265281 | 1.9210106 | 41.164232 | 3.836E-10 | 5.52E-09 |
| IGKV1-17 | -1.265479 | 2.9195709 | 17.470963 | 3.563E-05 | 0.0001328 |
| SRGN | -1.268619 | 6.4210774 | 63.821955 | 1.368E-14 | 9.629E-13 |
| FASLG | -1.268737 | -0.01994 | 36.452628 | 3.485E-09 | 3.796E-08 |
| AOAH | -1.271224 | 2.7723925 | 44.211083 | 9.35E-11 | 1.647E-09 |
| TNFRSF18 | -1.272512 | 2.9758515 | 53.035276 | 1.669E-12 | 5.192E-11 |
| SIGLEC9 | -1.272791 | 1.2588135 | 60.924652 | 4.911E-14 | 2.857E-12 |
| LINC01871 | -1.273763 | -0.141589 | 36.358731 | 3.642E-09 | 3.931E-08 |
| LINC02577 | -1.274074 | 1.4627333 | 24.470251 | 1.1E-06 | 6.028E-06 |
| SLC7A11 | -1.27496 | 5.2391753 | 23.728454 | 1.583E-06 | 8.341E-06 |
| HLA-F | -1.275042 | 6.6034335 | 68.744391 | 1.59E-15 | 1.577E-13 |
| MT1E | -1.275523 | 5.1560373 | 36.865364 | 2.869E-09 | 3.204E-08 |
| TNFAIP6 | -1.276622 | 2.6218519 | 44.451696 | 8.368E-11 | 1.492E-09 |
| HSPA6 | -1.276652 | 4.8086509 | 36.324537 | 3.702E-09 | 3.985E-08 |
| BATF2 | -1.279854 | 3.787833 | 45.694461 | 4.722E-11 | 9.035E-10 |
| TUBB6 | -1.281155 | 6.729387 | 58.294794 | 1.579E-13 | 7.276E-12 |
| CD79B | -1.283034 | 2.2700519 | 28.759519 | 1.366E-07 | 9.571E-07 |
| CFAP57 | -1.283126 | 0.3648745 | 49.165591 | 9.645E-12 | 2.32E-10 |
| NHLH2 | -1.284625 | 0.903457 | 19.735131 | 1.143E-05 | 4.853E-05 |
| C3AR1 | -1.286576 | 3.4452139 | 53.441036 | 1.39E-12 | 4.467E-11 |
| MS4A4A | -1.288301 | 3.5498393 | 50.674893 | 4.855E-12 | 1.283E-10 |
| TMEM63C | -1.288784 | 0.8001156 | 27.888792 | 2.081E-07 | 1.388E-06 |
| POM121L9P | -1.289859 | -0.837029 | 44.190756 | 9.438E-11 | 1.657E-09 |
| LINP1 | -1.290412 | 0.6118116 | 21.153896 | 5.643E-06 | 2.598E-05 |
| DTHD1 | -1.291868 | -0.634564 | 32.957801 | 1.825E-08 | 1.634E-07 |
| LINC02195 | -1.292032 | -1.051676 | 35.706474 | 4.956E-09 | 5.177E-08 |
| ZNF683 | -1.292618 | 1.736077 | 27.568961 | 2.43E-07 | 1.586E-06 |
| TREM1 | -1.292899 | 3.398753 | 34.08991 | 1.066E-08 | 1.02E-07 |
| OR2I1P | -1.294883 | 4.2647161 | 23.387185 | 1.872E-06 | 9.702E-06 |
| IGKV3-15 | -1.295716 | 4.4922475 | 20.631993 | 7.313E-06 | 3.27E-05 |
| RASSF9 | -1.296696 | 1.4664759 | 32.556009 | 2.211E-08 | 1.937E-07 |
| FPR3 | -1.297134 | 4.0848378 | 50.023497 | 6.527E-12 | 1.67E-10 |
| ACY3 | -1.297763 | 0.4489814 | 49.319493 | 8.992E-12 | 2.181E-10 |
| ITM2A | -1.297946 | 3.7668502 | 45.452741 | 5.277E-11 | 1.001E-09 |
| OSMR | -1.307285 | 6.0337156 | 71.450095 | 4.921E-16 | 6.078E-14 |
| CFAP251 | -1.309117 | 3.6967172 | 42.535858 | 2.029E-10 | 3.149E-09 |
| AIM2 | -1.309765 | 4.6337418 | 30.047071 | 7.349E-08 | 5.559E-07 |
| MANCR | -1.30977 | 0.7055018 | 21.163314 | 5.617E-06 | 2.588E-05 |
| IGLV9-49 | -1.310245 | 1.1456701 | 16.462225 | 5.934E-05 | 0.0002096 |
| NCF2 | -1.311295 | 3.9828836 | 72.512955 | 3.111E-16 | 4.175E-14 |
| SLAMF9 | -1.311365 | -0.40576 | 34.632676 | 8.237E-09 | 8.12E-08 |
| LCN2 | -1.311869 | 7.8201589 | 20.633843 | 7.307E-06 | 3.268E-05 |
| GPR82 | -1.312068 | -0.21763 | 44.663999 | 7.588E-11 | 1.369E-09 |
| KCNJ12 | -1.312623 | 0.443076 | 34.038721 | 1.092E-08 | 1.043E-07 |
| AIF1 | -1.312729 | 4.4501693 | 60.973969 | 4.805E-14 | 2.828E-12 |
| MSN | -1.314042 | 8.2565541 | 96.384646 | 1.373E-20 | 8.375E-18 |
| XDH | -1.314528 | 4.1246639 | 58.012214 | 1.791E-13 | 8.047E-12 |
| IGHV4-59 | -1.315302 | 3.7828576 | 21.172844 | 5.59E-06 | 2.578E-05 |
| SLAMF1 | -1.31685 | 1.5789393 | 43.399812 | 1.36E-10 | 2.249E-09 |
| ITGAM | -1.321715 | 3.6875849 | 55.81125 | 4.789E-13 | 1.816E-11 |
| RNF217-AS1 | -1.322647 | -0.03952 | 45.674381 | 4.766E-11 | 9.093E-10 |
| PLEK | -1.322882 | 4.2276166 | 51.725817 | 3.016E-12 | 8.623E-11 |
| AL356215.1 | -1.323137 | -1.442438 | 52.26812 | 2.36E-12 | 7.026E-11 |
| DOK2 | -1.325852 | 3.2451337 | 60.12946 | 6.986E-14 | 3.801E-12 |
| SFN | -1.326982 | 9.8004326 | 78.571896 | 2.323E-17 | 5.084E-15 |
| LILRB1 | -1.329929 | 2.6424612 | 60.018286 | 7.339E-14 | 3.971E-12 |
| TNC | -1.33051 | 7.4623082 | 38.490061 | 1.337E-09 | 1.638E-08 |
| FAM20A | -1.331401 | 3.7431702 | 54.820725 | 7.469E-13 | 2.666E-11 |
| TLR8 | -1.331688 | 1.2122188 | 38.347552 | 1.43E-09 | 1.737E-08 |
| POU2AF1 | -1.331998 | 3.7200687 | 36.277549 | 3.785E-09 | 4.066E-08 |
| PDCD1LG2 | -1.334664 | 2.3032557 | 41.709494 | 2.977E-10 | 4.423E-09 |
| CXCL1 | -1.335474 | 5.2110106 | 29.809595 | 8.238E-08 | 6.149E-07 |
| KCNK12 | -1.338396 | 0.3864812 | 39.753973 | 7.403E-10 | 9.753E-09 |
| CD274 | -1.340045 | 3.7401811 | 47.983377 | 1.654E-11 | 3.667E-10 |
| GFI1 | -1.340108 | 2.027794 | 67.755109 | 2.446E-15 | 2.279E-13 |
| PSMB9 | -1.34199 | 6.3444352 | 65.834707 | 5.657E-15 | 4.706E-13 |
| CD38 | -1.343875 | 3.0701234 | 42.601597 | 1.968E-10 | 3.071E-09 |
| KRT17 | -1.344719 | 11.449187 | 39.856216 | 7.058E-10 | 9.359E-09 |
| NNMT | -1.345298 | 6.7659579 | 45.279069 | 5.716E-11 | 1.075E-09 |
| LILRB4 | -1.346247 | 4.0191478 | 44.32335 | 8.878E-11 | 1.575E-09 |
| CSF1R | -1.348437 | 5.5644716 | 59.482374 | 9.31E-14 | 4.772E-12 |
| TGFBI | -1.348851 | 8.5690951 | 45.245651 | 5.804E-11 | 1.087E-09 |
| FCER1G | -1.350349 | 4.9745639 | 65.607845 | 6.248E-15 | 5.071E-13 |
| CEACAM4 | -1.350945 | -0.488032 | 46.998495 | 2.596E-11 | 5.398E-10 |
| LRRC25 | -1.351222 | 2.5260472 | 67.691701 | 2.514E-15 | 2.332E-13 |
| ADGRG3 | -1.351647 | 0.5648978 | 53.59873 | 1.294E-12 | 4.223E-11 |
| SH3RF2 | -1.352224 | 3.6862575 | 54.123507 | 1.022E-12 | 3.463E-11 |
| NCF1 | -1.354133 | 1.6789077 | 49.445741 | 8.489E-12 | 2.081E-10 |
| SERPINB2 | -1.357677 | 4.0704206 | 16.341166 | 6.31E-05 | 0.0002214 |
| LINC02345 | -1.358544 | -0.720378 | 46.768502 | 2.884E-11 | 5.918E-10 |
| MN1 | -1.359419 | 2.8084858 | 53.876037 | 1.142E-12 | 3.807E-11 |
| TOX2 | -1.361096 | 3.0196673 | 64.048368 | 1.238E-14 | 8.999E-13 |
| LINC01711 | -1.361685 | -0.486093 | 29.10923 | 1.154E-07 | 8.242E-07 |
| KLHL4 | -1.361702 | 0.0135219 | 34.509665 | 8.731E-09 | 8.536E-08 |
| SLC47A1 | -1.362648 | 1.1806654 | 41.218527 | 3.741E-10 | 5.409E-09 |
| IGHV3-7 | -1.367056 | 3.9435607 | 22.802424 | 2.497E-06 | 1.258E-05 |
| NCF1B | -1.367617 | 0.2337294 | 43.843102 | 1.108E-10 | 1.897E-09 |
| IL12RB1 | -1.368681 | 2.4117075 | 58.969995 | 1.169E-13 | 5.726E-12 |
| TLL2 | -1.369149 | 1.3389868 | 45.676697 | 4.761E-11 | 9.092E-10 |
| LILRB5 | -1.372391 | 2.4507063 | 47.724617 | 1.862E-11 | 4.047E-10 |
| IL2RA | -1.374927 | 2.7477241 | 53.89647 | 1.132E-12 | 3.785E-11 |
| CCL3 | -1.375352 | 0.2996152 | 52.69797 | 1.943E-12 | 5.873E-11 |
| DSP | -1.376259 | 9.3768458 | 60.49425 | 5.943E-14 | 3.332E-12 |
| FCGR1A | -1.37627 | 1.914242 | 57.524637 | 2.226E-13 | 9.678E-12 |
| FAM83A | -1.380196 | 7.1318714 | 32.97967 | 1.807E-08 | 1.62E-07 |
| LYVE1 | -1.38043 | 2.874427 | 36.417431 | 3.543E-09 | 3.846E-08 |
| CCL4 | -1.380528 | 3.0868226 | 52.120576 | 2.522E-12 | 7.412E-11 |
| FPR1 | -1.380771 | 2.9159909 | 51.268539 | 3.709E-12 | 1.023E-10 |
| LILRA6 | -1.381128 | 1.1969903 | 61.946636 | 3.126E-14 | 1.901E-12 |
| CXCL9 | -1.381399 | 6.1277827 | 27.101121 | 3.049E-07 | 1.935E-06 |
| IL15RA | -1.383209 | 4.4474309 | 103.70651 | 6.998E-22 | 6.914E-19 |
| NCF1C | -1.388472 | 1.064099 | 52.153873 | 2.485E-12 | 7.312E-11 |
| SIGLEC10 | -1.390277 | 2.6861181 | 59.270473 | 1.023E-13 | 5.161E-12 |
| DPYD | -1.390609 | 4.5840222 | 73.984914 | 1.651E-16 | 2.518E-14 |
| MARCO | -1.392803 | 3.5679937 | 21.961719 | 3.782E-06 | 1.827E-05 |
| LYZ | -1.393413 | 6.3523322 | 35.007138 | 6.898E-09 | 6.901E-08 |
| FOLR2 | -1.396382 | 3.9397816 | 49.724692 | 7.477E-12 | 1.859E-10 |
| IL24 | -1.399659 | 1.3831354 | 31.402695 | 3.836E-08 | 3.132E-07 |
| CCL13 | -1.400111 | 2.5519908 | 37.228354 | 2.418E-09 | 2.775E-08 |
| AL021978.1 | -1.401571 | -0.501917 | 47.19192 | 2.376E-11 | 5.018E-10 |
| CD300C | -1.402858 | 0.7245659 | 62.691197 | 2.25E-14 | 1.461E-12 |
| NKG7 | -1.403562 | 3.4030147 | 47.978259 | 1.658E-11 | 3.671E-10 |
| RNASE2 | -1.40546 | -0.01511 | 52.927183 | 1.752E-12 | 5.409E-11 |
| LINC02551 | -1.405884 | -1.085134 | 44.122405 | 9.741E-11 | 1.698E-09 |
| GPR84 | -1.406488 | 1.0452819 | 70.696163 | 6.819E-16 | 7.843E-14 |
| CLDN14 | -1.408056 | -0.628016 | 58.803761 | 1.259E-13 | 6.106E-12 |
| HES2 | -1.413973 | 5.2641045 | 33.916195 | 1.157E-08 | 1.094E-07 |
| C20orf197 | -1.414788 | 0.7109676 | 32.974551 | 1.811E-08 | 1.623E-07 |
| WNT5B | -1.4152 | 4.2399859 | 49.22247 | 9.398E-12 | 2.263E-10 |
| CEACAM21 | -1.416659 | 0.453313 | 62.75982 | 2.183E-14 | 1.432E-12 |
| AC110995.1 | -1.417463 | -0.783479 | 53.781793 | 1.192E-12 | 3.946E-11 |
| LINC02446 | -1.417547 | 0.5016212 | 26.802123 | 3.526E-07 | 2.199E-06 |
| CLCA2 | -1.42137 | 7.227306 | 20.76314 | 6.852E-06 | 3.09E-05 |
| MNDA | -1.423112 | 2.7161543 | 65.793171 | 5.761E-15 | 4.753E-13 |
| ENPP1 | -1.4232 | 3.2136068 | 35.867075 | 4.594E-09 | 4.846E-08 |
| TSPAN7 | -1.423518 | 3.7596683 | 41.103881 | 3.946E-10 | 5.649E-09 |
| MT1X | -1.425003 | 3.7666607 | 58.047114 | 1.763E-13 | 7.958E-12 |
| LILRB2 | -1.427079 | 3.185654 | 60.244157 | 6.639E-14 | 3.662E-12 |
| NOD2 | -1.427487 | 2.4327477 | 74.134695 | 1.548E-16 | 2.397E-14 |
| PSPHP1 | -1.429813 | 2.0117229 | 14.973898 | 0.0001266 | 0.0004075 |
| TYMP | -1.431401 | 7.229326 | 79.589363 | 1.508E-17 | 3.571E-15 |
| FYB1 | -1.432692 | 4.7637449 | 57.252794 | 2.513E-13 | 1.067E-11 |
| SPHK1 | -1.433833 | 5.3669847 | 69.255429 | 1.273E-15 | 1.295E-13 |
| ACP5 | -1.435364 | 6.1684599 | 85.328769 | 1.34E-18 | 4.35E-16 |
| SULT2B1 | -1.435585 | 3.1812324 | 37.242534 | 2.402E-09 | 2.759E-08 |
| FAT2 | -1.436688 | 6.3865543 | 36.378219 | 3.609E-09 | 3.908E-08 |
| BCL2A1 | -1.436815 | 2.3290034 | 57.096588 | 2.695E-13 | 1.135E-11 |
| ADAM23 | -1.438977 | 3.3181647 | 42.284748 | 2.28E-10 | 3.487E-09 |
| LINC01605 | -1.44241 | 1.4150567 | 51.207253 | 3.814E-12 | 1.048E-10 |
| CD300E | -1.44332 | 1.8933388 | 45.245421 | 5.805E-11 | 1.087E-09 |
| AC022126.1 | -1.443985 | -1.015914 | 55.304691 | 6.011E-13 | 2.184E-11 |
| SIGLEC1 | -1.44574 | 4.1738619 | 56.981093 | 2.837E-13 | 1.19E-11 |
| TLL1 | -1.449274 | 1.9208554 | 37.60199 | 2.029E-09 | 2.377E-08 |
| IGHV3-23 | -1.452179 | 5.1442709 | 24.599596 | 1.032E-06 | 5.706E-06 |
| CTSV | -1.452799 | 4.5505762 | 45.00989 | 6.47E-11 | 1.201E-09 |
| MS4A1 | -1.453459 | 3.6830122 | 16.095231 | 7.15E-05 | 0.0002467 |
| SIGLEC12 | -1.453896 | 0.7285759 | 40.406542 | 5.46E-10 | 7.497E-09 |
| HK3 | -1.454361 | 2.8585126 | 59.02629 | 1.14E-13 | 5.612E-12 |
| IGHV3-33 | -1.454586 | 3.2196182 | 23.482561 | 1.786E-06 | 9.303E-06 |
| AL844908.1 | -1.456752 | -1.149734 | 67.595638 | 2.622E-15 | 2.421E-13 |
| S100A8 | -1.457841 | 9.3888032 | 19.40599 | 1.348E-05 | 5.604E-05 |
| FMO2 | -1.458499 | 1.4404456 | 34.583284 | 8.432E-09 | 8.292E-08 |
| IGHV3-74 | -1.458619 | 3.449586 | 30.424641 | 6.13E-08 | 4.73E-07 |
| DSC2 | -1.460252 | 6.6500053 | 48.470316 | 1.324E-11 | 3.036E-10 |
| PDZK1IP1 | -1.460353 | 5.464773 | 29.191412 | 1.109E-07 | 7.967E-07 |
| CLECL1 | -1.467202 | -0.527149 | 48.656452 | 1.216E-11 | 2.831E-10 |
| TREML1 | -1.468562 | -0.725248 | 53.221497 | 1.534E-12 | 4.856E-11 |
| MYOSLID | -1.468815 | 1.0378552 | 30.844513 | 5.011E-08 | 3.95E-07 |
| CFTR | -1.468938 | 1.1855116 | 21.730311 | 4.24E-06 | 2.025E-05 |
| IGHV5-10-1 | -1.471653 | 3.0589548 | 16.533852 | 5.723E-05 | 0.0002029 |
| CPS1 | -1.473747 | 2.2531814 | 36.497476 | 3.412E-09 | 3.73E-08 |
| ALOX12B | -1.478843 | 0.4977783 | 29.892745 | 7.915E-08 | 5.931E-07 |
| ADAMTS15 | -1.48031 | 3.083595 | 52.292226 | 2.334E-12 | 6.96E-11 |
| CD163 | -1.480767 | 5.5495857 | 46.479655 | 3.293E-11 | 6.601E-10 |
| SERPINB1 | -1.483961 | 7.1191448 | 121.01187 | 7.314E-25 | 1.472E-21 |
| BARX2 | -1.485109 | 4.0556813 | 32.462312 | 2.312E-08 | 2.012E-07 |
| AC004847.1 | -1.485881 | -0.085683 | 63.267102 | 1.746E-14 | 1.2E-12 |
| CA2 | -1.487548 | 5.1727209 | 45.717279 | 4.673E-11 | 8.954E-10 |
| MEDAG | -1.490082 | 3.3267667 | 53.152687 | 1.583E-12 | 4.97E-11 |
| GZMH | -1.490216 | 2.0473137 | 44.194881 | 9.42E-11 | 1.656E-09 |
| IGKV3-20 | -1.494528 | 6.3027233 | 26.026555 | 5.142E-07 | 3.071E-06 |
| LINC01705 | -1.498739 | -0.942046 | 39.326181 | 9.041E-10 | 1.164E-08 |
| RNASE7 | -1.503736 | 2.3856646 | 36.36444 | 3.633E-09 | 3.927E-08 |
| LAG3 | -1.504174 | 3.4070413 | 59.158704 | 1.075E-13 | 5.37E-12 |
| IGKV6-21 | -1.504402 | 0.691346 | 20.112816 | 9.471E-06 | 4.11E-05 |
| KLK14 | -1.506221 | 1.6062714 | 24.579852 | 1.042E-06 | 5.755E-06 |
| S100A2 | -1.507768 | 9.8696444 | 34.292067 | 9.681E-09 | 9.387E-08 |
| ARSI | -1.508715 | 3.2349472 | 49.49039 | 8.319E-12 | 2.045E-10 |
| KRT86 | -1.513477 | 2.846153 | 34.93411 | 7.141E-09 | 7.118E-08 |
| CMKLR1 | -1.515245 | 3.6364662 | 66.485539 | 4.256E-15 | 3.725E-13 |
| IGLV1-47 | -1.516841 | 3.9590493 | 28.456861 | 1.581E-07 | 1.087E-06 |
| AL031123.1 | -1.518848 | -0.011788 | 75.745534 | 7.76E-17 | 1.389E-14 |
| PDPN | -1.519206 | 5.7209356 | 74.776892 | 1.175E-16 | 1.923E-14 |
| AC108925.1 | -1.5205 | 0.2314168 | 51.416051 | 3.47E-12 | 9.687E-11 |
| SLC6A14 | -1.523651 | 3.4386319 | 25.061746 | 8.234E-07 | 4.655E-06 |
| C1QC | -1.52384 | 7.0665041 | 66.367172 | 4.482E-15 | 3.872E-13 |
| CXCR2P1 | -1.529247 | 0.8401579 | 33.643255 | 1.317E-08 | 1.232E-07 |
| KIR2DL4 | -1.531458 | -0.294338 | 45.946529 | 4.205E-11 | 8.164E-10 |
| WFDC5 | -1.532632 | 1.4689985 | 26.184296 | 4.762E-07 | 2.863E-06 |
| SDR16C5 | -1.541812 | 2.9287917 | 23.220354 | 2.032E-06 | 1.043E-05 |
| FPR2 | -1.541993 | 0.4668714 | 49.916254 | 6.853E-12 | 1.727E-10 |
| C1QA | -1.543401 | 7.3472226 | 61.903119 | 3.186E-14 | 1.932E-12 |
| MT1L | -1.544404 | 1.795921 | 43.653959 | 1.209E-10 | 2.039E-09 |
| C1QB | -1.549694 | 7.3637618 | 62.591692 | 2.351E-14 | 1.503E-12 |
| VPREB3 | -1.559715 | 1.0103792 | 32.165519 | 2.664E-08 | 2.285E-07 |
| RXFP1 | -1.561432 | -0.042574 | 50.192233 | 6.045E-12 | 1.56E-10 |
| G0S2 | -1.563372 | 4.5836539 | 62.326539 | 2.643E-14 | 1.658E-12 |
| CNFN | -1.572752 | 4.0641448 | 52.212616 | 2.42E-12 | 7.162E-11 |
| AP003071.5 | -1.574845 | 0.2368381 | 48.384284 | 1.377E-11 | 3.133E-10 |
| FCRL5 | -1.575276 | 2.4051567 | 31.218 | 4.19E-08 | 3.392E-07 |
| HMGA2 | -1.58111 | 4.4913575 | 21.042646 | 5.963E-06 | 2.729E-05 |
| TMEM150B | -1.581994 | 0.7859411 | 61.459406 | 3.877E-14 | 2.322E-12 |
| H1-3 | -1.586561 | 0.7946633 | 32.347387 | 2.442E-08 | 2.118E-07 |
| IGLV5-45 | -1.587556 | 1.9615936 | 21.699098 | 4.306E-06 | 2.05E-05 |
| TNFRSF11B | -1.588155 | 3.0207552 | 46.977219 | 2.621E-11 | 5.439E-10 |
| CHI3L2 | -1.589558 | 3.2384322 | 49.93792 | 6.786E-12 | 1.726E-10 |
| SULF2 | -1.590325 | 6.9135766 | 71.206233 | 5.469E-16 | 6.712E-14 |
| SLC22A3 | -1.595211 | 2.320042 | 49.340217 | 8.907E-12 | 2.166E-10 |
| SCAT1 | -1.597971 | 0.5005936 | 51.505194 | 3.332E-12 | 9.356E-11 |
| IGHV3-11 | -1.600264 | 3.6137296 | 27.961929 | 2.009E-07 | 1.346E-06 |
| GRAMD1B | -1.605474 | 2.3019879 | 91.074826 | 1.223E-19 | 5.128E-17 |
| GPR68 | -1.609223 | 4.0920209 | 98.530888 | 5.711E-21 | 3.993E-18 |
| MYO7B | -1.609842 | 1.2208091 | 59.820161 | 8.013E-14 | 4.267E-12 |
| CD14 | -1.612596 | 6.374833 | 81.788796 | 5.943E-18 | 1.617E-15 |
| CNTN5 | -1.613656 | 0.2179883 | 27.586058 | 2.41E-07 | 1.575E-06 |
| VSIG4 | -1.618145 | 4.4144677 | 56.315891 | 3.821E-13 | 1.521E-11 |
| MELTF | -1.621492 | 5.2724617 | 67.137999 | 3.201E-15 | 2.864E-13 |
| H1-5 | -1.629762 | 0.9752392 | 25.26915 | 7.441E-07 | 4.265E-06 |
| CXCL13 | -1.632469 | 4.859429 | 34.361551 | 9.367E-09 | 9.1E-08 |
| FOXC2 | -1.633068 | 0.9260563 | 42.932871 | 1.688E-10 | 2.693E-09 |
| SERPINB3 | -1.636316 | 5.3455088 | 21.172562 | 5.591E-06 | 2.578E-05 |
| GBP5 | -1.636996 | 5.3875165 | 39.89369 | 6.935E-10 | 9.227E-09 |
| PADI4 | -1.638092 | 0.0356871 | 37.842674 | 1.812E-09 | 2.149E-08 |
| SLC12A3 | -1.638456 | 1.0920631 | 25.882396 | 5.516E-07 | 3.268E-06 |
| SLC26A9 | -1.638847 | 0.9167005 | 42.957567 | 1.669E-10 | 2.671E-09 |
| CD109 | -1.639906 | 5.636525 | 76.000506 | 6.958E-17 | 1.297E-14 |
| KRT78 | -1.641567 | 1.4800868 | 41.161099 | 3.842E-10 | 5.524E-09 |
| SERPINB13 | -1.644398 | 4.8588011 | 21.183269 | 5.561E-06 | 2.568E-05 |
| AL354953.1 | -1.647375 | -0.013998 | 59.853471 | 7.896E-14 | 4.216E-12 |
| PPP4R4 | -1.649976 | 0.6407589 | 33.060288 | 1.738E-08 | 1.567E-07 |
| IGKV1-8 | -1.654815 | 0.8618539 | 26.657472 | 3.783E-07 | 2.336E-06 |
| SERPINA1 | -1.657071 | 6.4856228 | 51.436514 | 3.438E-12 | 9.624E-11 |
| PPP1R14C | -1.657285 | 4.0813072 | 49.551749 | 8.089E-12 | 1.993E-10 |
| IL1R2 | -1.658995 | 2.8515098 | 65.821636 | 5.69E-15 | 4.713E-13 |
| LINC00707 | -1.664167 | 0.5978678 | 30.183652 | 6.882E-08 | 5.244E-07 |
| IFNG | -1.668966 | -0.259528 | 42.688664 | 1.89E-10 | 2.977E-09 |
| AC091182.2 | -1.678248 | -0.78577 | 56.128583 | 4.155E-13 | 1.627E-11 |
| CSF3 | -1.680854 | 3.1151484 | 25.945462 | 5.349E-07 | 3.18E-06 |
| CDX1 | -1.693982 | 0.3243278 | 39.845854 | 7.092E-10 | 9.392E-09 |
| STEAP1B | -1.697326 | 1.181009 | 39.981355 | 6.657E-10 | 8.928E-09 |
| LILRA5 | -1.700957 | 1.5180104 | 74.479504 | 1.335E-16 | 2.133E-14 |
| IGLV3-19 | -1.701042 | 4.9910536 | 30.742358 | 5.263E-08 | 4.133E-07 |
| AC015660.1 | -1.701397 | 0.6811837 | 52.848086 | 1.816E-12 | 5.563E-11 |
| KRT77 | -1.702092 | 1.3784828 | 24.137722 | 1.295E-06 | 6.963E-06 |
| MTCO2P12 | -1.703406 | 2.3334854 | 67.471116 | 2.768E-15 | 2.533E-13 |
| ARHGEF4 | -1.704471 | 4.429877 | 69.712632 | 1.044E-15 | 1.101E-13 |
| DSC3 | -1.710279 | 7.4831445 | 28.364437 | 1.654E-07 | 1.131E-06 |
| IGKV1-39 | -1.710568 | 0.6029731 | 30.421852 | 6.138E-08 | 4.734E-07 |
| RFLNA | -1.710708 | 2.1775441 | 43.238469 | 1.465E-10 | 2.395E-09 |
| SDR9C7 | -1.711294 | 1.0130463 | 31.428682 | 3.788E-08 | 3.1E-07 |
| CALHM6 | -1.711555 | 0.3689907 | 52.054455 | 2.599E-12 | 7.604E-11 |
| RGS20 | -1.716518 | 2.0037195 | 75.887711 | 7.302E-17 | 1.324E-14 |
| PKP1 | -1.718806 | 7.839646 | 53.957121 | 1.101E-12 | 3.701E-11 |
| PTPRZ1 | -1.719848 | 3.705863 | 21.913775 | 3.872E-06 | 1.867E-05 |
| MEGF10 | -1.735996 | 0.5265582 | 44.552219 | 7.989E-11 | 1.433E-09 |
| IGHM | -1.736559 | 7.9176455 | 30.201569 | 6.823E-08 | 5.203E-07 |
| CD209 | -1.743084 | 3.0508577 | 65.067093 | 7.918E-15 | 6.178E-13 |
| SAA2 | -1.744341 | 1.9855547 | 31.339416 | 3.954E-08 | 3.214E-07 |
| AL135818.2 | -1.74522 | -1.12759 | 88.885693 | 3.034E-19 | 1.131E-16 |
| KLHDC7B-DT | -1.748369 | -0.519432 | 64.02232 | 1.253E-14 | 9.07E-13 |
| IGHV1-69D | -1.751314 | 3.3959222 | 31.194537 | 4.238E-08 | 3.423E-07 |
| LINC01615 | -1.759239 | 1.6519637 | 58.461603 | 1.466E-13 | 6.879E-12 |
| F3 | -1.761801 | 6.4619089 | 73.960211 | 1.669E-16 | 2.526E-14 |
| TMEM45A | -1.762046 | 6.2947245 | 64.543871 | 9.961E-15 | 7.567E-13 |
| SH3PXD2A-AS1 | -1.768323 | 2.1678855 | 62.976711 | 1.984E-14 | 1.332E-12 |
| LINC02323 | -1.769625 | -0.153306 | 81.117813 | 7.891E-18 | 2.037E-15 |
| CDH3 | -1.770137 | 7.7147719 | 88.142846 | 4.133E-19 | 1.486E-16 |
| ATP6V0D2 | -1.777031 | -0.596373 | 59.58537 | 8.894E-14 | 4.626E-12 |
| AL355916.1 | -1.788117 | 0.7466696 | 77.717417 | 3.343E-17 | 6.867E-15 |
| CXCL10 | -1.796113 | 6.1549222 | 48.544333 | 1.28E-11 | 2.952E-10 |
| AC002401.4 | -1.799765 | 0.9646365 | 51.548032 | 3.268E-12 | 9.227E-11 |
| FIBCD1 | -1.801686 | 1.6677233 | 47.834841 | 1.77E-11 | 3.872E-10 |
| SERPINB4 | -1.801892 | 5.1165689 | 24.531159 | 1.068E-06 | 5.873E-06 |
| AHNAK2 | -1.807402 | 6.3758872 | 55.619258 | 5.22E-13 | 1.928E-11 |
| KRT16P6 | -1.808484 | 3.9097485 | 24.951647 | 8.69E-07 | 4.886E-06 |
| CDHR1 | -1.810089 | 1.3798152 | 56.334057 | 3.79E-13 | 1.519E-11 |
| SYT12 | -1.81537 | 3.4578197 | 65.72171 | 5.944E-15 | 4.844E-13 |
| PGLYRP3 | -1.818573 | 1.8000866 | 31.643609 | 3.418E-08 | 2.83E-07 |
| WARS1 | -1.82115 | 8.2333159 | 127.57362 | 5.754E-26 | 2.317E-22 |
| SHC4 | -1.82126 | 0.6248485 | 93.452452 | 4.579E-20 | 2.144E-17 |
| MT2P1 | -1.821595 | -0.624968 | 73.382793 | 2.139E-16 | 3.032E-14 |
| MMP13 | -1.822665 | 5.7232095 | 34.034047 | 1.094E-08 | 1.044E-07 |
| MT2A | -1.823992 | 8.1940332 | 80.690806 | 9.454E-18 | 2.335E-15 |
| HEPHL1 | -1.831387 | 3.0633066 | 36.360604 | 3.639E-09 | 3.931E-08 |
| AL732437.2 | -1.832963 | -0.447429 | 62.11773 | 2.898E-14 | 1.795E-12 |
| FMO3 | -1.836097 | 2.8601727 | 40.747374 | 4.658E-10 | 6.52E-09 |
| KRT17P3 | -1.83629 | 0.5987315 | 44.287389 | 9.027E-11 | 1.6E-09 |
| VNN1 | -1.84114 | 1.7230451 | 49.921707 | 6.836E-12 | 1.727E-10 |
| LINC02009 | -1.845755 | 0.5044906 | 44.947531 | 6.658E-11 | 1.231E-09 |
| IL20RB | -1.851246 | 4.6384663 | 56.022516 | 4.357E-13 | 1.693E-11 |
| ASPRV1 | -1.854001 | 2.5548981 | 60.427377 | 6.121E-14 | 3.413E-12 |
| GNLY | -1.859384 | 3.8865999 | 66.38036 | 4.456E-15 | 3.866E-13 |
| PPP2R2C | -1.861002 | 3.3650805 | 43.755687 | 1.154E-10 | 1.963E-09 |
| SAA1 | -1.87013 | 5.1826937 | 31.772493 | 3.214E-08 | 2.681E-07 |
| ANKRD1 | -1.878254 | 0.5674322 | 41.085581 | 3.979E-10 | 5.689E-09 |
| CPA4 | -1.879241 | 3.837933 | 42.767583 | 1.822E-10 | 2.882E-09 |
| CHI3L1 | -1.880421 | 6.8201756 | 46.52032 | 3.232E-11 | 6.505E-10 |
| ADRA1B | -1.88044 | -0.306293 | 57.443117 | 2.308E-13 | 9.929E-12 |
| IL12RB2 | -1.903557 | 2.7811709 | 70.809855 | 6.491E-16 | 7.569E-14 |
| IGHV4-4 | -1.909521 | 3.4138637 | 33.045163 | 1.751E-08 | 1.576E-07 |
| IL13RA2 | -1.916296 | 2.8012238 | 43.381601 | 1.372E-10 | 2.263E-09 |
| CCL8 | -1.922012 | 2.116791 | 73.818244 | 1.774E-16 | 2.625E-14 |
| CDA | -1.927822 | 3.5463126 | 82.052677 | 5.316E-18 | 1.507E-15 |
| COL17A1 | -1.931325 | 7.4539434 | 52.623295 | 2.01E-12 | 6.065E-11 |
| AC103718.1 | -1.932928 | 1.9558562 | 62.839436 | 2.108E-14 | 1.401E-12 |
| PICSAR | -1.9341 | 1.7893047 | 33.425653 | 1.461E-08 | 1.347E-07 |
| PTX3 | -1.936146 | 2.731259 | 50.755953 | 4.68E-12 | 1.241E-10 |
| CALB2 | -1.938837 | 2.5611862 | 50.982449 | 4.223E-12 | 1.143E-10 |
| C5AR2 | -1.942064 | 1.6193079 | 95.074035 | 2.35E-20 | 1.183E-17 |
| IGLV2-18 | -1.948075 | 1.4969428 | 33.967288 | 1.129E-08 | 1.072E-07 |
| CXCR1 | -1.957767 | -0.320815 | 60.818884 | 5.147E-14 | 2.96E-12 |
| NOTUM | -1.972583 | 5.2533154 | 24.789241 | 9.408E-07 | 5.249E-06 |
| SPRR2D | -1.973952 | 4.3748022 | 25.816743 | 5.696E-07 | 3.364E-06 |
| CCNA1 | -1.979966 | 1.7782989 | 33.022353 | 1.77E-08 | 1.59E-07 |
| IGKV1-9 | -2.002942 | 4.0909411 | 39.081367 | 1.014E-09 | 1.282E-08 |
| CXCL5 | -2.003131 | 3.4236773 | 39.323544 | 9.053E-10 | 1.164E-08 |
| MMP3 | -2.01345 | 4.6295837 | 45.952423 | 4.194E-11 | 8.152E-10 |
| PTHLH | -2.014613 | 5.1409198 | 50.00851 | 6.572E-12 | 1.677E-10 |
| LVRN | -2.017301 | 1.0029387 | 47.448193 | 2.113E-11 | 4.539E-10 |
| GSDMC | -2.028555 | 3.7249716 | 76.23605 | 6.291E-17 | 1.218E-14 |
| CCL24 | -2.030857 | 1.0998134 | 69.901783 | 9.62E-16 | 1.047E-13 |
| ADH7 | -2.036113 | 4.105763 | 23.823208 | 1.511E-06 | 8.003E-06 |
| IGHV3-48 | -2.053071 | 3.7652583 | 42.028425 | 2.567E-10 | 3.877E-09 |
| KRT16 | -2.061674 | 9.1677328 | 43.747197 | 1.158E-10 | 1.969E-09 |
| MYH16 | -2.074245 | 0.5150136 | 78.891166 | 2.029E-17 | 4.588E-15 |
| L1CAM | -2.08221 | 4.5233421 | 43.591275 | 1.245E-10 | 2.088E-09 |
| KLK7 | -2.101933 | 3.310527 | 25.742388 | 5.906E-07 | 3.474E-06 |
| SPRR2A | -2.114597 | 4.7274198 | 26.813583 | 3.506E-07 | 2.19E-06 |
| MEFV | -2.122185 | 0.7570961 | 113.05862 | 1.665E-23 | 2.394E-20 |
| CXCL11 | -2.127991 | 4.8290241 | 55.673672 | 5.094E-13 | 1.895E-11 |
| ALDH3A1 | -2.128039 | 6.3155576 | 36.653349 | 3.17E-09 | 3.499E-08 |
| HKDC1 | -2.138377 | 1.7370228 | 62.351157 | 2.615E-14 | 1.645E-12 |
| SERPINB7 | -2.148571 | 2.8280691 | 37.801001 | 1.848E-09 | 2.185E-08 |
| KIF19 | -2.149849 | 0.7591838 | 76.426421 | 5.8E-17 | 1.145E-14 |
| HNF4A | -2.161763 | 1.3794443 | 42.978448 | 1.653E-10 | 2.653E-09 |
| AL139351.3 | -2.203353 | -0.334355 | 71.799504 | 4.232E-16 | 5.358E-14 |
| SLURP1 | -2.217259 | 1.4930438 | 42.368688 | 2.193E-10 | 3.367E-09 |
| AKR1B10 | -2.221791 | 6.6316486 | 40.27906 | 5.794E-10 | 7.897E-09 |
| IGLV4-60 | -2.23689 | 1.0960412 | 43.636641 | 1.219E-10 | 2.054E-09 |
| IDO1 | -2.24179 | 5.5664588 | 62.085541 | 2.94E-14 | 1.81E-12 |
| IL36G | -2.251253 | 2.0015806 | 42.806093 | 1.79E-10 | 2.838E-09 |
| AL161431.1 | -2.255199 | 4.6416639 | 37.450208 | 2.179E-09 | 2.537E-08 |
| LAMC2 | -2.256424 | 8.5477026 | 108.75526 | 9.22E-23 | 1.092E-19 |
| LGALS7B | -2.264929 | 3.2984741 | 35.44359 | 5.611E-09 | 5.789E-08 |
| SBSN | -2.270429 | 5.5771846 | 38.714234 | 1.204E-09 | 1.491E-08 |
| LAMA3 | -2.277213 | 7.213649 | 87.876112 | 4.619E-19 | 1.631E-16 |
| ABCA4 | -2.293947 | 3.4941018 | 67.768666 | 2.431E-15 | 2.279E-13 |
| IGHV2-70 | -2.294313 | 2.261696 | 43.012317 | 1.627E-10 | 2.616E-09 |
| KRT81 | -2.299304 | 5.5115542 | 32.578657 | 2.187E-08 | 1.918E-07 |
| NMRAL2P | -2.309005 | 2.4843121 | 40.471831 | 5.296E-10 | 7.307E-09 |
| VSNL1 | -2.312545 | 3.5350387 | 74.289914 | 1.448E-16 | 2.278E-14 |
| GZMA | -2.342963 | 3.7082284 | 94.524297 | 2.946E-20 | 1.412E-17 |
| TMPRSS11A | -2.355775 | 1.7798304 | 44.861961 | 6.926E-11 | 1.266E-09 |
| FLG | -2.392577 | 1.7191745 | 50.185275 | 6.065E-12 | 1.563E-10 |
| TENM2 | -2.393925 | 4.3276322 | 40.584545 | 5.025E-10 | 6.971E-09 |
| GBP6 | -2.396382 | 4.0025562 | 78.793246 | 2.115E-17 | 4.73E-15 |
| KRT5 | -2.40819 | 11.381231 | 49.852774 | 7.054E-12 | 1.766E-10 |
| SH2D5 | -2.429575 | 1.5062368 | 95.179316 | 2.251E-20 | 1.18E-17 |
| TM4SF19 | -2.444801 | -0.037564 | 98.513381 | 5.752E-21 | 3.993E-18 |
| RRAD | -2.446442 | 4.8668789 | 95.696999 | 1.82E-20 | 1.018E-17 |
| S100A7 | -2.470474 | 7.3774649 | 33.254304 | 1.585E-08 | 1.448E-07 |
| IGLV7-43 | -2.47737 | 2.6924646 | 49.932394 | 6.803E-12 | 1.726E-10 |
| MMP9 | -2.488051 | 6.495185 | 73.679249 | 1.883E-16 | 2.728E-14 |
| SPP1 | -2.491073 | 8.4364195 | 92.235046 | 7.567E-20 | 3.312E-17 |
| PLA2G4E | -2.496177 | 2.8880502 | 55.691121 | 5.054E-13 | 1.891E-11 |
| KRT16P2 | -2.515711 | 1.2211595 | 62.656116 | 2.286E-14 | 1.475E-12 |
| SPRR1B | -2.519776 | 6.3958743 | 50.139393 | 6.192E-12 | 1.59E-10 |
| ENTPD8 | -2.573201 | 1.5628896 | 70.341073 | 7.952E-16 | 8.795E-14 |
| NHLH1 | -2.580201 | 0.3454822 | 90.535447 | 1.529E-19 | 6.156E-17 |
| FGFBP1 | -2.584956 | 5.8021642 | 59.633914 | 8.704E-14 | 4.573E-12 |
| CASP14 | -2.613439 | 7.1750213 | 48.082001 | 1.581E-11 | 3.525E-10 |
| RHCG | -2.628006 | 5.9795426 | 70.791644 | 6.543E-16 | 7.569E-14 |
| CES1 | -2.646597 | 6.6491889 | 53.093922 | 1.625E-12 | 5.078E-11 |
| KLK11 | -2.653125 | 2.9771767 | 56.004704 | 4.392E-13 | 1.7E-11 |
| DSG1 | -2.658546 | 4.3019408 | 47.766238 | 1.827E-11 | 3.984E-10 |
| LGALS17A | -2.678542 | 0.4236611 | 73.23661 | 2.278E-16 | 3.184E-14 |
| IBSP | -2.706194 | 1.8227545 | 89.082314 | 2.796E-19 | 1.062E-16 |
| SPIB | -2.734777 | 3.5168733 | 60.225948 | 6.693E-14 | 3.677E-12 |
| KRT6A | -2.759582 | 10.451542 | 48.170961 | 1.518E-11 | 3.403E-10 |
| KLK13 | -2.787685 | 2.2333629 | 56.573069 | 3.405E-13 | 1.39E-11 |
| NTS | -2.879759 | 3.466192 | 57.576704 | 2.175E-13 | 9.538E-12 |
| DSG3 | -2.905471 | 7.056721 | 51.89147 | 2.798E-12 | 8.023E-11 |
| BNC1 | -2.973135 | 3.8821213 | 58.616882 | 1.368E-13 | 6.495E-12 |
| TGM1 | -2.990895 | 4.6533559 | 126.59438 | 8.393E-26 | 2.816E-22 |
| LINC00942 | -3.101023 | 2.8218311 | 83.618348 | 2.748E-18 | 8.256E-16 |
| KRT14 | -3.107833 | 10.263593 | 60.926717 | 4.907E-14 | 2.857E-12 |
| KRT6B | -3.114258 | 7.6953884 | 45.941763 | 4.215E-11 | 8.174E-10 |
| CDH17 | -3.201279 | 2.4855078 | 74.240726 | 1.479E-16 | 2.308E-14 |
| KLK10 | -3.262103 | 3.8351211 | 76.169965 | 6.472E-17 | 1.238E-14 |
| LTF | -3.457173 | 6.8697619 | 71.93356 | 3.994E-16 | 5.121E-14 |
| S100A3 | -3.467486 | 4.8482808 | 136.04153 | 2.266E-27 | 2.737E-23 |
| SLC47A2 | -3.549158 | 1.5493052 | 130.80198 | 1.667E-26 | 1.118E-22 |
| CCER2 | -3.631565 | 2.2209101 | 120.33319 | 9.532E-25 | 1.744E-21 |
| KRT1 | -3.868455 | 6.4388284 | 72.109759 | 3.702E-16 | 4.87E-14 |
| FDCSP | -4.063086 | 5.0155704 | 80.99461 | 8.314E-18 | 2.092E-15 |
| KRT6C | -4.286687 | 5.8538625 | 88.3309 | 3.822E-19 | 1.399E-16 |
|  |  |  |  |  |  |
| **Sheet2 Differentially expressed genes associated with UPK1A-AS1 in UCEC.** | | | | | |
| gene_name | log₂FC | logCPM | F | PValue | padj |
| GFAP | 3.5787957 | 2.3103477 | 164.69602 | 3.338E-33 | 6.275E-29 |
| CALCB | 2.7250237 | 3.2613691 | 70.230619 | 4.337E-16 | 2.145E-13 |
| CALB2 | 2.6031603 | 3.4425786 | 101.38767 | 5.101E-22 | 9.588E-19 |
| LINC02532 | 2.5638322 | 2.0557455 | 105.95888 | 7.297E-23 | 2.743E-19 |
| TF | 2.5182673 | 2.3523833 | 134.93114 | 4.458E-28 | 4.19E-24 |
| EEF1A2 | 2.397599 | 5.5562455 | 50.050435 | 4.528E-12 | 6.755E-10 |
| FLG | 2.3942765 | 1.5534818 | 73.309749 | 1.089E-16 | 6.509E-14 |
| DLL3 | 2.384652 | 1.5377351 | 102.8028 | 2.79E-22 | 8.739E-19 |
| CHGB | 2.2187173 | 2.5714976 | 66.019582 | 2.906E-15 | 1.138E-12 |
| NAPSA | 2.1662656 | 1.9712206 | 82.649949 | 1.722E-18 | 1.618E-15 |
| DHRS2 | 2.1611059 | 4.1124016 | 48.2362 | 1.059E-11 | 1.422E-09 |
| ANO2 | 2.1109062 | 1.4454092 | 132.75846 | 1.077E-27 | 6.747E-24 |
| IGF2BP1 | 2.0656755 | 2.5661728 | 57.049606 | 1.757E-13 | 4.228E-11 |
| IGLV9-49 | 2.0490575 | 3.1526402 | 39.388185 | 6.997E-10 | 5.323E-08 |
| GABRQ | 2.0276372 | 0.9676955 | 86.982742 | 2.572E-19 | 2.844E-16 |
| LBP | 2.0223147 | 3.9834574 | 37.193049 | 2.004E-09 | 1.273E-07 |
| SST | 1.9886627 | 5.434289 | 35.067654 | 5.581E-09 | 3.023E-07 |
| UPK2 | 1.9312911 | 2.4129518 | 72.967636 | 1.269E-16 | 7.119E-14 |
| L1CAM | 1.9086083 | 5.5946213 | 53.258471 | 1.016E-12 | 1.928E-10 |
| TRHDE | 1.8968692 | 1.9671433 | 46.302013 | 2.63E-11 | 3.252E-09 |
| TSPEAR-AS2 | 1.8953568 | 1.3188176 | 62.499771 | 1.441E-14 | 5.017E-12 |
| PNMA2 | 1.8881302 | 0.9845276 | 85.7384 | 4.435E-19 | 4.631E-16 |
| LMO1 | 1.8823252 | 0.9133308 | 59.369553 | 6.041E-14 | 1.67E-11 |
| TGM7 | 1.8369703 | 2.379805 | 46.61688 | 2.267E-11 | 2.841E-09 |
| DUSP9 | 1.8187046 | 1.8194851 | 72.453694 | 1.598E-16 | 8.58E-14 |
| CACNA1B | 1.8160798 | 1.1546593 | 75.799566 | 3.581E-17 | 2.404E-14 |
| CACNB4 | 1.7947356 | 1.2022611 | 101.45558 | 4.955E-22 | 9.588E-19 |
| IRAG2 | 1.7943226 | 2.6392376 | 97.512549 | 2.682E-21 | 4.583E-18 |
| COL4A3 | 1.7826005 | 1.3338258 | 60.800446 | 3.134E-14 | 9.503E-12 |
| PNOC | 1.7787025 | 2.6629119 | 42.237955 | 1.799E-10 | 1.657E-08 |
| RSPO4 | 1.7566694 | 2.2388176 | 49.156916 | 6.879E-12 | 9.87E-10 |
| ALPK2 | 1.7563467 | 0.879138 | 74.192435 | 7.337E-17 | 4.597E-14 |
| FTCD | 1.7466679 | 0.6211955 | 90.208927 | 6.302E-20 | 9.111E-17 |
| GAL | 1.7435565 | 1.8867855 | 60.866301 | 3.041E-14 | 9.371E-12 |
| IGLON5 | 1.7388496 | 1.8266484 | 53.312205 | 9.905E-13 | 1.919E-10 |
| COX4I2 | 1.7283328 | 1.2723227 | 107.10885 | 4.484E-23 | 2.107E-19 |
| FBXO17 | 1.7195027 | 3.4819287 | 101.7718 | 4.33E-22 | 9.588E-19 |
| COL9A1 | 1.7045469 | 4.5261663 | 36.146827 | 3.316E-09 | 1.954E-07 |
| KCNT1 | 1.6976157 | 0.7753757 | 74.335918 | 6.882E-17 | 4.46E-14 |
| FSD1 | 1.6961793 | 1.9939257 | 89.835246 | 7.414E-20 | 9.29E-17 |
| TUBB4A | 1.6946902 | 2.6587 | 56.755799 | 2.011E-13 | 4.555E-11 |
| MYOC | 1.6874596 | 2.1400297 | 57.794099 | 1.246E-13 | 3.124E-11 |
| POU2F3 | 1.6839413 | 1.8146331 | 101.79392 | 4.289E-22 | 9.588E-19 |
| TMPRSS6 | 1.6776744 | 0.9211429 | 61.279487 | 2.517E-14 | 8.02E-12 |
| SLC17A7 | 1.6681373 | 1.5761227 | 59.966195 | 4.594E-14 | 1.349E-11 |
| ZNF334 | 1.6612257 | 1.7349911 | 68.373609 | 1.002E-15 | 4.279E-13 |
| SCN2A | 1.6343287 | 1.5598018 | 47.713215 | 1.354E-11 | 1.767E-09 |
| PHF24 | 1.6211726 | 0.7784937 | 54.613761 | 5.415E-13 | 1.094E-10 |
| HTR6 | 1.6171482 | 1.0268528 | 52.169743 | 1.685E-12 | 3.045E-10 |
| APOA1 | 1.6109907 | 3.9672616 | 28.187091 | 1.595E-07 | 5.222E-06 |
| SIGLEC16 | 1.6047115 | 0.9431594 | 69.118683 | 7.157E-16 | 3.161E-13 |
| AL929091.1 | 1.604325 | -0.105679 | 70.692875 | 3.522E-16 | 1.789E-13 |
| MIR1915HG | 1.6023403 | 1.4381281 | 80.286873 | 4.886E-18 | 4.174E-15 |
| SCN3A | 1.6023308 | 1.4538524 | 40.225027 | 4.691E-10 | 3.817E-08 |
| COL4A4 | 1.5975914 | 2.6475612 | 64.545138 | 5.677E-15 | 2.092E-12 |
| PADI3 | 1.5838918 | 2.1618649 | 40.918722 | 3.37E-10 | 2.892E-08 |
| AC092969.1 | 1.5750802 | 0.8946783 | 38.370052 | 1.139E-09 | 7.815E-08 |
| AOC1 | 1.5739069 | 7.32141 | 35.933729 | 3.675E-09 | 2.138E-07 |
| KCNA5 | 1.5608366 | -0.027474 | 90.035241 | 6.796E-20 | 9.124E-17 |
| IGF2 | 1.5534506 | 4.3538641 | 28.390774 | 1.443E-07 | 4.814E-06 |
| UPK3B | 1.5534082 | 3.6438234 | 52.170269 | 1.684E-12 | 3.045E-10 |
| MTUS2 | 1.5499814 | -0.009885 | 87.322394 | 2.218E-19 | 2.605E-16 |
| H19 | 1.5411254 | 7.140374 | 44.317672 | 6.71E-11 | 7.291E-09 |
| AL117329.1 | 1.5355473 | 1.0783869 | 43.801807 | 8.566E-11 | 8.945E-09 |
| RN7SL3 | 1.525727 | 1.5001254 | 34.266271 | 8.222E-09 | 4.222E-07 |
| HMGA2-AS1 | 1.515208 | 0.1359855 | 59.532389 | 5.606E-14 | 1.596E-11 |
| AP3B2 | 1.5104192 | 1.6004957 | 71.79555 | 2.146E-16 | 1.121E-13 |
| GPRIN2 | 1.5054428 | 1.359632 | 40.383774 | 4.349E-10 | 3.585E-08 |
| KCNJ16 | 1.5016407 | 2.6271344 | 32.900682 | 1.594E-08 | 7.361E-07 |
| SFRP5 | 1.5004387 | 1.9097361 | 32.701693 | 1.756E-08 | 8.056E-07 |
| ZDBF2 | 1.4961117 | 2.2011441 | 63.029579 | 1.132E-14 | 4.014E-12 |
| IGLV3-27 | 1.4925161 | 2.5988431 | 22.904157 | 2.185E-06 | 4.116E-05 |
| PAEP | 1.4895583 | 2.3105928 | 29.085143 | 1.026E-07 | 3.584E-06 |
| PPP2R2B | 1.4880883 | 1.8023872 | 55.802212 | 3.124E-13 | 6.829E-11 |
| SOX11 | 1.4745528 | 2.409911 | 26.370053 | 3.905E-07 | 1.073E-05 |
| MUC3A | 1.4710513 | 1.2999956 | 46.338035 | 2.585E-11 | 3.218E-09 |
| SLC66A1L | 1.4710307 | 0.9995412 | 31.343241 | 3.401E-08 | 1.414E-06 |
| THPO | 1.4704581 | 0.3960064 | 55.619795 | 3.399E-13 | 7.34E-11 |
| TCHH | 1.4618833 | 2.0640125 | 43.112646 | 1.188E-10 | 1.175E-08 |
| LINC00472 | 1.4533398 | 3.7931594 | 58.230091 | 1.02E-13 | 2.662E-11 |
| GPC3 | 1.4468971 | 4.3585325 | 43.484251 | 9.957E-11 | 1.017E-08 |
| SUCNR1 | 1.4391185 | 0.4538326 | 62.081803 | 1.745E-14 | 5.753E-12 |
| FTH1P20 | 1.4366379 | 1.9632124 | 46.197091 | 2.763E-11 | 3.372E-09 |
| LRRN3 | 1.4266516 | 1.3139273 | 48.341712 | 1.008E-11 | 1.383E-09 |
| SLC19A3 | 1.423344 | 1.8974719 | 38.551165 | 1.045E-09 | 7.326E-08 |
| NLGN1 | 1.4214145 | 0.5044575 | 52.219398 | 1.646E-12 | 3.034E-10 |
| CPS1 | 1.4078303 | 2.4963534 | 39.678975 | 6.089E-10 | 4.749E-08 |
| PNMA3 | 1.4067417 | 3.3768398 | 35.873882 | 3.782E-09 | 2.187E-07 |
| IGDCC3 | 1.3800211 | 1.356791 | 44.571601 | 5.951E-11 | 6.541E-09 |
| SCN3B | 1.3755485 | 0.4564337 | 64.708937 | 5.27E-15 | 1.981E-12 |
| SCTR | 1.3727346 | 1.9354612 | 34.835466 | 6.243E-09 | 3.334E-07 |
| ENHO | 1.3721874 | 0.5038567 | 51.831823 | 1.972E-12 | 3.4E-10 |
| LRFN1 | 1.3718042 | 2.9090226 | 78.06053 | 1.31E-17 | 1.026E-14 |
| TCEAL5 | 1.3634968 | 0.732254 | 38.480676 | 1.08E-09 | 7.522E-08 |
| TUBB2B | 1.351454 | 4.3525973 | 32.127146 | 2.322E-08 | 1.017E-06 |
| LRCH2 | 1.3499438 | 1.7531266 | 56.939047 | 1.848E-13 | 4.343E-11 |
| ELOVL4 | 1.3491829 | 1.2817345 | 72.935057 | 1.288E-16 | 7.119E-14 |
| AC021683.1 | 1.3322283 | 0.8341636 | 40.065072 | 5.064E-10 | 4.05E-08 |
| SYT13 | 1.3316595 | 3.5951077 | 35.455572 | 4.628E-09 | 2.551E-07 |
| JPH3 | 1.3312304 | 1.3552712 | 31.75886 | 2.777E-08 | 1.176E-06 |
| ADAMTS9-AS1 | 1.3262466 | 0.2595232 | 48.390907 | 9.85E-12 | 1.361E-09 |
| HMGA2 | 1.3172653 | 5.0857403 | 42.698339 | 1.445E-10 | 1.4E-08 |
| LINC00942 | 1.3172568 | 1.2907739 | 27.866334 | 1.867E-07 | 5.867E-06 |
| STAC2 | 1.3163017 | 3.4190703 | 23.716466 | 1.457E-06 | 2.955E-05 |
| SYCP2 | 1.3153491 | 1.3683155 | 53.137779 | 1.074E-12 | 2.019E-10 |
| DAB1 | 1.3099507 | 0.8434433 | 35.830279 | 3.862E-09 | 2.222E-07 |
| SH3GL2 | 1.3071124 | 0.4727175 | 50.455054 | 3.748E-12 | 5.636E-10 |
| CYS1 | 1.3050865 | 3.9096718 | 31.935953 | 2.548E-08 | 1.098E-06 |
| CRB2 | 1.3024478 | 2.0041649 | 37.722655 | 1.554E-09 | 1.018E-07 |
| HIF3A | 1.2986257 | 3.5351668 | 33.795127 | 1.033E-08 | 5.084E-07 |
| NPY1R | 1.293069 | 1.5427892 | 39.645514 | 6.188E-10 | 4.779E-08 |
| XACT | 1.2909218 | 1.2174826 | 40.872434 | 3.445E-10 | 2.943E-08 |
| NPTX1 | 1.2887505 | 1.3767982 | 39.457023 | 6.771E-10 | 5.173E-08 |
| SCN8A | 1.2858995 | 1.1710933 | 82.830487 | 1.59E-18 | 1.573E-15 |
| RUNDC3A | 1.2843898 | 1.3580267 | 58.536026 | 8.859E-14 | 2.379E-11 |
| VGF | 1.2831807 | 2.6595488 | 26.313272 | 4.016E-07 | 1.096E-05 |
| ESPNL | 1.2826249 | 1.3971103 | 43.217838 | 1.13E-10 | 1.129E-08 |
| UGT3A2 | 1.2812482 | 0.7220916 | 30.688135 | 4.682E-08 | 1.845E-06 |
| ABCC2 | 1.2807114 | 0.8555616 | 61.980119 | 1.827E-14 | 5.922E-12 |
| MUC2 | 1.2767691 | 2.7241486 | 24.032954 | 1.245E-06 | 2.594E-05 |
| ALK | 1.2745478 | 0.6717887 | 40.968567 | 3.291E-10 | 2.837E-08 |
| GALNT9 | 1.2618632 | 0.672187 | 39.642101 | 6.198E-10 | 4.779E-08 |
| SOX8 | 1.2614197 | 0.5054424 | 50.816358 | 3.166E-12 | 4.918E-10 |
| MATN4 | 1.2611005 | 0.2413565 | 53.617422 | 8.596E-13 | 1.701E-10 |
| CNGB1 | 1.2573078 | 0.1901596 | 59.400268 | 5.956E-14 | 1.67E-11 |
| CLDN6 | 1.2531065 | 5.5641633 | 15.623646 | 8.725E-05 | 0.0007889 |
| TRHDE-AS1 | 1.2505478 | 1.898461 | 18.999488 | 1.558E-05 | 0.0001988 |
| ADAMTS16 | 1.2475632 | 2.1871754 | 33.502018 | 1.191E-08 | 5.694E-07 |
| POMC | 1.2472895 | 2.1488768 | 36.025618 | 3.515E-09 | 2.052E-07 |
| SCG2 | 1.2453735 | 1.1519343 | 38.74127 | 9.537E-10 | 6.764E-08 |
| GCK | 1.243651 | 0.5880834 | 36.095437 | 3.399E-09 | 1.997E-07 |
| AACSP1 | 1.2397256 | 1.1208244 | 21.932286 | 3.554E-06 | 6.1E-05 |
| AL133227.1 | 1.2354479 | -0.413175 | 79.061104 | 8.406E-18 | 6.87E-15 |
| SCHIP1 | 1.2322131 | -0.411688 | 69.90758 | 5.016E-16 | 2.357E-13 |
| PPARGC1A | 1.231715 | 1.9862759 | 36.366697 | 2.983E-09 | 1.791E-07 |
| PDZD4 | 1.2245717 | 1.9770135 | 54.81889 | 4.924E-13 | 1.028E-10 |
| FBXO27 | 1.2219854 | 2.805412 | 66.881369 | 1.967E-15 | 8.036E-13 |
| RYR1 | 1.2213189 | 3.3383696 | 51.414105 | 2.396E-12 | 3.849E-10 |
| IGHV5-10-1 | 1.2194781 | 3.4734471 | 13.822119 | 0.0002213 | 0.0016496 |
| FBN3 | 1.2186308 | 4.1157411 | 30.293852 | 5.676E-08 | 2.182E-06 |
| KLRG2 | 1.2164658 | 1.0577087 | 42.841056 | 1.351E-10 | 1.322E-08 |
| APCDD1L-DT | 1.2163983 | 0.6554516 | 30.984143 | 4.052E-08 | 1.645E-06 |
| AC021683.2 | 1.2077978 | 0.9438082 | 35.509004 | 4.51E-09 | 2.515E-07 |
| COL23A1 | 1.2068825 | 4.1246875 | 45.566448 | 3.72E-11 | 4.316E-09 |
| CILP2 | 1.2060335 | 3.3818138 | 56.61657 | 2.145E-13 | 4.8E-11 |
| TMEM145 | 1.2050659 | 0.6021391 | 51.494938 | 2.307E-12 | 3.771E-10 |
| MEG3 | 1.2014686 | 3.6475215 | 47.814102 | 1.291E-11 | 1.697E-09 |
| APCDD1L | 1.2008452 | 1.6057081 | 28.215673 | 1.572E-07 | 5.176E-06 |
| MYOM3 | 1.200065 | 1.228515 | 37.942794 | 1.398E-09 | 9.222E-08 |
| CCKBR | 1.192202 | 1.7442595 | 25.824651 | 5.115E-07 | 1.322E-05 |
| MAGEL2 | 1.1921338 | 0.7217252 | 38.920429 | 8.753E-10 | 6.352E-08 |
| ACTL8 | 1.191496 | 2.2184916 | 17.460362 | 3.407E-05 | 0.0003697 |
| MAPT | 1.1912908 | 1.2197837 | 96.292905 | 4.532E-21 | 7.098E-18 |
| LINC00954 | 1.1876373 | 0.4275867 | 64.777949 | 5.107E-15 | 1.959E-12 |
| LHX2 | 1.1873208 | 1.3911215 | 24.346423 | 1.065E-06 | 2.314E-05 |
| NYAP1 | 1.1779368 | 0.7423698 | 58.146844 | 1.06E-13 | 2.728E-11 |
| ELOVL2 | 1.1776948 | 0.7445231 | 43.725038 | 8.883E-11 | 9.174E-09 |
| BX322639.1 | 1.1769372 | 1.2509233 | 25.665179 | 5.535E-07 | 1.415E-05 |
| IDO2 | 1.1761027 | 0.993403 | 24.769927 | 8.628E-07 | 1.966E-05 |
| CDH17 | 1.1755037 | 1.5423532 | 22.215737 | 3.084E-06 | 5.453E-05 |
| PCSK1N | 1.1747056 | 4.918713 | 35.015614 | 5.723E-09 | 3.091E-07 |
| CNTFR | 1.1708257 | 2.6681202 | 23.52011 | 1.607E-06 | 3.2E-05 |
| HPD | 1.1703938 | 0.3286886 | 51.458163 | 2.347E-12 | 3.803E-10 |
| SDR16C5 | 1.1680126 | 0.9461622 | 20.314294 | 8.016E-06 | 0.0001156 |
| TNNT1 | 1.1620158 | 5.3765741 | 21.298616 | 4.885E-06 | 7.874E-05 |
| S1PR5 | 1.161249 | 0.7645286 | 50.627822 | 3.458E-12 | 5.284E-10 |
| SPTB | 1.1611879 | 1.7751937 | 77.178237 | 1.939E-17 | 1.402E-14 |
| PLAG1 | 1.1592006 | 2.3609914 | 42.312591 | 1.736E-10 | 1.607E-08 |
| KRT81 | 1.1536302 | 2.691773 | 25.944155 | 4.821E-07 | 1.262E-05 |
| LINC00514 | 1.1530505 | 1.5563207 | 33.722332 | 1.07E-08 | 5.199E-07 |
| DGCR5 | 1.151653 | 0.908928 | 40.695702 | 3.748E-10 | 3.145E-08 |
| HOXA2 | 1.1497474 | 1.1156293 | 33.032352 | 1.495E-08 | 6.957E-07 |
| NDUFB4P11 | 1.1463709 | 0.6786256 | 28.69339 | 1.243E-07 | 4.211E-06 |
| WNT7A | 1.1406452 | 6.1671976 | 22.951571 | 2.134E-06 | 4.036E-05 |
| PAK3 | 1.1371773 | 0.2497819 | 40.969971 | 3.289E-10 | 2.837E-08 |
| AL356534.1 | 1.1368228 | -0.496237 | 49.349576 | 6.285E-12 | 9.088E-10 |
| KIF5A | 1.1322948 | 1.6129916 | 51.947246 | 1.869E-12 | 3.283E-10 |
| B4GALNT1 | 1.131877 | 1.4478779 | 48.555033 | 9.12E-12 | 1.27E-09 |
| TCF15 | 1.1299729 | 0.9626834 | 44.73425 | 5.511E-11 | 6.24E-09 |
| SOX2-OT | 1.1288905 | -0.26107 | 50.867143 | 3.092E-12 | 4.843E-10 |
| ACHE | 1.127379 | 1.3403875 | 51.979671 | 1.841E-12 | 3.283E-10 |
| LCNL1 | 1.1238598 | 0.424705 | 29.357388 | 8.977E-08 | 3.233E-06 |
| GRIK5 | 1.121229 | 3.3789418 | 35.809889 | 3.901E-09 | 2.228E-07 |
| TMEM52B | 1.1191877 | -0.027385 | 49.799062 | 5.093E-12 | 7.421E-10 |
| GNG4 | 1.1148993 | 3.6836833 | 28.437224 | 1.41E-07 | 4.716E-06 |
| LINC01936 | 1.1098619 | 0.4286817 | 54.64347 | 5.341E-13 | 1.091E-10 |
| SCGB2A2 | 1.1096499 | 5.9910721 | 9.937121 | 0.0017071 | 0.0084094 |
| NTS | 1.1092838 | 5.5404256 | 8.4232809 | 0.0038514 | 0.0158369 |
| ZNF257 | 1.109251 | 1.1661876 | 33.378243 | 1.264E-08 | 5.985E-07 |
| GRIA4 | 1.1067908 | 0.6093719 | 25.480378 | 6.065E-07 | 1.51E-05 |
| MUC12 | 1.1058579 | 1.3263142 | 26.058086 | 4.557E-07 | 1.208E-05 |
| CLCN3P1 | 1.1035762 | 0.3523395 | 39.115311 | 7.973E-10 | 5.923E-08 |
| ANKLE1 | 1.1004614 | 2.1212418 | 51.807775 | 1.994E-12 | 3.408E-10 |
| DOK5 | 1.0982347 | 1.9269898 | 37.529131 | 1.705E-09 | 1.105E-07 |
| MESP2 | 1.0975434 | 1.1454093 | 37.769302 | 1.52E-09 | 9.988E-08 |
| CCDC144NL-AS1 | 1.0970283 | 2.1806822 | 27.18556 | 2.611E-07 | 7.692E-06 |
| SLC39A5 | 1.0959323 | 1.2268905 | 24.37456 | 1.05E-06 | 2.293E-05 |
| UNC13A | 1.0923375 | 1.3809417 | 33.742244 | 1.06E-08 | 5.174E-07 |
| ZNF114 | 1.0887407 | 2.0795727 | 32.654612 | 1.796E-08 | 8.195E-07 |
| AC144831.1 | 1.0839313 | 1.3153726 | 30.206139 | 5.925E-08 | 2.259E-06 |
| SYP | 1.0831691 | 1.5400995 | 76.339246 | 2.816E-17 | 1.96E-14 |
| TMEM130 | 1.0831598 | 1.7590722 | 56.224276 | 2.571E-13 | 5.685E-11 |
| KCNQ1OT1 | 1.0828043 | 4.5882715 | 37.311479 | 1.893E-09 | 1.211E-07 |
| ADRA2B | 1.0825133 | 0.7861161 | 40.846895 | 3.487E-10 | 2.966E-08 |
| SYN2 | 1.0823912 | 2.3939074 | 26.973649 | 2.899E-07 | 8.36E-06 |
| CHIT1 | 1.0804854 | 3.2125293 | 18.891031 | 1.646E-05 | 0.0002078 |
| GPX3 | 1.0755862 | 8.4940126 | 36.350293 | 3.006E-09 | 1.8E-07 |
| ABCA17P | 1.0729084 | -0.345356 | 38.801877 | 9.264E-10 | 6.621E-08 |
| SPTBN4 | 1.0722224 | 1.5313501 | 62.382365 | 1.521E-14 | 5.197E-12 |
| PCDHB6 | 1.0710555 | 0.8969694 | 44.272094 | 6.857E-11 | 7.407E-09 |
| SEC14L4 | 1.067021 | 0.2197909 | 39.315814 | 7.244E-10 | 5.441E-08 |
| AP003068.4 | 1.066107 | 0.3176192 | 46.005901 | 3.023E-11 | 3.62E-09 |
| KLK6 | 1.0633361 | 4.282393 | 16.843234 | 4.668E-05 | 0.0004746 |
| EPHA10 | 1.0624109 | 0.8853273 | 30.187968 | 5.978E-08 | 2.27E-06 |
| NOVA1 | 1.0606827 | 1.764442 | 37.12251 | 2.073E-09 | 1.308E-07 |
| KRT15 | 1.0599416 | 3.3710931 | 30.333819 | 5.567E-08 | 2.144E-06 |
| MALAT1 | 1.0567734 | 9.3074934 | 42.112212 | 1.91E-10 | 1.726E-08 |
| NPFFR1 | 1.0566083 | 1.6919714 | 44.816965 | 5.299E-11 | 6.037E-09 |
| CLDN11 | 1.0548684 | 1.514602 | 41.67453 | 2.351E-10 | 2.095E-08 |
| CPA3 | 1.0529659 | 1.5681027 | 24.333493 | 1.072E-06 | 2.316E-05 |
| RIC3 | 1.0476643 | 0.1622858 | 29.965173 | 6.666E-08 | 2.481E-06 |
| DNER | 1.0473192 | 2.6872282 | 23.020535 | 2.062E-06 | 3.907E-05 |
| LINC00664 | 1.045471 | 0.6351907 | 41.422686 | 2.651E-10 | 2.317E-08 |
| AC061992.2 | 1.0450214 | 0.216188 | 57.024245 | 1.777E-13 | 4.228E-11 |
| SALL4 | 1.0446441 | 2.6868194 | 35.511534 | 4.504E-09 | 2.515E-07 |
| MIR503HG | 1.043775 | 1.125637 | 38.843426 | 9.081E-10 | 6.53E-08 |
| AC244153.1 | 1.0425028 | 0.4080546 | 40.278628 | 4.573E-10 | 3.737E-08 |
| ANKRD33B | 1.040558 | 2.4952714 | 38.081651 | 1.308E-09 | 8.72E-08 |
| AGT | 1.0367487 | 3.7046106 | 21.947168 | 3.528E-06 | 6.067E-05 |
| CNTN2 | 1.036623 | -0.183614 | 44.135194 | 7.315E-11 | 7.857E-09 |
| GABRE | 1.03373 | 3.1742378 | 39.640038 | 6.204E-10 | 4.779E-08 |
| DUSP26 | 1.0334229 | 0.004561 | 34.521036 | 7.268E-09 | 3.827E-07 |
| YBX2 | 1.0319237 | 3.4771153 | 31.814387 | 2.703E-08 | 1.15E-06 |
| LRRC3 | 1.0317945 | 2.3289061 | 69.095603 | 7.232E-16 | 3.161E-13 |
| AP000662.1 | 1.0312265 | 0.0903012 | 42.452922 | 1.624E-10 | 1.534E-08 |
| GPR158 | 1.031152 | 1.2166054 | 23.3216 | 1.774E-06 | 3.456E-05 |
| NUPR2 | 1.0300864 | 1.0278228 | 20.781619 | 6.335E-06 | 9.688E-05 |
| IGFN1 | 1.0297068 | 2.5179503 | 20.221015 | 8.402E-06 | 0.0001206 |
| CLCNKB | 1.0283311 | 0.6266245 | 42.781229 | 1.39E-10 | 1.353E-08 |
| SLC38A3 | 1.0276967 | 2.1654712 | 26.473525 | 3.711E-07 | 1.03E-05 |
| SLC35F1 | 1.0258376 | 0.2036099 | 42.323151 | 1.727E-10 | 1.607E-08 |
| CELF5 | 1.0249627 | 0.7972199 | 47.125857 | 1.784E-11 | 2.297E-09 |
| GAL3ST3 | 1.0248348 | 1.7994725 | 13.488849 | 0.0002632 | 0.0018895 |
| FST | 1.0220398 | 2.5575248 | 25.01745 | 7.631E-07 | 1.791E-05 |
| MT1H | 1.0220008 | 2.3574941 | 20.480955 | 7.37E-06 | 0.0001087 |
| PCSK1 | 1.0198435 | 1.9735777 | 14.253997 | 0.0001769 | 0.001389 |
| CORO6 | 1.0194301 | 1.9009457 | 31.284258 | 3.5E-08 | 1.449E-06 |
| NOS1 | 1.0186371 | 1.5965104 | 20.575708 | 7.027E-06 | 0.0001046 |
| TFAP2B | 1.0148816 | 1.3026329 | 15.177872 | 0.0001098 | 0.0009446 |
| RDH10-AS1 | 1.0135809 | 1.2896011 | 26.936714 | 2.952E-07 | 8.484E-06 |
| SLC9A3 | 1.0111939 | 1.0232484 | 36.669025 | 2.579E-09 | 1.584E-07 |
| WNK3 | 1.0111179 | 2.1373514 | 39.376093 | 7.038E-10 | 5.323E-08 |
| ATP8A2 | 1.0104795 | 0.5273672 | 39.309559 | 7.266E-10 | 5.441E-08 |
| CEROX1 | 1.0104625 | 0.739543 | 40.16684 | 4.824E-10 | 3.908E-08 |
| TMEM171 | 1.0092523 | -0.136078 | 30.340267 | 5.549E-08 | 2.142E-06 |
| KIAA1549L | 1.0050545 | 2.027746 | 27.015015 | 2.84E-07 | 8.238E-06 |
| LINCR-0001 | 1.004518 | 0.7830703 | 23.035816 | 2.046E-06 | 3.888E-05 |
| CHST13 | 1.0041767 | 0.24303 | 38.044877 | 1.332E-09 | 8.843E-08 |
| PAX2 | 1.0022881 | 5.3823759 | 9.7134772 | 0.0019237 | 0.0092193 |
| SMIM24 | 1.0020956 | 3.7611284 | 25.681635 | 5.49E-07 | 1.41E-05 |
| IGHV2-26 | 1.0003428 | 2.6800224 | 11.110882 | 0.0009153 | 0.0051327 |
| CFAP161 | -1.003159 | 2.2433816 | 20.58806 | 6.983E-06 | 0.0001042 |
| APOD | -1.003989 | 4.8563345 | 22.860852 | 2.233E-06 | 4.189E-05 |
| IGLV3-21 | -1.00551 | 5.3239868 | 14.929083 | 0.0001248 | 0.0010497 |
| WDR49 | -1.005843 | 1.4854467 | 21.204299 | 5.122E-06 | 8.172E-05 |
| AC084866.1 | -1.006198 | 5.0594871 | 14.037534 | 0.0001979 | 0.001514 |
| CDHR3 | -1.00829 | 4.2087183 | 25.248726 | 6.803E-07 | 1.644E-05 |
| ROPN1L | -1.008309 | 4.1169409 | 20.338389 | 7.919E-06 | 0.0001147 |
| IGKV4-1 | -1.019351 | 5.995514 | 15.797487 | 7.979E-05 | 0.000734 |
| SLC25A35 | -1.019751 | 5.830327 | 38.183348 | 1.246E-09 | 8.485E-08 |
| IGHV4-34 | -1.019886 | 3.2654366 | 17.191863 | 3.907E-05 | 0.0004128 |
| C20orf85 | -1.021277 | 5.5136285 | 12.948277 | 0.0003488 | 0.002349 |
| UMODL1-AS1 | -1.02381 | 0.4685069 | 18.043772 | 2.531E-05 | 0.0002915 |
| FAM166B | -1.025246 | 3.9836915 | 22.735563 | 2.377E-06 | 4.39E-05 |
| FRMPD2 | -1.02594 | 1.1148674 | 27.273175 | 2.501E-07 | 7.411E-06 |
| CFAP73 | -1.02747 | 2.6782092 | 24.831084 | 8.37E-07 | 1.926E-05 |
| SCGB1D4 | -1.030915 | 5.5204951 | 7.3831418 | 0.006789 | 0.0247972 |
| GDNF | -1.049286 | 1.0748125 | 20.044686 | 9.184E-06 | 0.0001287 |
| AL357093.2 | -1.050424 | 3.0609309 | 16.225032 | 6.407E-05 | 0.0006163 |
| CIBAR2 | -1.050721 | 3.3478725 | 17.61553 | 3.147E-05 | 0.0003466 |
| IGHV3-73 | -1.053748 | 2.7142537 | 13.113175 | 0.00032 | 0.0022019 |
| IL5RA | -1.056979 | 2.2012364 | 20.333295 | 7.94E-06 | 0.0001148 |
| IGHV3-74 | -1.057059 | 3.1699973 | 19.233022 | 1.384E-05 | 0.0001797 |
| HSPA6 | -1.060521 | 4.1155835 | 24.797951 | 8.509E-07 | 1.948E-05 |
| IGHV3-53 | -1.060693 | 1.7851382 | 17.971051 | 2.626E-05 | 0.0002999 |
| DNAH12 | -1.061046 | 3.1122555 | 22.786363 | 2.318E-06 | 4.309E-05 |
| IGKC | -1.062989 | 11.243458 | 18.762947 | 1.756E-05 | 0.0002184 |
| GAS2L2 | -1.063733 | 3.0674897 | 19.831502 | 1.023E-05 | 0.0001406 |
| RSPH9 | -1.066193 | 1.9995383 | 40.43122 | 4.252E-10 | 3.536E-08 |
| C11orf97 | -1.068169 | 0.6439099 | 18.639913 | 1.87E-05 | 0.0002283 |
| LINC01411 | -1.068894 | 1.4121849 | 19.368977 | 1.292E-05 | 0.0001701 |
| APOBEC4 | -1.072912 | 1.8948905 | 15.95079 | 7.375E-05 | 0.0006892 |
| ANKRD66 | -1.075212 | 1.7997428 | 15.832869 | 7.835E-05 | 0.0007237 |
| TCTE1 | -1.079942 | 1.8198157 | 22.861769 | 2.232E-06 | 4.189E-05 |
| SRGAP3-AS2 | -1.081633 | 2.5797976 | 14.283173 | 0.0001743 | 0.0013756 |
| CDC20B | -1.08306 | 4.4196302 | 16.77863 | 4.825E-05 | 0.0004887 |
| CYP24A1 | -1.083738 | 3.3768304 | 20.601433 | 6.936E-06 | 0.0001039 |
| WDR38 | -1.085377 | 4.0635606 | 19.006106 | 1.553E-05 | 0.0001985 |
| IGHV3-13 | -1.0882 | 0.8370493 | 18.014301 | 2.569E-05 | 0.0002953 |
| ZNF295-AS1 | -1.091999 | 2.8032209 | 28.56834 | 1.322E-07 | 4.462E-06 |
| IGKV1-5 | -1.092294 | 5.8792642 | 18.789889 | 1.733E-05 | 0.0002164 |
| CDHR4 | -1.096379 | 4.4150195 | 16.878626 | 4.585E-05 | 0.0004678 |
| SCGB1A1 | -1.097412 | 2.5836124 | 12.236058 | 0.0005063 | 0.0031793 |
| CXCL5 | -1.098552 | 4.0551464 | 20.694802 | 6.618E-06 | 0.0001002 |
| STOML3 | -1.099843 | 2.4498386 | 17.49701 | 3.343E-05 | 0.0003639 |
| TEKT4 | -1.107094 | 2.4825793 | 22.038062 | 3.371E-06 | 5.836E-05 |
| C1orf189 | -1.110841 | 1.4288319 | 23.495739 | 1.627E-06 | 3.232E-05 |
| TUBA4B | -1.114676 | 4.018668 | 18.65313 | 1.857E-05 | 0.0002274 |
| ERICH3 | -1.126512 | 3.6508953 | 26.067857 | 4.535E-07 | 1.206E-05 |
| ADGB | -1.127277 | 1.3170373 | 20.48556 | 7.353E-06 | 0.0001086 |
| IGHV2-5 | -1.127706 | 3.5485866 | 17.960875 | 2.64E-05 | 0.0003009 |
| C9orf24 | -1.137179 | 4.6755505 | 20.877132 | 6.037E-06 | 9.332E-05 |
| TPPP3 | -1.137606 | 7.3113613 | 29.643919 | 7.802E-08 | 2.836E-06 |
| BPIFB1 | -1.140584 | 3.4210481 | 15.032539 | 0.0001183 | 0.0010052 |
| C4BPB | -1.14325 | 1.4847529 | 26.42881 | 3.793E-07 | 1.047E-05 |
| PI3 | -1.146602 | 6.2624232 | 21.003532 | 5.666E-06 | 8.859E-05 |
| FAM216B | -1.147639 | 3.8563796 | 16.62897 | 5.209E-05 | 0.000518 |
| LINC02345 | -1.170639 | 0.6868453 | 34.583318 | 7.053E-09 | 3.724E-07 |
| IGHG2 | -1.172574 | 8.303895 | 22.414399 | 2.792E-06 | 5.007E-05 |
| NXF3 | -1.173862 | 1.2949475 | 32.066185 | 2.391E-08 | 1.036E-06 |
| DIPK1C | -1.192274 | 0.3153459 | 31.16854 | 3.703E-08 | 1.513E-06 |
| IGLV7-43 | -1.207503 | 1.8580671 | 21.92031 | 3.576E-06 | 6.115E-05 |
| SNTN | -1.207766 | 3.9150475 | 18.5393 | 1.967E-05 | 0.0002381 |
| KRT16 | -1.237629 | 5.4056657 | 22.023431 | 3.395E-06 | 5.871E-05 |
| NAPSB | -1.24975 | 6.2350201 | 28.098287 | 1.666E-07 | 5.37E-06 |
| C1orf87 | -1.250165 | 1.7653502 | 19.393083 | 1.276E-05 | 0.0001685 |
| IGHV3-66 | -1.254983 | 0.9307846 | 25.323028 | 6.557E-07 | 1.607E-05 |
| DTHD1 | -1.257216 | 2.4711031 | 29.007343 | 1.066E-07 | 3.701E-06 |
| OMG | -1.259318 | 3.7905336 | 25.572 | 5.796E-07 | 1.463E-05 |
| LRRC18 | -1.260039 | 0.8515431 | 25.299477 | 6.634E-07 | 1.615E-05 |
| CNGA4 | -1.279421 | 2.1847015 | 30.746348 | 4.551E-08 | 1.804E-06 |
| KLK4 | -1.281596 | 2.8679684 | 19.704806 | 1.09E-05 | 0.000148 |
| SBSN | -1.283977 | 1.8206926 | 28.779157 | 1.192E-07 | 4.052E-06 |
| ALPG | -1.301974 | 6.9537811 | 19.762264 | 1.059E-05 | 0.0001449 |
| SCGB3A1 | -1.318118 | 6.7227839 | 16.500273 | 5.564E-05 | 0.0005484 |
| KRT6A | -1.324631 | 6.0965268 | 21.343696 | 4.775E-06 | 7.718E-05 |
| IGHA2 | -1.325301 | 7.4036839 | 29.959995 | 6.683E-08 | 2.483E-06 |
| IGHGP | -1.340026 | 6.7249348 | 22.96431 | 2.121E-06 | 4.014E-05 |
| C6orf15 | -1.342439 | 3.8205555 | 15.883327 | 7.635E-05 | 0.0007095 |
| TDO2 | -1.364527 | 1.7282282 | 66.702388 | 2.133E-15 | 8.53E-13 |
| AC013724.1 | -1.368321 | 1.9354799 | 22.534262 | 2.629E-06 | 4.756E-05 |
| LY6G6C | -1.378036 | 3.1758512 | 20.082177 | 9.012E-06 | 0.0001271 |
| SERPINA6 | -1.378146 | 3.9353866 | 23.2384 | 1.849E-06 | 3.558E-05 |
| OBP2B | -1.411978 | 4.3053929 | 15.717131 | 8.316E-05 | 0.0007599 |
| C6 | -1.433717 | 2.3601873 | 24.398967 | 1.037E-06 | 2.267E-05 |
| IGHV4-61 | -1.439385 | 1.8563593 | 28.017386 | 1.733E-07 | 5.531E-06 |
| OVGP1 | -1.448242 | 7.2477347 | 24.193503 | 1.149E-06 | 2.429E-05 |
| IGKV1D-8 | -1.45412 | 0.8192419 | 32.332009 | 2.101E-08 | 9.34E-07 |
| IGLV4-69 | -1.489616 | 3.6832897 | 31.904391 | 2.587E-08 | 1.113E-06 |
| PMEL | -1.508267 | 3.1378332 | 77.529965 | 1.658E-17 | 1.247E-14 |
| IGHA1 | -1.508279 | 10.163191 | 40.813989 | 3.542E-10 | 2.999E-08 |
| IGHV5-51 | -1.508343 | 4.9324298 | 31.884857 | 2.612E-08 | 1.117E-06 |
| DNAJC5B | -1.522119 | 0.7169659 | 60.74122 | 3.221E-14 | 9.609E-12 |
| MS4A8 | -1.563367 | 5.1923483 | 27.275138 | 2.498E-07 | 7.411E-06 |
| IGHV3-72 | -1.654249 | 1.0102151 | 49.056076 | 7.212E-12 | 1.027E-09 |
| BTLA | -1.664945 | 0.4964105 | 81.101598 | 3.409E-18 | 3.051E-15 |
| IGHV1-3 | -1.721441 | 3.7099547 | 35.54114 | 4.44E-09 | 2.491E-07 |
| IGLV2-11 | -1.805763 | 4.7887127 | 46.920808 | 1.965E-11 | 2.496E-09 |
| IGLC7 | -1.842888 | 2.0103959 | 44.612815 | 5.836E-11 | 6.491E-09 |
| CSF3 | -1.878568 | 2.7319128 | 51.893125 | 1.916E-12 | 3.335E-10 |
| IGLV4-60 | -1.880603 | 1.636652 | 43.259982 | 1.107E-10 | 1.113E-08 |
| IGHV3-49 | -1.929061 | 3.4542627 | 48.691519 | 8.555E-12 | 1.209E-09 |
| IGLV3-9 | -1.93917 | 2.1910393 | 51.609204 | 2.188E-12 | 3.626E-10 |
| CTSE | -1.980752 | 3.0335041 | 55.556291 | 3.501E-13 | 7.393E-11 |
| MUC5AC | -2.030231 | 5.4763879 | 46.811878 | 2.068E-11 | 2.609E-09 |
| IGLV5-45 | -2.36988 | 3.0541207 | 56.778546 | 1.99E-13 | 4.555E-11 |
| IGLV8-61 | -2.507047 | 3.6746762 | 73.270505 | 1.108E-16 | 6.509E-14 |
| ODAM | -2.558228 | 3.1968447 | 70.136091 | 4.525E-16 | 2.181E-13 |
|  |  |  |  |  |  |
| **Sheet3 Differentially expressed genes associated with UPK1A-AS1 in PRAD.** | | | | | |
| gene_name | log₂FC | logCPM | F | PValue | padj |
| BBOX1 | 3.7777159 | 2.5530916 | 181.82803 | 1.361E-35 | 2.856E-31 |
| SLC6A19 | 3.4141894 | 2.8168504 | 103.41056 | 3.237E-22 | 7.547E-19 |
| CACNA1I | 2.788953 | -0.055061 | 148.94837 | 3.464E-30 | 2.423E-26 |
| CGA | 2.6961504 | 2.5101337 | 73.629806 | 1.178E-16 | 1.124E-13 |
| DAPL1 | 2.391682 | 2.2928472 | 70.059983 | 5.737E-16 | 5.016E-13 |
| SUCNR1 | 2.0871191 | 1.7071341 | 63.965484 | 8.777E-15 | 5.942E-12 |
| ATP12A | 2.0666494 | 2.6513011 | 54.271009 | 7.209E-13 | 2.751E-10 |
| IGHV3-43 | 2.0595108 | -0.214818 | 67.238445 | 2.02E-15 | 1.631E-12 |
| LIPC | 2.0425836 | -0.617611 | 106.63281 | 8.432E-23 | 2.528E-19 |
| GLP1R | 1.9819151 | 1.4393192 | 59.44285 | 6.789E-14 | 3.475E-11 |
| ABCB5 | 1.9022936 | -0.314261 | 81.762236 | 3.33E-18 | 4.368E-15 |
| DHRS2 | 1.6742102 | 2.2666649 | 59.103382 | 7.922E-14 | 3.862E-11 |
| IGHV5-51 | 1.6521897 | 2.828293 | 56.281896 | 2.868E-13 | 1.18E-10 |
| USP32P3 | 1.6225817 | -0.375752 | 121.7314 | 1.702E-25 | 7.143E-22 |
| AC008663.2 | 1.5474921 | -1.143432 | 100.49207 | 1.102E-21 | 2.312E-18 |
| IGHV2-26 | 1.5469381 | 0.0014321 | 40.256748 | 4.972E-10 | 4.831E-08 |
| WFDC5 | 1.5461121 | 1.1934876 | 30.425678 | 5.559E-08 | 2.129E-06 |
| IGHV5-10-1 | 1.5311906 | 0.6749279 | 31.627445 | 3.103E-08 | 1.322E-06 |
| NNAT | 1.5043279 | 2.0013546 | 43.731384 | 9.646E-11 | 1.289E-08 |
| CYP1A1 | 1.4649979 | 0.1814592 | 42.468164 | 1.748E-10 | 2.158E-08 |
| AC009133.3 | 1.448959 | 1.2497583 | 45.025514 | 5.255E-11 | 7.845E-09 |
| SYT4 | 1.435778 | 2.7268695 | 26.713393 | 3.41E-07 | 9.58E-06 |
| MTCO3P12 | 1.4218173 | 3.532941 | 36.912672 | 2.441E-09 | 1.797E-07 |
| IGHV3-73 | 1.4121228 | 0.5017269 | 42.788873 | 1.503E-10 | 1.912E-08 |
| IGKV2D-29 | 1.3829987 | -0.455936 | 35.130802 | 5.73E-09 | 3.435E-07 |
| SPOCK2 | 1.3782702 | 5.6004874 | 84.100739 | 1.206E-18 | 1.808E-15 |
| SGK2 | 1.3411216 | 0.5029304 | 80.320118 | 6.243E-18 | 7.707E-15 |
| AC010255.2 | 1.3030954 | 0.4567331 | 30.600016 | 5.107E-08 | 1.977E-06 |
| OASL | 1.298736 | 1.5379109 | 75.097962 | 6.165E-17 | 6.468E-14 |
| CST2 | 1.2952955 | 4.2138642 | 24.769401 | 8.89E-07 | 2.004E-05 |
| REG4 | 1.2863798 | 4.0795497 | 30.940451 | 4.329E-08 | 1.737E-06 |
| ETV5 | 1.2658455 | 4.05445 | 77.59574 | 2.056E-17 | 2.397E-14 |
| AC061961.1 | 1.2578255 | -0.838719 | 46.456643 | 2.69E-11 | 4.808E-09 |
| LINC02798 | 1.2486241 | -1.116928 | 59.584732 | 6.365E-14 | 3.339E-11 |
| IGLV2-18 | 1.2465006 | -0.348993 | 26.465537 | 3.852E-07 | 1.039E-05 |
| ODAM | 1.2441722 | 0.6024214 | 29.274148 | 9.737E-08 | 3.372E-06 |
| KLF2P4 | 1.2424977 | 0.0324193 | 42.627066 | 1.622E-10 | 2.051E-08 |
| TLCD3B | 1.2349737 | -0.822305 | 58.56153 | 1.014E-13 | 4.727E-11 |
| AP001476.1 | 1.2305537 | -1.184811 | 42.362557 | 1.838E-10 | 2.229E-08 |
| MPPED1 | 1.2098599 | 0.6914122 | 46.783167 | 2.31E-11 | 4.215E-09 |
| AL445985.2 | 1.1835559 | -0.609743 | 43.506662 | 1.072E-10 | 1.406E-08 |
| SPAG17 | 1.1833623 | -0.525676 | 32.338448 | 2.199E-08 | 1.011E-06 |
| IGKV1-27 | 1.1787264 | 0.6332055 | 30.505392 | 5.347E-08 | 2.063E-06 |
| CDH17 | 1.1768206 | 0.9348112 | 41.763017 | 2.438E-10 | 2.707E-08 |
| LINC00514 | 1.1681839 | -1.441657 | 50.906028 | 3.4E-12 | 9.032E-10 |
| IGLV2-14 | 1.1574354 | 3.0742031 | 31.719051 | 2.968E-08 | 1.279E-06 |
| AC018865.2 | 1.1564557 | 1.5709395 | 36.136603 | 3.538E-09 | 2.335E-07 |
| KLF2P3 | 1.1398411 | 0.0154751 | 33.286074 | 1.392E-08 | 6.872E-07 |
| IGHV1-46 | 1.1373937 | 1.2770707 | 28.537285 | 1.395E-07 | 4.554E-06 |
| IGLV3-21 | 1.1332017 | 3.2120597 | 25.153518 | 7.353E-07 | 1.732E-05 |
| C6orf141 | 1.1214218 | -0.652953 | 42.222672 | 1.963E-10 | 2.327E-08 |
| IGHV3-66 | 1.1073366 | -1.136339 | 28.544566 | 1.39E-07 | 4.544E-06 |
| KLK14 | 1.096991 | 2.9004098 | 24.063914 | 1.261E-06 | 2.635E-05 |
| IGHV3-53 | 1.0952494 | 0.1191252 | 24.472997 | 1.029E-06 | 2.257E-05 |
| KLF2P2 | 1.0881735 | -0.976693 | 41.637991 | 2.587E-10 | 2.794E-08 |
| AL136524.1 | 1.0845681 | 0.3989324 | 25.883013 | 5.131E-07 | 1.297E-05 |
| IGHV1-69D | 1.0744006 | 0.8062045 | 24.527791 | 1.002E-06 | 2.204E-05 |
| AMH | 1.0733583 | 1.1218593 | 32.099543 | 2.469E-08 | 1.112E-06 |
| IGHV3-23 | 1.0727369 | 3.0027052 | 26.071337 | 4.677E-07 | 1.21E-05 |
| CEACAM22P | 1.0695531 | 1.5288356 | 17.743518 | 2.996E-05 | 0.0003369 |
| UNC13A | 1.0623863 | 0.0670057 | 41.5129 | 2.744E-10 | 2.908E-08 |
| TRIM31 | 1.0585398 | 0.0432973 | 41.601722 | 2.631E-10 | 2.817E-08 |
| IGKV4-1 | 1.0574269 | 3.7010542 | 26.695082 | 3.441E-07 | 9.629E-06 |
| KLF2P1 | 1.0557524 | 0.2052432 | 30.726182 | 4.804E-08 | 1.883E-06 |
| CALN1 | 1.0471962 | 0.8006066 | 22.768007 | 2.399E-06 | 4.42E-05 |
| DACH2 | 1.0389443 | 0.8512694 | 17.290835 | 3.771E-05 | 0.0004041 |
| MTATP8P2 | 1.0313676 | 0.3436679 | 15.953243 | 7.462E-05 | 0.0006988 |
| KRT4 | 1.0286659 | 2.4516237 | 23.84537 | 1.405E-06 | 2.865E-05 |
| TMPRSS4 | 1.0276298 | 2.0234872 | 35.27083 | 5.357E-09 | 3.268E-07 |
| PDIA2 | 1.0244148 | 2.1832555 | 19.532919 | 1.212E-05 | 0.0001613 |
| FEZF1-AS1 | 1.0243746 | -0.747389 | 32.045938 | 2.534E-08 | 1.124E-06 |
| RUFY4 | 1.0242017 | -0.031555 | 52.420474 | 1.689E-12 | 4.857E-10 |
| IGHV4-34 | 1.0241793 | 1.7215495 | 22.098877 | 3.349E-06 | 5.774E-05 |
| MTCYBP18 | 1.020671 | 0.5494286 | 18.119993 | 2.475E-05 | 0.0002882 |
| AC010624.4 | 1.0165774 | -0.565639 | 19.570636 | 1.189E-05 | 0.0001588 |
| ADAM7 | 1.0101255 | 2.1012679 | 14.114104 | 0.0001922 | 0.0014812 |
| SLC6A4 | 1.0000539 | -0.482122 | 44.390813 | 7.077E-11 | 9.901E-09 |
| DPYS | -1.002413 | 1.9577942 | 22.338002 | 2.972E-06 | 5.264E-05 |
| HAND2-AS1 | -1.012907 | -0.852102 | 27.213027 | 2.668E-07 | 7.823E-06 |
| FP671120.7 | -1.027378 | -0.345008 | 36.463448 | 3.026E-09 | 2.082E-07 |
| MUC6 | -1.049778 | 5.1413294 | 9.1538711 | 0.002609 | 0.0116588 |
| NOTUM | -1.056923 | 0.9319319 | 21.920719 | 3.66E-06 | 6.219E-05 |
| CA3 | -1.065834 | 4.2929413 | 10.415164 | 0.0013314 | 0.0068578 |
| ANKRD2 | -1.070943 | 0.2283045 | 18.553902 | 1.987E-05 | 0.0002402 |
| AD000090.1 | -1.071501 | 1.5931861 | 15.112728 | 0.0001149 | 0.0009834 |
| HAND2 | -1.08509 | -0.793655 | 27.812007 | 1.989E-07 | 6.167E-06 |
| TGM4 | -1.135498 | 8.6348493 | 10.815684 | 0.0010769 | 0.0057888 |
| BCYRN1 | -1.14666 | 0.3329291 | 39.756813 | 6.302E-10 | 5.957E-08 |
| FABP4 | -1.150564 | -0.151516 | 35.043199 | 5.976E-09 | 3.539E-07 |
| AGT | -1.155654 | 5.4743678 | 26.490841 | 3.804E-07 | 1.031E-05 |
| PCSK1 | -1.156973 | -0.120116 | 41.832967 | 2.359E-10 | 2.647E-08 |
| SNORA73B | -1.164276 | 2.1585625 | 19.863559 | 1.026E-05 | 0.000141 |
| DBH | -1.17699 | 0.6475968 | 25.111836 | 7.506E-07 | 1.756E-05 |
| LRRC15 | -1.224289 | 0.0308097 | 36.293752 | 3.282E-09 | 2.214E-07 |
| ZFR2 | -1.22814 | -0.750098 | 38.734644 | 1.024E-09 | 8.808E-08 |
| AC026355.2 | -1.275352 | -0.043718 | 29.415567 | 9.089E-08 | 3.195E-06 |
| ACTA1 | -1.283341 | 5.954957 | 13.411516 | 0.0002766 | 0.0019823 |
| ECEL1 | -1.316608 | 0.3715441 | 46.319738 | 2.868E-11 | 4.974E-09 |
| DPEP1 | -1.468081 | 0.3208223 | 40.963676 | 3.558E-10 | 3.66E-08 |
| MAEL | -1.579393 | -0.071265 | 92.006281 | 4.022E-20 | 6.492E-17 |
| CFC1 | -1.6367 | 0.2463415 | 57.256879 | 1.837E-13 | 7.868E-11 |
| AC012588.1 | -1.873352 | -0.361746 | 96.460236 | 6.046E-21 | 1.153E-17 |
| ACY3 | -2.105568 | 2.0375262 | 60.604729 | 4.007E-14 | 2.336E-11 |
| TRIM63 | -2.371436 | 1.2705132 | 75.45139 | 5.276E-17 | 5.827E-14 |
| TG | -2.540229 | 4.4156955 | 103.5162 | 3.097E-22 | 7.547E-19 |
| IGF2 | -3.060913 | 0.7793244 | 125.74092 | 3.363E-26 | 1.764E-22 |
| ETV4 | -3.529312 | 5.745014 | 115.14908 | 2.497E-24 | 8.732E-21 |
| AC104823.1 | -4.923456 | 3.0288136 | 177.46137 | 6.859E-35 | 7.197E-31 |
|  |  |  |  |  |  |
